# Quantitative Characterization of Macrophage, Lymphocyte, and Neutrophil Subtypes Within the Foreign Body Granuloma of Human Mesh Explants by 5-Marker Multiplex Fluorescence Microscopy

**DOI:** 10.3389/fmed.2022.777439

**Published:** 2022-02-15

**Authors:** Uwe Klinge, Axel Dievernich, Johannes Stegmaier

**Affiliations:** ^1^Department of General, Visceral and Transplant Surgery at the University Hospital of the RWTH Aachen, Aachen, Germany; ^2^Forschungs- und Entwicklungsgesellschaft FEG Textiltechnik, Aachen, Germany; ^3^Institute of Imaging and Computer Vision, RWTH Aachen University, Aachen, Germany

**Keywords:** foreign body granuloma, mesh, multiplex, fluorescence, macrophage, lymphocyte, neutrophil

## Abstract

Foreign bodies such as fibers of a surgical mesh induce a typical reaction with an inflammatory infiltrate that forms a surrounding granuloma. This infiltrate is dominated by macrophages, lymphocytes, and neutrophils, whereas its extent of collaboration is widely unknown. In this study, we analyzed 12 samples of surgical meshes explanted from humans by multiplex analyses with three different 5-marker panels – 1. macrophage panel: CD68, CD86, CD105, CD163, and CD206; 2. lymphocyte panel: CD3, CD4, CD8, CD20, and CD68; and 3. neutrophil panel: CD15, histone, MPO, NE, and CD68. Measurement of fluorescence intensity within nuclear masks resulting from DAPI nuclear staining allows exact quantification of cells considered “positive” at a user-defined mean intensity threshold of > 100. Obviously, however, there is no natural threshold as a biological criterion for an intensity that separates “positive” stained cells from unstained cells (“negative”). Multiplex staining of 5 markers always reveals a high rate of coexpression for almost all of the 2^5^ possible marker combinations (= 32 combinations, when using 5 markers simultaneously). The present staining results demonstrate that various morphological and functional subtypes of macrophages, lymphocytes, and neutrophils are abundant in the foreign body granuloma (FBG), which were investigated by regions of interest (ROI) with an area of 1 mm^2^. The widespread coexpression of two or more markers underscores the complex collaboration network of the inflammatory infiltrate. The ability to combine spatial distribution with exact numerical analysis may offer new perspectives for our understanding of the complex interactions in this multidimensional process.

## Introduction

The repair of hernias as defects of the abdominal wall with possible protrusion of intestine is the most frequent procedure of visceral surgery. Within the past decades, the closure of the hernia orifice turned from simple suture to extended reinforcement with non-absorbable textile structures, the so-called meshes. These porous devices elicit a foreign body reaction that culminates in the formation of a foreign body granuloma (FBG), consisting of an inflammatory infiltrate surrounded by a fibrotic capsule. In some cases, revision surgery is required, mainly because of hernia recurrence, infection, or chronic pain, where these devices with ingrown and adherent tissue need to be removed or replaced.

Image cytometry with the use of various specific antibodies against cellular proteins and staining with several different fluorescent dyes enables the determination of various cell types and their functionality. Measurement of the mean intensities in the area of a nucleus offers the possibility of precise quantification of the number of “positive” cells and thus characterization of the local cellular response to the foreign body.

Quantified analysis of the foreign body reaction at the cell molecular level in terms of precision medicine is essential to determine the different risk profiles of mesh materials.

Macrophages have been shown to be the predominant actors of the chronic inflammation around these foreign bodies, with some of them fusing to multinucleated foreign body giant cells (FBGCs) (Supplementary Figure 1A in Supplementary Material 1). Characteristic surface markers are CD68 as pan-macrophage marker, CD86 for M1 subtype, CD105 indicates macrophage activation, and CD163 and also CD206 reflect M2 subtypes (1). Though these subtypes appear with distinct spatial distribution, they all can be found within a FBG. Recently, it has been shown that lymphocytes may also be the important components of the foreign body reaction (Supplementary Figure 1B in Supplementary Material 1). Corresponding surface markers are CD3 for T-lymphocytes, CD4 for T-helper cells, CD8 for cytotoxic T cells, and CD20 for B-lymphocytes (2). Besides macrophages and lymphocytes, neutrophils have been supposed to contribute to the inflammatory process (Supplementary Figure 1C in Supplementary Material 1), in particular by forming neutrophils extracellular traps (NETs) (3). Characteristic immune markers for NETs are CD15 for neutrophils, and antibodies against myeloperoxidase (MPO), neutrophil elastase (NE), and histone, and in particular the colocalization of all these.

However, it is not clear how many cells of the FBG show these surface markers and if there is some overlapping. As multiplex staining can provide this valuable information, we examined the macrophage pattern, lymphocyte pattern, and neutrophil pattern on 12 explanted mesh samples with 5 markers each and analyzed and quantified their coexpression profiles using the scanning system TissueFAXS PLUS with the StrataQuest Analysis Software from TissueGnostics, Vienna, Austria (4, 5).

## Materials and Methods

We analyzed 12 meshes, all of which have been used for abdominal wall hernia repair in humans: 2 plugs, 1 multifilament polyester (PES) mesh, 2 monofilament polyvinylidene fluoride (PVDF) meshes, 3 polypropylene (PP) meshes, and 4 composite PP meshes, combined with an absorbable part. Meshes with ingrown tissue were removed between 2001 and 2020 because of recurrence, pain, or infection after being incorporated for 2 months to 17 years (Ethics Committee approval: EK 239/19).

Prior to immunofluorescence staining, mesh samples were checked for the presence of mesh and FBGs by hematoxylin and eosin (H&E) with the addition of a polarization filter. By immunohistochemical staining with diaminobenzidine (DAB), we confirmed the functionality and dilution of the antibody markers. All mesh samples showed the typical foreign body reaction around the mesh fibers with an inner layer of inflammatory infiltrate, followed by an outer fibrotic layer. Most specimens showed a varying number of lymphocytes and FBGCs, and also small vessels at the mesh-tissue interface.

### Immunofluorescence Staining

#### General

All steps were performed at room temperature. Serial 2-μm sections of each specimen were labeled with a first marker and subsequently with 4 other markers (Table 1). The “macrophage panel” includes CD68 (pan-macrophage), CD86 (M1), CD105 (activated macrophages), and also CD163 and CD206 both (M2). The “neutrophil panel” includes CD15 (neutrophils), histone H3, MPO, NE, and CD68. The “lymphocyte panel” includes CD3 (pan-T-lymphocyte), CD4 (T-helper cell), CD8 (cytotoxic T cell), CD20 (pan-B-lymphocyte), and CD68 (Supplementary Figure 1 in Supplementary Material 1).

**Table 1:** List of monoclonal antibodies.

**Antibody**	**Clone**	**Dilution**	**Incubation time**	**Manufacturer**	**Host**
**Macrophage panel**					
CD68	KP1	1:6,000	30 min at RT or overnight at 4°C	Dako	Mouse
CD86	BO63	1:200		Novus Biologicals	Mouse
CD105	SN6h	1:25		Dako	Mouse
CD163	5C6FAT	1:800		BMA Biomedicals	Mouse
CD206	15/2	1:200		Origene	Mouse
**Neutrophil panel**					
CD68	KP1	1:6,000	30 min at RT or overnight at 4°C	Dako	Mouse
CD15	I112R.1	1:2,000		Diagnostic BioSystems	Mouse
Histone H3	Polyclonal	1:2,000		Abcam	Rabbit
MPO	EPR20257	1:4,000		Abcam	Rabbit
NE	Polyclonal	1:400		Abcam	Rabbit
**Lymphocyte panel**					
CD3	F7.2.38	1:1,000	30 min at RT or overnight at 4°C	Dako	Mouse
CD4	4B12	1:500		Dako	Mouse
CD8	CD8/144B	1:500		Dako	Mouse
CD20	L26	1:600		Dako	Mouse
CD68	KP1	1:6000		Dako	Mouse

*Monoclonal antibodies used in this study sorted by panel. Additional information: type of clone, dilution, incubation time, manufacturer, and host. MPO, myeloperoxidase; NE, neutrophil elastase; RT, room temperature*.

The order of the fluorophores or fluorescent dyes was always kept the same for all panels; Opal^TM^ 480 was used first, followed by Opal^TM^ 520, Opal^TM^ 570, Opal^TM^ 650, and finally Opal^TM^ 780. All antibodies used were monoclonal and diluted with antibody diluent (with Background Reducing Components, Dako, Germany). Secondary antibodies were applied with ImmPRESS^TM^ HRP (peroxidase) Polymer Detection Kit (Vector, Laboratories, US). Fluorochromes were diluted with 1x Plus Amplification Diluent (PerkinElmer, US).

#### Protocol

Tissue sections with the explanted mesh devices were deparaffinized with xylol, rehydrated through graded alcohol and Milli-Q, before incubation in 3.5% formalin for 10 min. Sections were then placed in a cuvette filled with Milli-Q and pH6 citrate buffer (1:10) and treated with a Decloaking Chamber^TM^ (Biocare Medical, US) for 10 min at 110°C. Afterward, sections were washed with Milli-Q and TBST Tris (buffered saline with Tween 20, Dako) and cooled. Non-specific binding was blocked by incubation with antibody diluent for 10 min.

These steps were followed by incubation with the primary antibody of the first marker. After incubation, sections were rinsed in TBST Tris and incubated with the secondary antibody for 20 min, before applying staining with the Opal^TM^ 480 Reagent Pack (1:100, PerkinElmer) for 10 min. Sections were then washed with TBST Tris and placed in a cuvette filled with AR6 buffer (PerkinElmer) and Milli-Q (1:10). The cuvette was microwave treated for 3 min at 385 W reaching a maximal temperature of 92°C and 15 min at 120 W reaching a maximal temperature of 90°C, before being cooled with cold water. Sections were removed and rinsed with Milli-Q.

Afterward, the primary antibody of the second marker was applied after having blocked again with the antibody diluent for 10 min. Sections were rinsed in TBST Tris and incubated with the secondary antibody for 20 min, before applying staining with the Opal^TM^ 520 reagent pack (1:100, PerkinElmer) for 10 min. Sections were then washed with TBST Tris and placed in a cuvette filled with AR6 buffer (PerkinElmer) and Milli-Q (1:10). The cuvette was microwave treated for 3 min at 385 W reaching a maximal temperature of 92°C and 15 min at 120 W reaching a maximal temperature of 90°C, before being cooled with cold water. Sections were removed and rinsed with Milli-Q.

Subsequent markers were applied the same way as the second marker. After the fifth staining cycle (application of the fifth marker), all tissue sections were mounted with VECTRASHIELD^®^ HardSet^TM^ Antifade Mounting Medium (Vector) with DAPI and coverslipped. The whole staining process for one panel took 3 days in total.

### Analysis of the Fluorescence Images or Stainings

Fluorescence imaging was performed with an Axio Imager 2 microscope (20x, ZEISS, Germany) with an attached Colibri 7 light source (ZEISS, Germany) and the TissueFAXS PLUS system (TissueGnostics, Austria). The light source contains six LED modules and seven fluorescence channels, each producing monochromatic light of a different wavelength. LED-optimized filters and direct coupling increase sensitivity and ensure optimum excitation and emission spectra (Table 2, Supplementary Material 2).

**Table 2:** List of LED modules, filters with excitation and emission [wavelength/FWHM], and staining/fluorophore.

**Marker**	**LED**	**ZEISS filter**	**Excitation**	**Emission**	**Staining/Fluorophore**
Nuclear	UV DAPI	96 HE	390/40	450/40	DAPI
First	Violet	47 Cy	436/25	480/40	Opal^TM^ 480
Second	Green Cy3	46 HE	500/25	535/30	Opal^TM^ 520
Third	Yellow AF594	43 HE	550/25	605/70	Opal^TM^ 570
Fourth	Red Cy5	50 Cy	640/30	690/50	Opal^TM^ 650
Fifth	Far red	Cy 7E	708/75	809/81	Opal^TM^ 780

Images were processed and quantitatively analyzed with StrataQuest Analysis Software (v7, TissueGnostics, Austria). Before applying the analysis app, the minimum and maximum ranges for each filter were set by automatically adjusting the saturation, and the mean minimum and maximum intensities of the slides were used for each marker in each panel.

DAPI images were used to detect and segment nuclei. Nuclei areas were used to measure the mean staining intensities for the five different markers (in six selected circular ROIs with an area of 1 mm^2^). The ROIs were selected such that the mesh fibers were located in the center. We recorded the total number of cells with a mean intensity > 100, considered to be “positive.” The number of “positive” cells was normalized to 2,000 cells (mean of number of cells within the ROIs, reflecting mainly the inflammatory infiltrate of the FBG) for each of the 32 possible combinations of the five markers. Then, the mean of 12 slides each with 6 ROIs was determined. The results of the total of 72 ROIs were compared to the analyses of the entire tissue samples, which were considered mainly as scar tissue.

With a cut-off value of 100 for the mean intensity in the nucleus area for the “positive” cells, the analyses yielded on average <5% “false-positive” cells (Supplementary Material 3).

## Results

### Autofluorescence of HE-Stained Meshes

**Figure 1:**
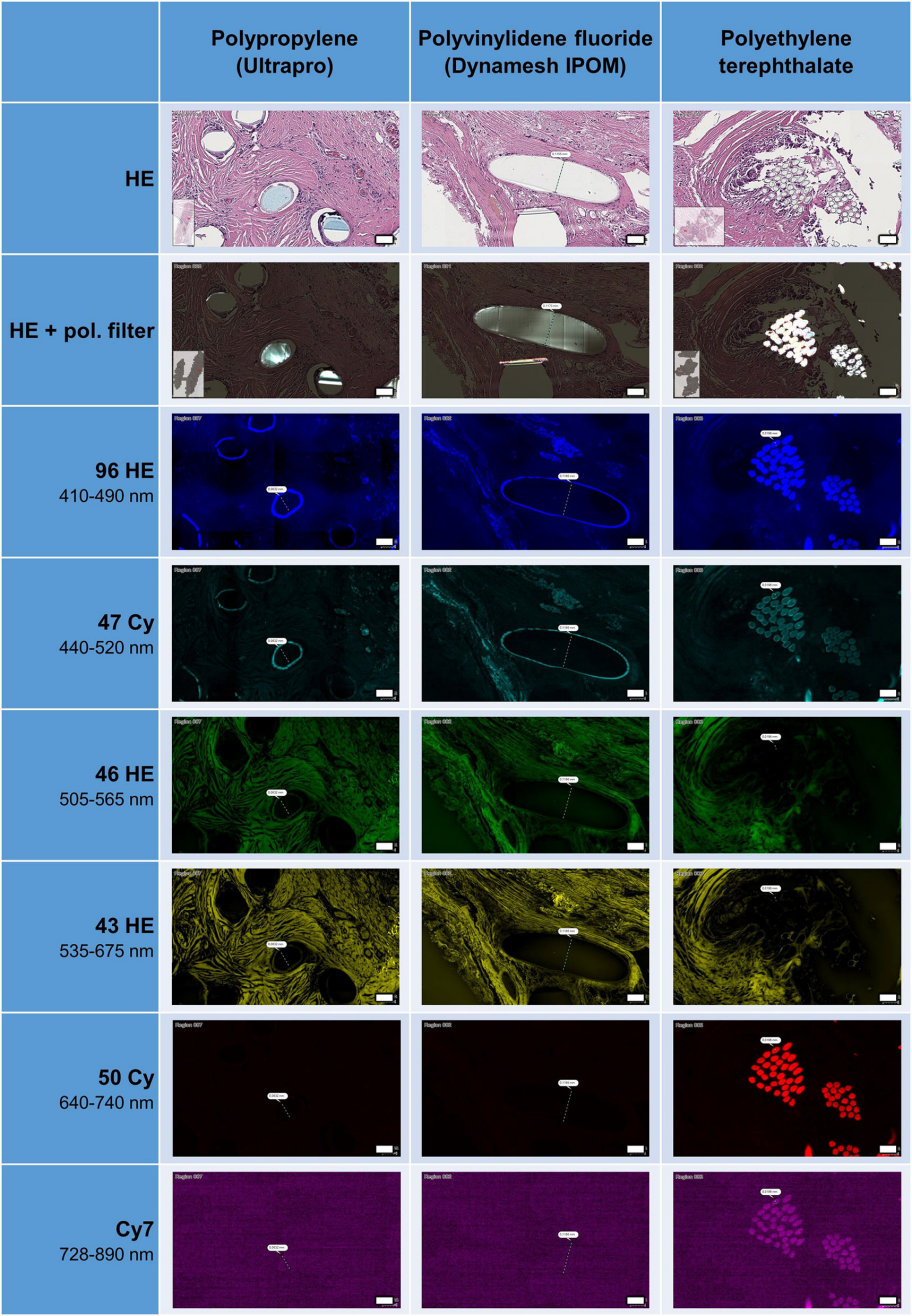
Appearance of HE-stained meshes in bright field microscopy, with or without polarization filter, and with fluorescence microscopy. All polymers visible with pol. filter. Surface of PP and PVDF fibers visible as ring formation with autofluorescence at 410–520 nm, whereas polyester fibers visible at 410–520 and at 640–890 nm. Scale bars = 50 μm.

During the preparation and cutting process of the thin tissue sections (2 μm), most of the polymer fibers were removed, though some fibers remained in all samples (Figure 1). After HE staining, polymer fibers were slightly visible as milky clouds, whereas with the use of a polarization filter, all polymers were hyperintense and could be clearly distinguished from the hypointense surrounding tissues. When using the filters of the fluorescence microscopy, an intrinsic autofluorescence of the fibers became apparent. PP and PVDF showed a marked outer ring formation or “bark” at wavelength of 410–520 nm, in case of the PP with pronounced fragmentation. In contrast, the multifilament PES fibers demonstrated an intense autofluorescence of the entire fiber at both 410–520 nm and 640–890 nm. The illumination of the entire PES fibers is probably due to birefringence within the small (~20 μm) individual fibers.

The frequent fragmentation of the “bark” seen for PP may represent the surface degradation of the fibers seen after incorporation in tissues (6). This fragmentation could also be clearly observed with the use of a polarization filter.

### Multiplex Fluorescence Microscopy With Five Macrophage Cell Markers: CD68, CD86, CD105, CD163, and CD206

**Figure 2:**
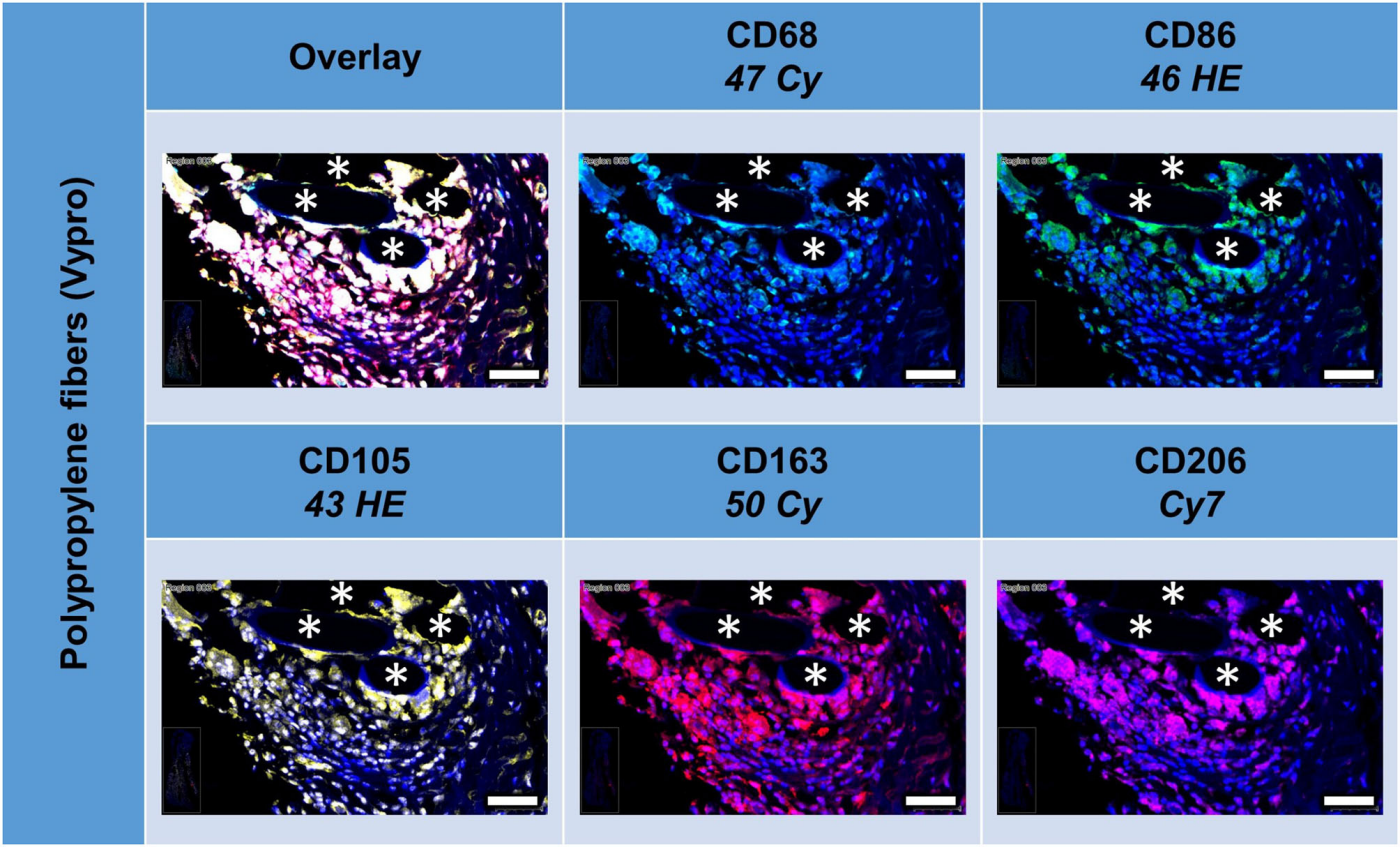
Example of mesh-tissue section stained with macrophage panel. Labeling for nuclei with DAPI (blue), macrophages with CD68 (turquoise), M1 macrophages with CD86 (green), activated macrophages with CD105 (yellow), M2 macrophages with CD163 (red), and CD206 (magenta). Asterisks mark fiber locations, scale bars = 50 μm.

All five markers were detected within the 1 mm^2^ ROIs around mesh fibers that marked the FBG, whereas their spatial heterogeneity confirmed their protein specificity (Figure 2). At first glance, cells in close vicinity to the fiber usually expressed not only a single marker, but often coexpressed multiple macrophage markers, which was confirmed by quantification using a mean nuclear intensity cut-off of 100 (Table 3).

**Table 3:** Comparison of cells located in the FBG and in the scar tissue by means of the macrophage panel.

**Macrophage panel**					**FBG Mean (SD) *n* = 72**	**Scar Mean (SD) *n* = 12**	***t*-test**
**CD68**	**CD86**	**CD105**	**CD163**	**CD206**			
All “positive” cells for a given marker, independent of the other markers (n. d., not defined; pos., positive)							
pos.	n. d.	n. d.	n. d.	n. d.	405 (319)	475 (708)	0.778
n. d.	pos.	n. d.	n. d.	n. d.	418 (325)	266 (211)	^*^0.044
n. d.	n. d.	pos.	n. d.	n. d.	407 (429)	374 (680)	0.823
n. d.	n. d.	n. d.	pos.	n. d.	430 (397)	270 (325)	0.113
n. d.	n. d.	n. d.	n. d.	pos.	266 (260)	76 (55)	^*^0.000
All possible marker combinations (pos., positive; neg., negative)							
neg.	neg.	neg.	neg.	neg.	1,045 (500)	1,387 (895)	0.326
pos.	neg.	neg.	neg.	neg.	71 (89)	184 (308)	0.230
neg.	pos.	neg.	neg.	neg.	58 (99)	37 (41)	0.299
neg.	neg.	pos.	neg.	neg.	57 (109)	78 (176)	0.718
neg.	neg.	neg.	pos.	neg.	111 (149)	57 (68)	^*^0.033
neg.	neg.	neg.	neg.	pos.	43 (86)	18 (20)	0.053
pos.	pos.	neg.	neg.	neg.	69 (118)	57 (68)	0.745
pos.	neg.	pos.	neg.	neg.	17 (41)	43 (120)	0.465
pos.	neg.	neg.	pos.	neg.	10 (13)	8 (7)	0.367
pos.	neg.	neg.	neg.	pos.	5 (13)	2 (2)	0.194
neg.	pos.	pos.	neg.	neg.	14 (31)	9 (8)	0.278
neg.	pos.	neg.	pos.	neg.	6 (7)	7 (7)	0.658
neg.	pos.	neg.	neg.	pos.	10 (26)	2 (3)	^*^0.027
neg.	neg.	pos.	pos.	neg.	52 (101)	52 (127)	0.974
neg.	neg.	pos.	neg.	pos.	12 (36)	7 (11)	0.266
neg.	neg.	neg.	pos.	pos.	27 (60)	5 (5)	^*^0.004
pos.	pos.	pos.	neg.	neg.	54 (88)	52 (83)	0.949
pos.	pos.	neg.	pos.	neg.	18 (29)	11 (13)	0.197
pos.	pos.	neg.	neg.	pos.	11 (18)	2 (2)	^*^0.001
pos.	neg.	pos.	pos.	neg.	20 (65)	39 (118)	0.558
pos.	neg.	pos.	neg.	pos.	1 (3)	1 (2)	0.795
pos.	neg.	neg.	pos.	pos.	5 (10)	1 (1)	^*^0.001
neg.	pos.	pos.	pos.	neg.	17 (42)	9 (8)	0.194
neg.	pos.	pos.	neg.	pos.	8 (20)	2 (3)	^*^0.048
neg.	pos.	neg.	pos.	pos.	5 (16)	1 (2)	0.110
neg.	neg.	pos.	pos.	pos.	21 (47)	6 (6)	^*^0.017
pos.	pos.	pos.	pos.	neg.	39 (54)	50 (72)	0.668
pos.	pos.	pos.	neg.	pos.	22 (39)	4 (5)	^*^0.004
pos.	pos.	neg.	pos.	pos.	19 (34)	3 (4)	^*^0.001
pos.	neg.	pos.	pos.	pos.	6 (14)	2 (3)	0.129
neg.	pos.	pos.	pos.	pos.	25 (56)	4 (5)	^*^0.003
pos.	pos.	pos.	pos.	pos.	62 (81)	15 (13)	^*^0.000

*Mean number of “positive” cells per 2,000 cells. For the FBG, six circular ROIs including mesh fibers were analyzed per sample (n = 12). Comparison with t-test between 280,760 cells located in the FBG and 6,445,165 cells in the whole sample (= scar). Statistically significant differences are marked with asterisks*.

Of the mean 2,000 cells of the ROIs, which included the majority of the FBG, more than 400 cells were either CD68+, CD86+, CD105+, or CD163+, whereas only 206 cells expressed CD206. Almost half of the cells (*n* = 1,045) did not express any of the five markers, whereas 341 showed only one marker, and 614 showed at least some coexpression, and 62 even coexpressed all five markers. Most of the 405 CD68+ cells coexpressed CD86+ (*n* = 294, ~70%), half of them CD105+ (*n* = 221). Coexpression of CD68 and CD163 was seen in 179 cells and CD68 with CD206 in 131 cells.

Comparison of scar tissue vs. the FBG ROIs showed significant differences mainly for CD86+ (*p* < 0.044) and CD206+ (*p* < 0.001) cells, which were predominantly seen within the FBG. Cells coexpressing all five markers were mainly seen in the FBG (62 vs. 15, *p* < 0.001).

**Figure 3:**
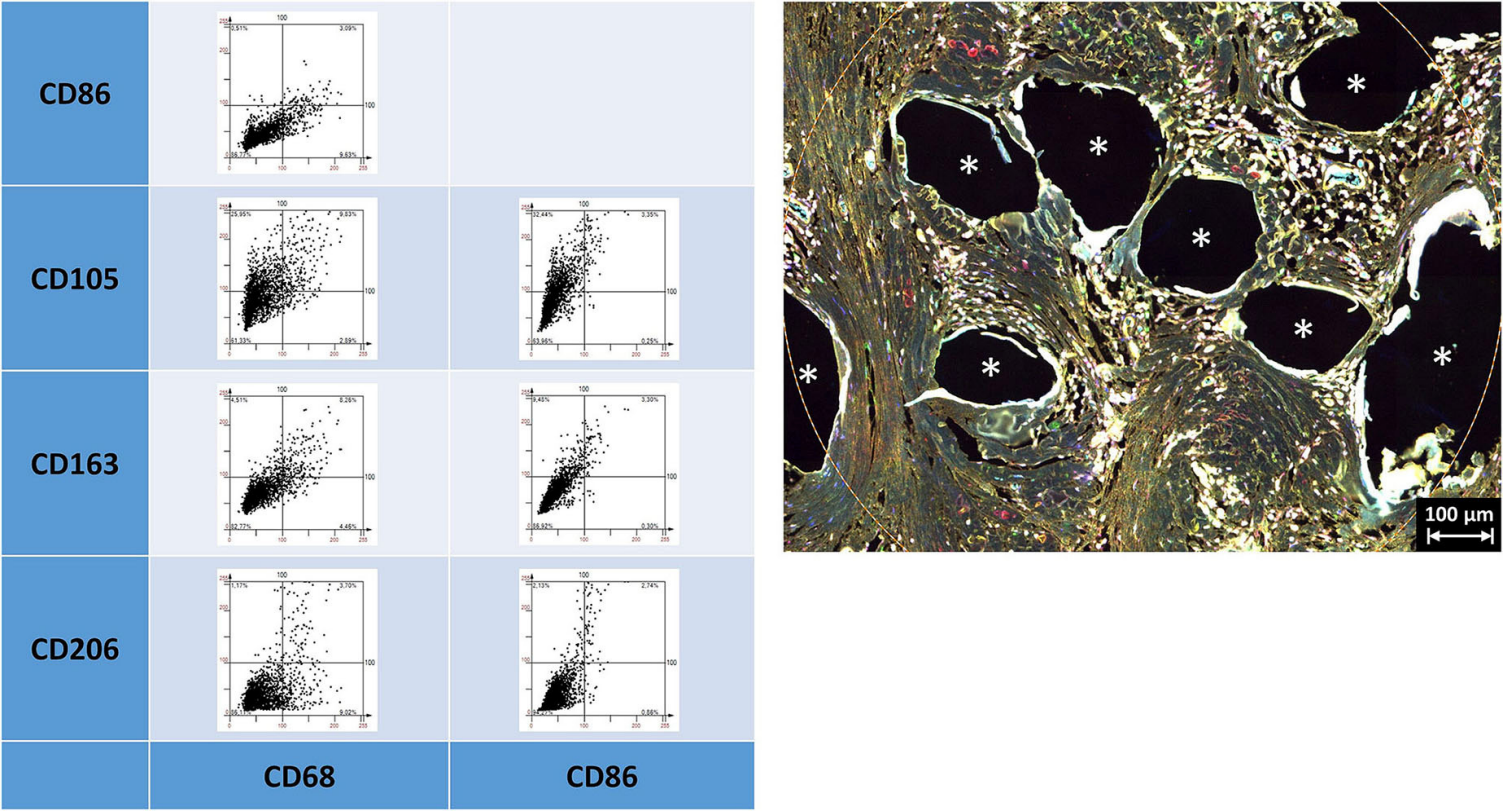
Examples of linear scatter plots from macrophage panel. Scatter plots of mean cellular intensities of two markers in a 1 mm^2^ ROI placed around fibers of a PP mesh plug. Lines in the scatter plots mark the cut-off value 100. Cytometric analysis was performed with StrataQuest 7. Asterisks mark fiber locations.

Depicting the linearly scaled scatter plots for the mean cellular intensities of two markers usually showed a homogenous cloud with a dense cluster marking the background and a continuous transition to the labeled cells without any clear subsets of cell clusters with distinct intensities (Figure 3). Just as little, a clear separation of “positive” cells could not be seen, for any of the five markers (Supplementary Figures 1, 2 in Supplementary Material 4). Noteworthy, the intensity of CD68 correlates with the intensity of CD86 in a linear way as a long oval cloud, and also with CD163, whereas the mean cellular intensities for CD68 and CD105 and also CD68 and CD206 were more distributed at higher intensities. Remarkably, also the intensities of CD86 correlate linearly with CD163 and CD206, which indicate the high plasticity and continuous spectrum of macrophages between the antiinflammatory (M1) and inflammatory (M2) states.

As already indicated by the previous work of Dievernich et al. using double stainings, only a minority of cells that just expressed a single marker can be assigned to a specific cell type (1). As we restricted the analyses just to the inflammatory infiltrate of the granuloma in this study, we did not look for spatial gradients. However, though the markers used were thought to separate the cells into distinct morphological or functional subgroups, the expression profiles of the cells within the FBG were found to be considerably more complex than expected with a high level of interference or coexpression.

### Multiplex Fluorescence Microscopy With Four Lymphocyte Cell Markers: CD3, CD4, CD8, CD20, and CD68 as Reference

**Figure 4:**
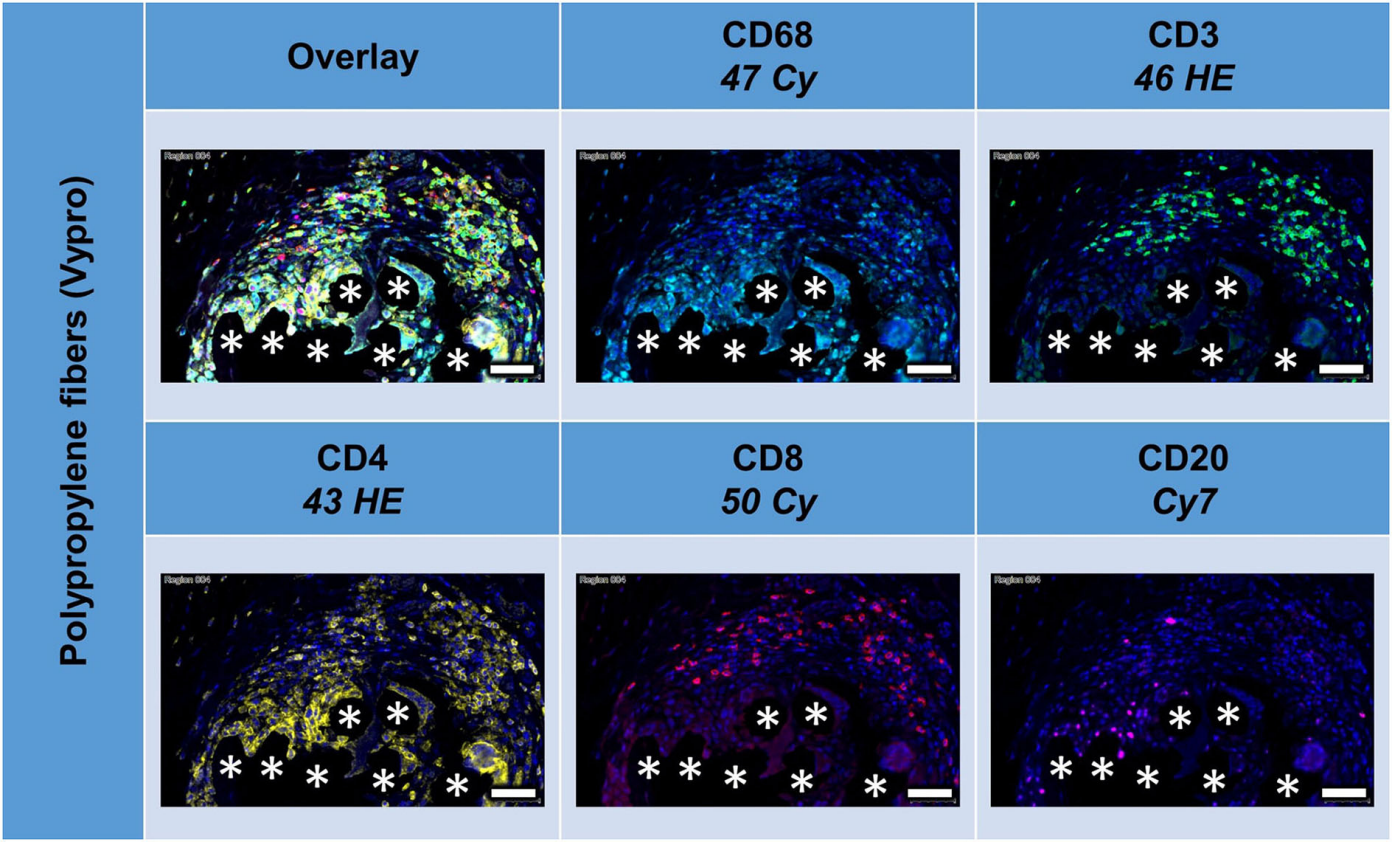
Example of mesh-tissue section stained with lymphocyte panel. Labeling for nuclei with DAPI (blue), macrophages with CD68 (turquoise), T-lymphocytes with CD3 (green), T-helper cells with CD4 (yellow), cytotoxic T cells with CD8 (red), and B-lymphocytes with CD20 (magenta). Asterisks mark fiber locations, scale bars = 50 μm.

All lymphocyte markers were detected within the 1 mm^2^ ROIs around mesh fibers that marked the FBG, but CD20+ cells were mainly seen in clusters outside the FBG (Figure 4). In close vicinity to the fibers, there were predominantly CD68+ cells and CD4+ cells, whereas in a distance of 10 to 20 μm, there were accumulations of CD3+ and CD8+ cells. Single CD20+ cells were distributed equally all over the FBG. Cells in close vicinity to the fiber usually express not only a single marker, but also often coexpressed multiple (Table 4).

**Table 4:** Comparison of cells located in the FBG and in the scar tissue by means of the lymphocyte panel.

**Lymphocyte panel**					**FBG Mean (SD) *n* = 72**	**Scar Mean (SD) *n* = 12**	***t*-test**
**CD68**	**CD3**	**CD4**	**CD8**	**CD20**			
All “positive” cells for a given marker, independent of the other markers (n. d., not defined; pos., positive)							
pos.	n. d.	n. d.	n. d.	n. d.	360 (287)	183 (156)	^*^0.004
n. d.	pos.	n. d.	n. d.	n. d.	304 (288)	124 (101)	^*^0.000
n. d.	n. d.	pos.	n. d.	n. d.	345 (289)	169 (160)	^*^0.005
n. d.	n. d.	n. d.	pos.	n. d.	395 (399)	288 (217)	0.185
n. d.	n. d.	n. d.	n. d.	pos.	93 (193)	31 (38)	^*^0.015
All possible marker combinations (pos., positive; neg., negative)							
neg.	neg.	neg.	neg.	neg.	1,104 (523)	1,803 (1,445)	0.174
pos.	neg.	neg.	neg.	neg.	119 (111)	58 (57)	^*^0.008
neg.	pos.	neg.	neg.	neg.	48 (60)	21 (17)	^*^0.004
neg.	neg.	pos.	neg.	neg.	58 (101)	34 (43)	0.242
neg.	neg.	neg.	pos.	neg.	115 (144)	136 (137)	0.680
neg.	neg.	neg.	neg.	pos.	38 (85)	14 (17)	^*^0.031
pos.	pos.	neg.	neg.	neg.	12 (25)	2 (3)	^*^0.006
pos.	neg.	pos.	neg.	neg.	59 (88)	21 (25)	^*^0.006
pos.	neg.	neg.	pos.	neg.	39 (66)	40 (51)	0.986
pos.	neg.	neg.	neg.	pos.	1 (4)	0 (1)	0.072
neg.	pos.	pos.	neg.	neg.	32 (42)	18 (19)	0.070
neg.	pos.	neg.	pos.	neg.	45 (64)	20 (19)	^*^0.011
neg.	pos.	neg.	neg.	pos.	12 (32)	3 (5)	^*^0.045
neg.	neg.	pos.	pos.	neg.	25 (65)	13 (17)	0.145
neg.	neg.	pos.	neg.	pos.	3 (7)	1 (1)	^*^0.012
neg.	neg.	neg.	pos.	pos.	3 (12)	2 (3)	0.185
pos.	pos.	pos.	neg.	neg.	19 (30)	5 (5)	^*^0.003
pos.	pos.	neg.	pos.	neg.	20 (54)	3 (4)	^*^0.022
pos.	pos.	neg.	neg.	pos.	0 (1)	0 (0)	0.363
pos.	neg.	pos.	pos.	neg.	34 (55)	26 (37)	0.481
pos.	neg.	pos.	neg.	pos.	0 (1)	0 (0)	0.099
pos.	neg.	neg.	pos.	pos.	0 (1)	0 (0)	0.896
neg.	pos.	pos.	pos.	neg.	33 (55)	17 (22)	0.122
neg.	pos.	pos.	neg.	pos.	16 (34)	4 (7)	^*^0.015
neg.	pos.	neg.	pos.	pos.	6 (20)	2 (3)	0.105
neg.	neg.	pos.	pos.	pos.	2 (7)	0 (1)	0.075
pos.	pos.	pos.	pos.	neg.	60 (80)	25 (32)	^*^0.018
pos.	pos.	pos.	neg.	pos.	1 (1)	0 (1)	0.371
pos.	pos.	neg.	pos.	pos.	0 (0)	0 (0)	0.635
pos.	neg.	pos.	pos.	pos.	0 (1)	0 (0)	0.310
neg.	pos.	pos.	pos.	pos.	7 (25)	2 (4)	0.127
pos.	pos.	pos.	pos.	pos.	1 (4)	1 (3)	0.618

*Mean number of “positive” cells per 2,000 cells. For the FBG, six circular ROIs including mesh fibers were analyzed per sample (n = 12). Comparison with t-test between 234,177 cells located in the FBG and 5,775,972 cells in the whole sample (= scar). Statistically significant differences are marked with asterisks*.

Of the mean 2,000 cells of the sphere in the FBG, more than 300 cells were CD68+, CD3+, CD4+, or CD8+, and only CD20+ cells were markedly less with 93 cells. CD8+ cells were most common. A total of 148 cells were even “double-positive” for CD4 and CD8 (CD4+CD8+ cells). Half of the CD8+ cells costained for CD3, but only 25% of the CD4+ cells. Of 395 CD8+ cells, there are only 115 “single-positive” cells, 45 are CD8+CD3+, 39 are CD8+CD68+, and 20 are CD3+CD8+CD68+. Of 345 CD4+ cells, there are 58 exclusively positive for CD4, 32 are CD4+CD3+, 59 are CD4+CD68+, and 19 are CD3+CD4+CD68+. Altogether, among the CD4+ cells, in the mean per 2,000 cells, there were 93 that were CD68+ but not CD3+, 88 were CD3+ but not CD68+, and 81 were positive for both CD3 and CD68 (Table 4).

Comparing the scar area vs. ROIs within the FBG showed significant differences for CD68+ (*p* < 0.01), CD3+ (*p* < 0.001), CD4+ (*p* < 0.01), and CD20+ (*p* = 0.015) cells, which all were predominantly seen in the FBG, but no differences for CD8+ cells. Cells coexpressing all five markers were almost absent (Table 4).

**Figure 5:**
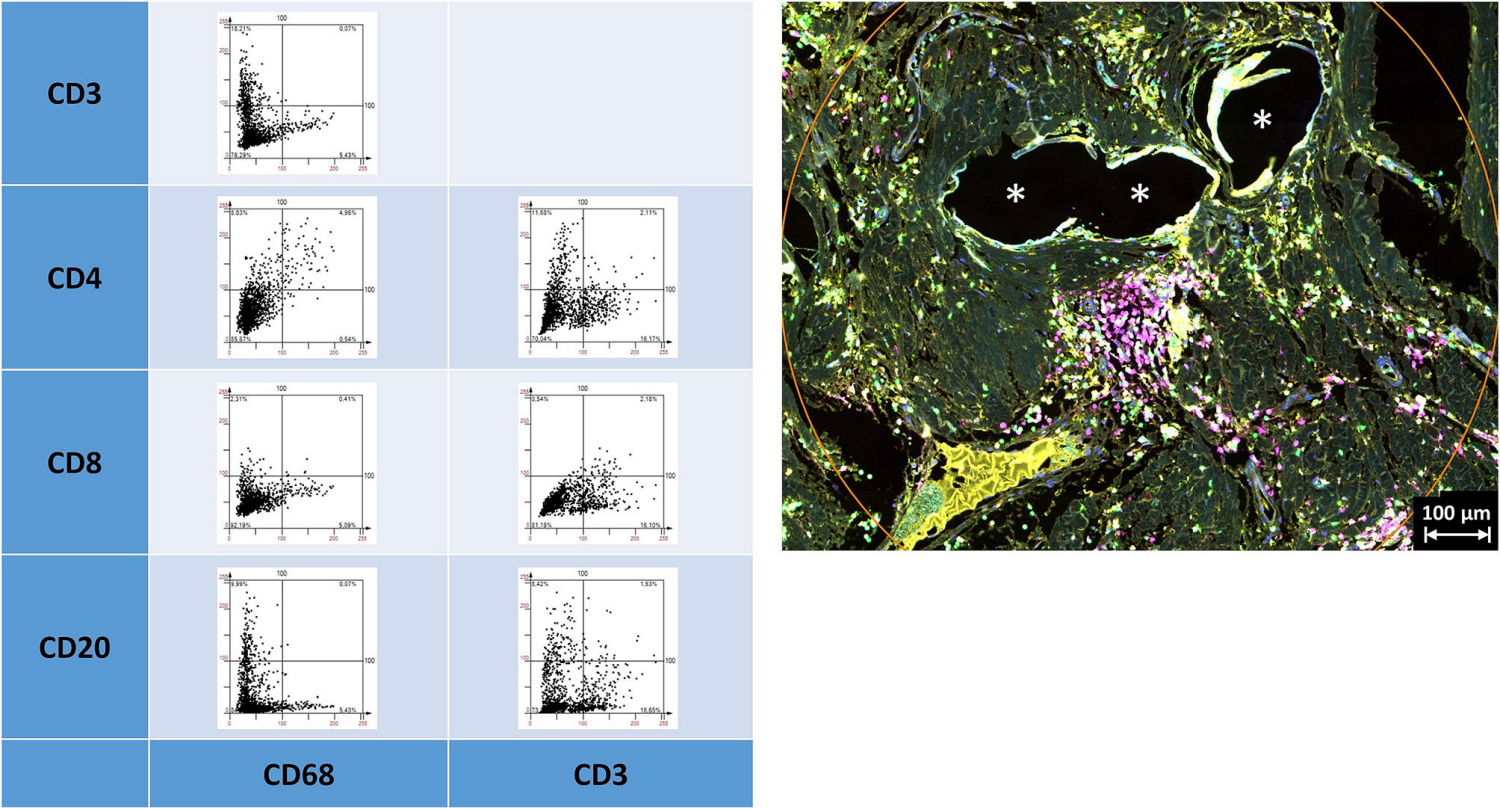
Examples of linear scatter plots from lymphocyte panel. Scatter plots of mean cellular intensities of two markers in a 1 mm^2^ ROI placed around PP mesh fibers. Lines in the scatter plot mark the cut-off value 100. Cytometric analysis was performed with StrataQuest 7. Asterisks mark fiber locations.

Depicting the linearly scaled scatter plots for the intensities of two markers in the lymphocyte panel showed a clearer separation for CD68+ and CD3+, CD68+ and CD8+, and CD68+ and CD20+, and also CD3+ and CD4+ and CD3+ and CD20+ cells compared to the marker combinations of the macrophage panel, as indicated by an “L” configuration. However, coexpressing cells were also present (Figure 5). As for the macrophage marker, a clear separation of “positive” cells could not be seen.

### Multiplex Fluorescence Microscopy With Four Neutrophil Cell Markers: CD15, Histone, MPO, and NE

**Figure 6:**
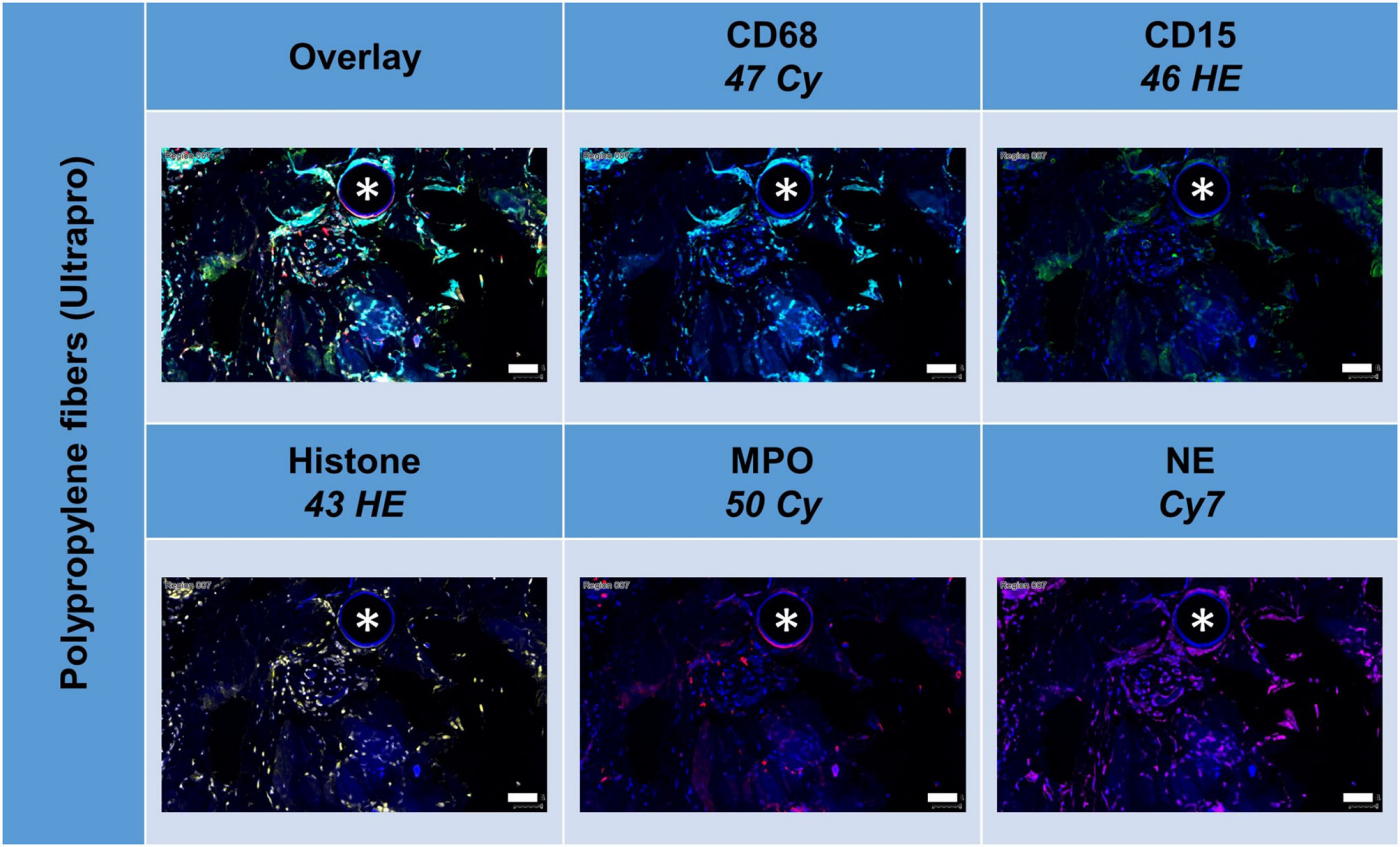
Example of mesh-tissue section stained with neutrophil panel. Labeling for nuclei with DAPI (blue), macrophages with CD68 (turquoise), neutrophils with CD15 (green), histone (yellow), myeloperoxidase (MPO, red), and neutrophil elastase (NE, magenta). Asterisk marks a fiber location, scale bars = 50 μm.

All these markers were detected within the ROIs (Figure 6, Table 5). Whereas, histone usually appeared within the area of the nuclear mask, CD15, MPO, and NE were often found in the extranuclear area, too. CD15+ (*p* = 0.010) and histone+ (*p* = 0.049) cells were seen significantly more often in the FBG compared to the general scar tissue.

**Table 5:** Comparison of cells located in the FBG and in the scar tissue by means of the neutrophil panel.

**Neutrophil panel**					**FBG Mean (SD) *n* = 72**	**Scar Mean (SD) *n* = 12**	***t*-test**
**CD68**	**CD15**	**Histone**	**MPO**	**NE**			
All “positive” cells for a given marker, independent of the other markers (n. d., not defined; pos., positive)							
pos.	n. d.	n. d.	n. d.	n. d.	374 (272)	516 (499)	0.354
n. d.	pos.	n. d.	n. d.	n. d.	264 (323)	144 (86)	^*^0.010
n. d.	n. d.	pos.	n. d.	n. d.	250 (345)	135 (137)	^*^0.049
n. d.	n. d.	n. d.	pos.	n. d.	300 (347)	241 (249)	0.485
n. d.	n. d.	n. d.	n. d.	pos.	262 (315)	204 (175)	0.363
All possible marker combinations (pos., positive; neg., negative)							
neg.	neg.	neg.	neg.	neg.	1,242 (444)	2,069 (1,802)	0.141
pos.	neg.	neg.	neg.	neg.	111 (112)	290 (353)	0.109
neg.	pos.	neg.	neg.	neg.	48 (64)	24 (15)	^*^0.006
neg.	neg.	pos.	neg.	neg.	59 (107)	30 (37)	0.083
neg.	neg.	neg.	pos.	neg.	58 (80)	67 (91)	0.764
neg.	neg.	neg.	neg.	pos.	100 (178)	82 (88)	0.586
pos.	pos.	neg.	neg.	neg.	34 (53)	33 (39)	0.929
pos.	neg.	pos.	neg.	neg.	13 (22)	9 (12)	0.462
pos.	neg.	neg.	pos.	neg.	23 (31)	53 (111)	0.362
pos.	neg.	neg.	neg.	pos.	22 (39)	26 (32)	0.663
neg.	pos.	pos.	neg.	neg.	6 (9)	2 (3)	^*^0.010
neg.	pos.	neg.	pos.	neg.	35 (65)	15 (14)	^*^0.028
neg.	pos.	neg.	neg.	pos.	5 (9)	2 (3)	^*^0.028
neg.	neg.	pos.	pos.	neg.	15 (32)	6 (9)	0.052
neg.	neg.	pos.	neg.	pos.	31 (51)	20 (26)	0.285
neg.	neg.	neg.	pos.	pos.	5 (12)	5 (3)	0.898
pos.	pos.	pos.	neg.	neg.	8 (22)	3 (4)	0.118
pos.	pos.	neg.	pos.	neg.	39 (99)	24 (25)	0.282
pos.	pos.	neg.	neg.	pos.	5 (10)	3 (4)	0.262
pos.	neg.	pos.	pos.	neg.	17 (39)	8 (13)	0.131
pos.	neg.	pos.	neg.	pos.	10 (21)	11 (19)	0.854
pos.	neg.	neg.	pos.	pos.	10 (27)	13 (14)	0.551
neg.	pos.	pos.	pos.	neg.	8 (18)	4 (6)	0.099
neg.	pos.	pos.	neg.	pos.	2 (4)	1 (1)	0.055
neg.	pos.	neg.	pos.	pos.	3 (7)	2 (1)	0.098
neg.	neg.	pos.	pos.	pos.	5 (12)	3 (3)	0.222
pos.	pos.	pos.	pos.	neg.	23 (48)	9 (16)	0.054
pos.	pos.	pos.	neg.	pos.	4 (9)	2 (2)	0.062
pos.	pos.	neg.	pos.	pos.	11 (28)	7 (8)	0.373
pos.	neg.	pos.	pos.	pos.	15 (30)	13 (25)	0.757
neg.	pos.	pos.	pos.	pos.	3 (8)	1 (2)	0.216
pos.	pos.	pos.	pos.	pos.	31 (84)	12 (16)	0.096

*Mean number of “positive” cells per 2,000 cells. For the FBG, six circular ROIs including mesh fibers were analyzed per sample (n = 12). Comparison with t-test between 304,998 cells located in the FBG and 6,471,629 cells in the whole sample (= scar). Statistically significant differences are marked with asterisks*.

**Figure 7:**
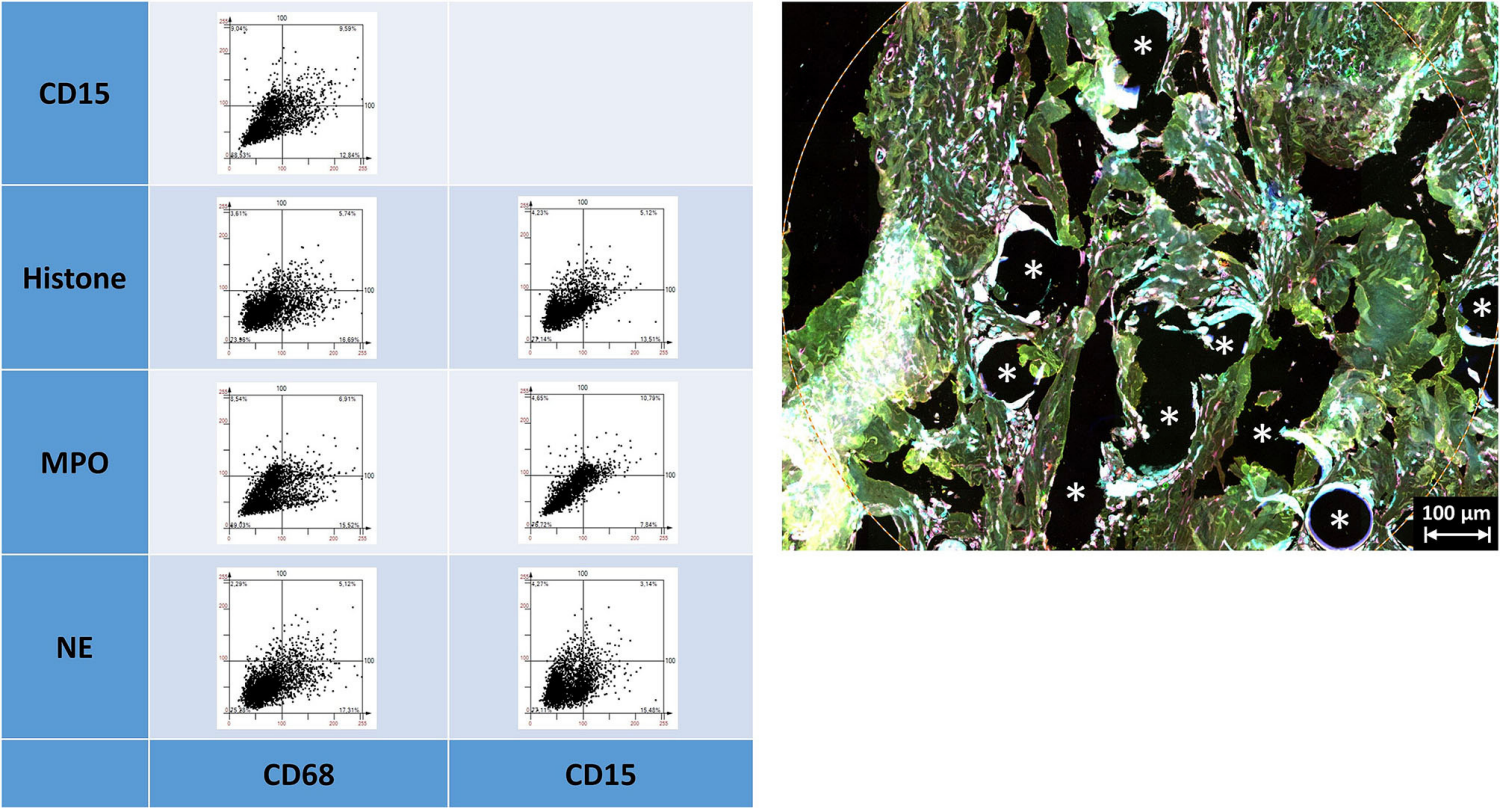
Examples of linear scatter plots from neutrophil panel. Scatter plots of mean cellular intensities of two markers in a 1 mm^2^ ROI placed around PP mesh fibers. The lines in the scatter plot mark the cut-off value 100. Cytometric analysis was performed with StrataQuest 7. Asterisks mark fiber locations.

The linearly scaled scatter plots usually revealed point clouds with a wide distribution of intensities rather than oval clouds with less variation in intensities (Figure 7). Many CD68+ cells showed coexpression of CD15, histone, MPO, and NE, and CD15+ cells were usually positive for histone, MPO, and NE, as expected.

**Figure 8:**
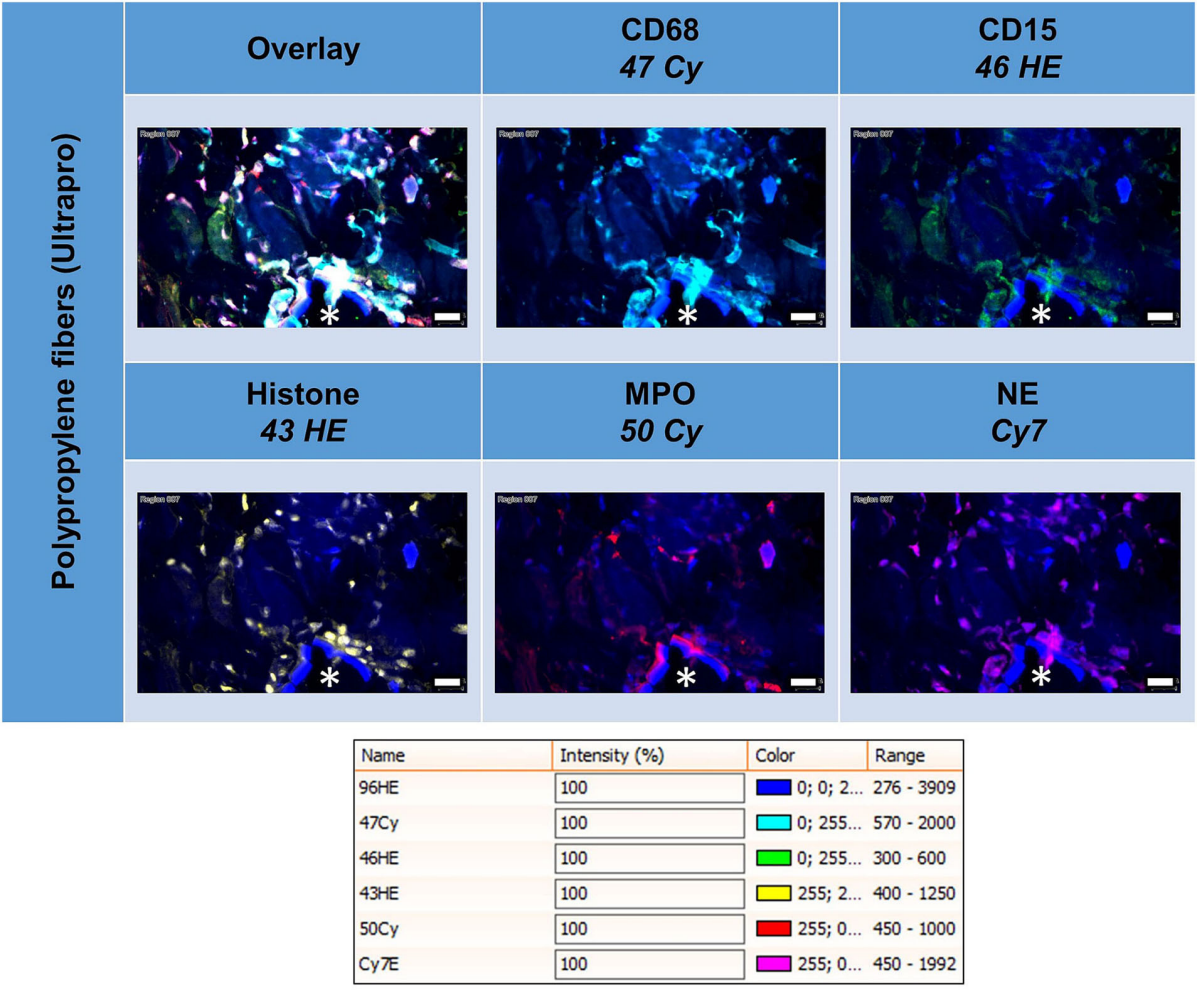
Tissue section of infected composite PP mesh labeled for nuclei with DAPI (blue), macrophages with CD68 (turquoise), neutrophils with CD15 (green), histone (yellow), myeloperoxidase (MPO, red), and neutrophil elastase (NE, magenta). EDAs indicate the presence of neutrophil extracellular traps (NETs). Asterisk marks a fiber location, scale bars = 50 μm.

Noteworthy, considerable extranuclear DAPI areas (EDA) were visible near the meshes (Figure 8). As DAPI is considered to bind specifically to DNA, these DAPI deposits can indicate the presence of neutrophil extracellular traps (NETs). Lowering the ranges for DAPI and excluding the area of the nuclear masks, EDAs could be identified, separated from the nuclear area, and the expression of the marker analyzed in the EDAs. Though the clinical relevance of neutrophils and NETs still is obscure, this study demonstrated their presence within the inflammatory infiltrate of the FBG and may be considered as the reason for long-term immunological problems of some patients (7).

### Collaborative Network of Macrophages, Lymphocytes, and Neutrophils Within the FBG

**Figure 9:**
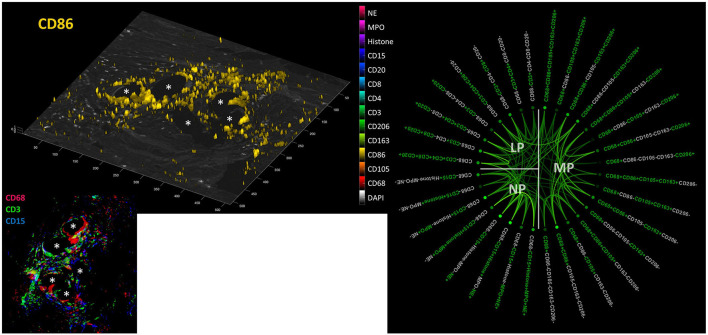
Collaboration network in the FBG. **Left***:* Example image from a video file (available as Supplementary Material) showing all “positive” cells (threshold = mean + 2 SD) for all 13 markers on a DAPI image; CD86 is shown here. **Inset***:* CD68+ macrophages (red), CD3+ lymphocytes (green), and CD15+ neutrophils (blue) in the FBG of a PP mesh. Asterisks mark fiber locations. **Right***:* Significant Pearson's correlations (*p* < 0.05) within and between macrophage (MP), lymphocyte (LP), and neutrophile (NP) panels. Marker colored green = positive and marker colored light gray = negative. The stronger the saturation of the depicted correlations between individual marker combinations, the higher is their correlation coefficient.

Considering positive staining as intensities above mean + 2 SD, there were abundant CD68+ macrophages, CD3+ lymphocytes, and CD15+ neutrophils seen within the inflammatory infiltrate around PP fibers (Figure 9). Visualization revealed the spatial expression of the 13 markers used, and some of them coexpressed in similar cell clusters. Of the 4,560 possible correlations among the different panels of “positive” or “negative” markers, there were 898 significant Pearson's two-sided correlations (*p* < 0.05) reflecting the many functional linkages among the various markers. Since most markers showed, at least in some cells, that their expression occurred independently of the expression of other markers, higher correlations with *r* > 0.6 were rare (*n* = 81).

## Discussion

In comparison with previous studies with just two markers besides DAPI, the use of 5-marker multiplex immunofluorescence microscopy demonstrated the marked complexity of the biology within a FBG. The distinct spatial distribution of the markers within the entire tissue sample confirmed the high specificity of the antibody–protein binding. However, the complex and overlapping expression signature demonstrated that there was hardly any cell pattern that uniformly determined the presence of a specific subgroup or cell cluster with either identical origin or similar functionality.

The separation of M1 and M2 macrophages due to their mere staining with CD86 or CD163/CD206 appears to be incomplete to mirror the high heterogeneity of the macrophage response to the meshes. All the more so as the lymphocytic system obviously contributes to a similar extent and complexity. Additionally, not least, the detection of EDAs around the mesh fibers with abundant expression of histone, MPO, and NE underlines the importance of the neutrophils and their formation of neutrophil extracellular traps, which may be responsible for the ongoing chronic inflammation and any possible autoimmune stress.

However, despite the high evidence for the present high level of coexpression, any attempt for quantification of immunohistochemistry has severe limitations:

The possible impact on functionality by the expression of proteins with variable intensities, which may be the consequence of different concentrations of the binding epitopes on the cells, is known, but hard to control (8). The poor quality of the tissue with, for example, long ischemia before fixation may reduce the binding sites further. Patient's biology and their immunological response may differ to an unknown extent. The surgical trauma and wound infection may change the local tissue response to the mesh materials.

Quantification of multiplex staining results, of course, has lots of confounders, such as overlaying background signals, unspecific binding of the primary or secondary antibody, distinct affinity binding of the dye, variable number of binding sites in heterogeneous cells, and alteration of epitopes by ischemia or the fixation procedure. Some of them can be excluded by reasonable spatial staining pattern and using various controls. However, for the question at what staining intensity a cell should be considered “positive” there still is not an easy answer. Sometimes, the staining signal is relatively weak but distributed over most of the cell, whereas in other cases, a hyperintense signal emanates from only part of the cell, resulting in the same mean signal. Without additional analysis, it is impossible to define any as functional “negative” or “positive.” Adjusting the intensity ranges using imaging tools can decrease or increase the image contrast, but it does not solve the problem. We decided to use the automatic ranges provided by TissueFAXS for the StrataQuest analyses and to use a fixed cut-off value of 100 to determine “positive” cells.

Any quantification needs to define a reliable cut-off to determine at what intensity a cell may be considered “positive” (9). The cut-off usually does not result from natural order with distinct intensities but always has to be fixed manually and arbitrarily (10). The intensity may be measured as mean or maximum in an eroded or dilated nuclear mask. The definition of reliable ROIs is essential to overcome the usual huge heterogeneity of the tissue samples, in particular, if the mesh fibers are removed during the cutting process. Using specific LED light sources in combination with bandpass filters ideally matched to their spectra and selected fluorescent dyes, any artificial overlap by interfering signals must be avoided. A mean intensity > 100 does not automatically reflect a “positive” cell for a maker. It has to be checked by visual control and backward gating whether the staining pattern is reasonable and in accordance with the published literature. Conversely, a mean intensity <100 does not prove that the cell does not express the marker anyhow. However, a fixed cut-off value of 100 reduces the subjective impact of a manual gating and improves reproducibility.

This “high” cut-off value may exclude several cells that are only partially or overall “weakly” stained, but a lower cut-off would result in higher percentages without contradicting the fact that there are many coexpressions and complex marker patterns in cells of a FBG.

Despite the many limitations mentioned above in quantifying cells with “positive” staining, the present protocol provides tools to analyze the inflammatory response to meshes in a highly standardized, reproducible, reliable, and objective manner.

In regard to the many confounders with an impact on the cell response to meshes and in consideration of the limited information given by clinicians, this study cannot link its quantification of inflammatory cells with a specific clinical outcome. However, this protocol and the results for this mix of various materials may serve as a standard for future comparisons to detect any gross violation from the standard and to identify high-risk materials.

## Conclusion

The cells of the inflammatory infiltrate around mesh fibers showed a variable signature of macrophage, lymphocyte, and neutrophil markers in multiplex immunofluorescence microscopy. Instead of distinct subgroups of cells with clear marker profiles, we found comprehensive interference resulting in a blurred cloud of overlapping coexpression. A clear physiological threshold to separate “negative” and “positive” cells was not seen for any of the 13 markers, which, in contrast, all showed a continuous spectrum of intensities. In addition to the polarization filter in bright field microscopy, viewing polymer fibers with bandpass filters can help to analyze meshes and their degradation in tissue specimens.

The recent decades of research in the field of biomaterials and meshes, respectively, have been characterized by semi-quantitative analyses mainly focusing on macrophages, defined by immunohistochemistry usually with CD68 as the only marker. These results have been used to assess the biocompatibility and potential risks of the implants. However, these approaches obviously were not able to reflect the complexity of the foreign body reaction around the material and to provide data for reliable comparisons. In this study, we demonstrate that single marker approaches are not suitable to define specific subgroups of immunological cells in the FBG, which is possible with multiplex methods. Furthermore, by focusing on the mean intensities within the nuclear area, a precise quantification is feasible. The present data of a variety of different meshes thus provide a first standard for future comparisons. It still has to be shown by additional methods and/or targeted material modifications, which of these marker profiles are relevant for the clinical outcome. This study clearly demonstrates that future analyses should be done with multiplex imaging to enable precision medicine.

## Data Availability Statement

The raw data supporting the conclusions of this article will be made available by the authors, without undue reservation.

## Ethics Statement

The studies involving human participants were reviewed and approved by Ethic Committee of the University Hospital of the RWTH Aachen EK 239/19. The patients/participants provided their written informed consent to participate in this study.

## Author Contributions

UK performed the microscopic measurements. AD, JS, and UK together prepared the analysis and the manuscript. All authors contributed to the article and approved the submitted version.

## Funding

The support by the Federal Ministry of Education and Research (FKZ 13GW0108B) enabled the acquisition of the Tissue Gnostics system.

## Conflict of Interest

The authors declare that the research was conducted in the absence of any commercial or financial relationships that could be construed as a potential conflict of interest.

## Publisher's Note

All claims expressed in this article are solely those of the authors and do not necessarily represent those of their affiliated organizations, or those of the publisher, the editors and the reviewers. Any product that may be evaluated in this article, or claim that may be made by its manufacturer, is not guaranteed or endorsed by the publisher.
