# Supplementary material for: Quantitative Characterization of Macrophage, Lymphocyte, and Neutrophil Subtypes Within the Foreign Body Granuloma of Human Mesh Explants by 5-Marker Multiplex Fluorescence Microscopy
Source: Front Med (Lausanne). 2022 Feb 15;9:777439. doi: 10.3389/fmed.2022.777439 (PMC8887619; doi:10.3389/fmed.2022.777439)
Supplement: Supplementary file 1 [file Data_Sheet_1.zip › Supplementary Material 3.pdf]

Supplement 3

Content

Controls

- Figure 1: Cumulative distribution (1a) and distribution (1b) of mean cellular intensities for CD68 labeled with OPAL™ 480 and controls (p. 2-3)
- Figure 2: Cumulative distribution (2a) and distribution (2b) of mean cellular intensities for CD86, CD3, and CD15 labeled with OPAL™ 520 and controls (p. 4-5)
- Figure 3: Cumulative distribution (3a) and distribution (3b) of mean cellular intensities for CD163 and CD8 labeled with OPAL™ 650 and controls (p. 6-7)

High resolution images

- Figure 4-9: Grayscale images (single channel) from the lymphocyte panel (p. 8-13)
- Figure 10-23: Color images (3 channels) from the lymphocyte panel (p. 14-27)
- Figure 24: Color image of T cells and nuclei (4 channels) from lymphocyte panel (p. 28)
- Figure 25: Color image of macrophages, T cells, B cells and nuclei (6 channels) from lymphocyte panel (p. 29)

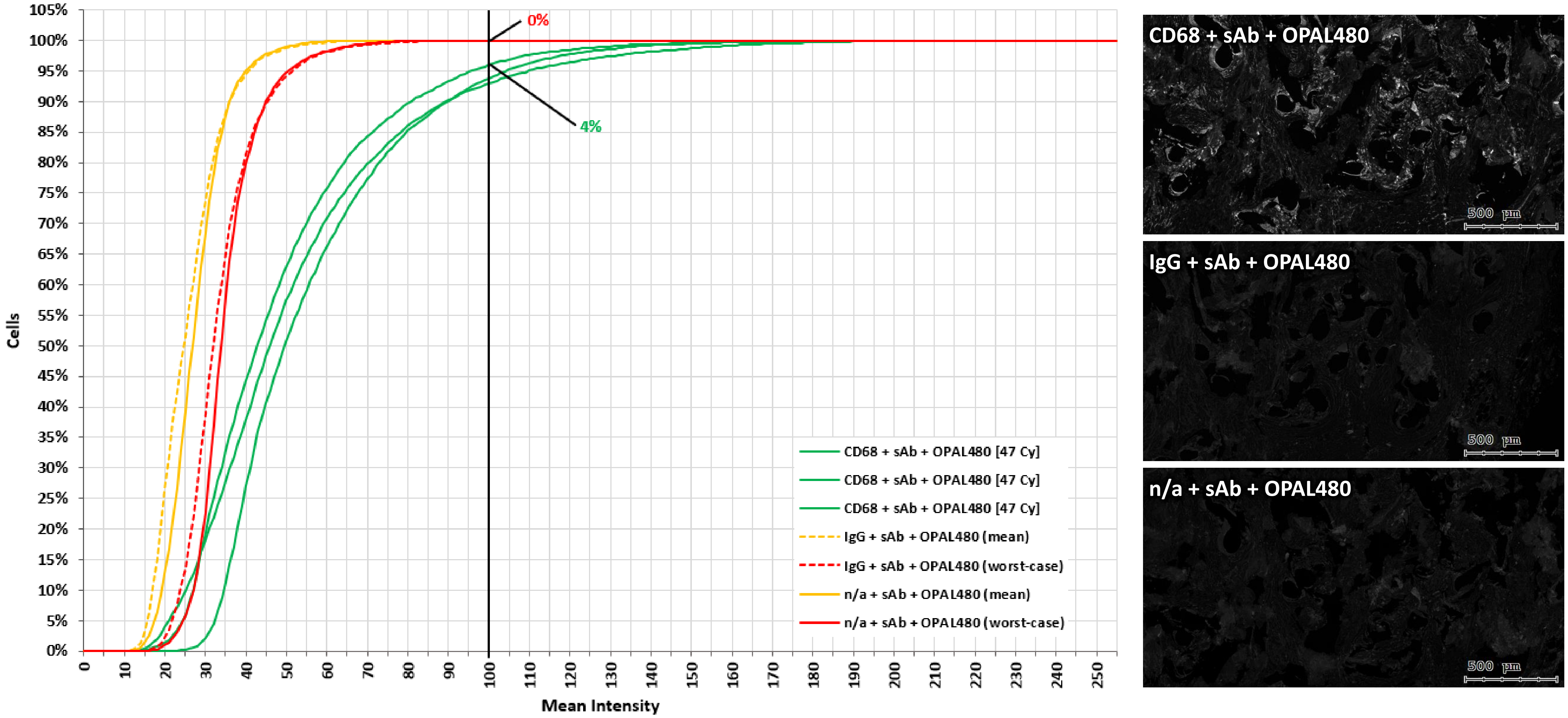

Figure 1a: *Left:* Cumulative distribution of mean cellular intensities for CD68 labeled with OPAL™ 480 (3x, one for each panel), isotype control (IgG) with the mean and lowest (= worst-case) of the maximum ranges of the panels (macrophage, lymphocyte, neutrophil), and no primary antibody control, i.e., application of only the second antibody (sAb) and the fluorophore. *Right:* Example images from a mesh sample – top: complete labeling with CD68, middle: isotype control, bottom: no primary antibody.

The curves of the controls are left-shifted and there is no indication of a distinct cut-off; the applied cut-off 100 reliably detects "positive" cells without including many "false positives".

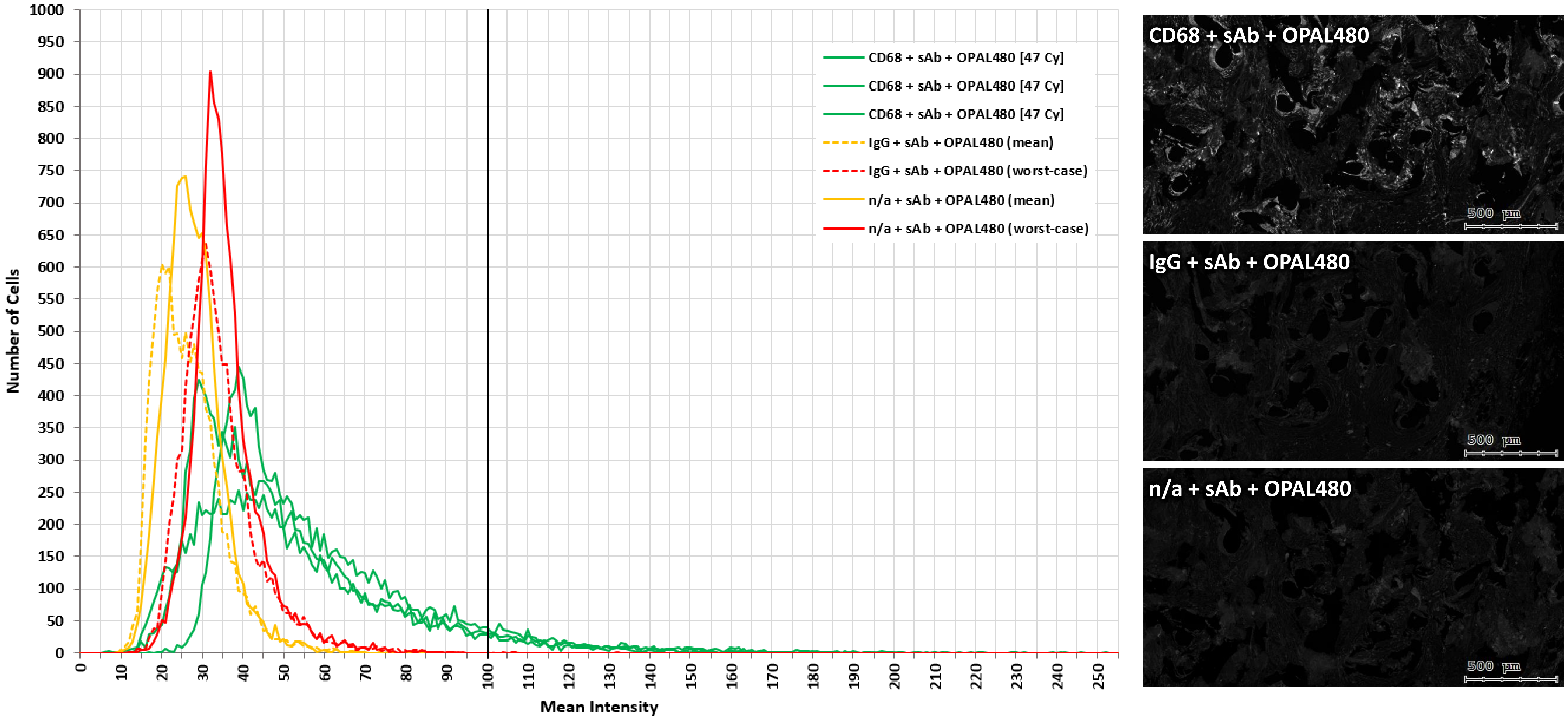

Figure 1b: ***Left:*** **Distribution of mean cellular intensity for CD68 labeled with OPAL^{TM} 480 (3x, one for each panel), isotype control (IgG) with the mean and lowest (= worst-case) of the maximum ranges of the panels (macrophage, lymphocyte, neutrophil), and no primary antibody, i.e., application of only the second antibody (sAb) and the fluorophore.** ***Right:*** **Example images from a mesh sample – top: complete labeling with CD68, middle: isotype control, bottom: no primary antibody.**

The curves of the controls are left-shifted and there is no indication of a distinct cut-off; the applied cut-off 100 reliably detects "positive" cells without including many "false positives".

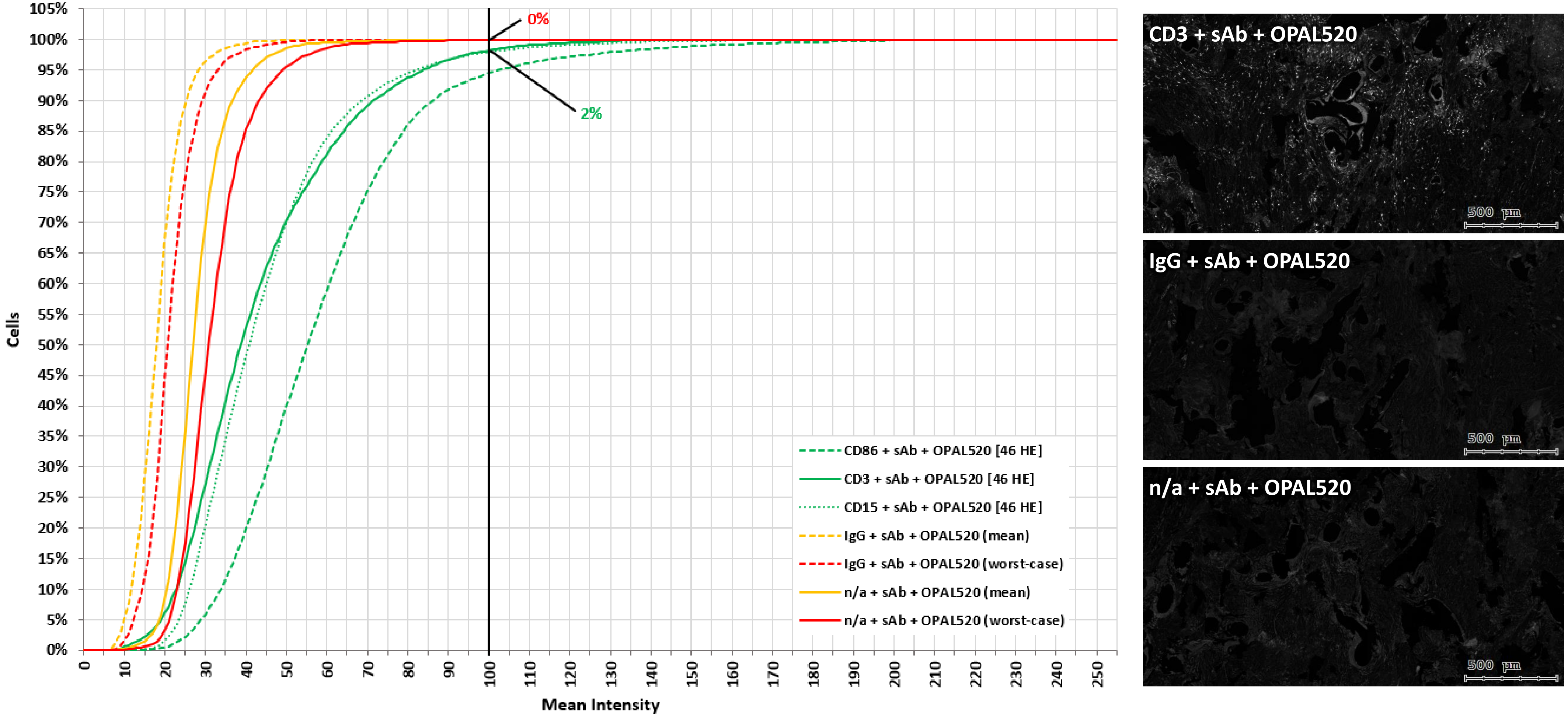

Figure 2a: ***Left:*** **Cumulative distribution of mean cellular intensities for CD86, CD3, and CD15 labeled with OPAL^{TM} 520, isotype control (IgG) with the mean and lowest (= worst-case) of the maximum ranges of the panels (macrophage, lymphocyte, neutrophil), and no primary antibody, i.e., application of only the second antibody (sAb) and the fluorophore.** ***Right:*** **Example images from a mesh sample – top: complete labeling with CD3, middle: isotype control, bottom: no primary antibody.**

The curves of the controls are left-shifted and there is no indication of a distinct cut-off; the applied cut-off 100 reliably detects "positive" cells without including many "false positives".

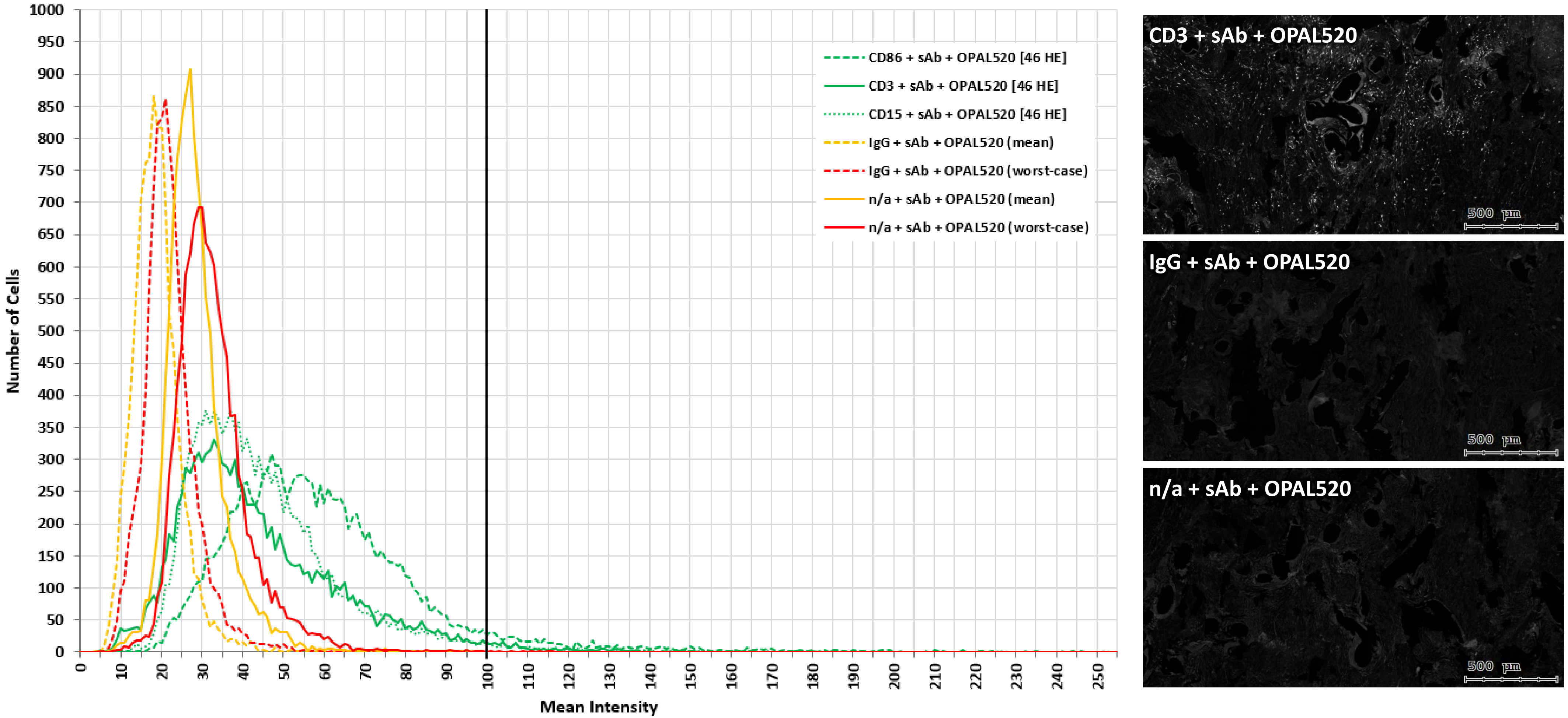

Figure 2b: *Left:* Distribution of mean cellular intensity for CD86, CD3, and CD15 labeled with OPAL^{TM} 520, isotype control (IgG) with the mean and lowest (= worst-case) of the maximum ranges of the panels (macrophage, lymphocyte, neutrophil), and no primary antibody, i.e., application of only the second antibody (sAb) and the fluorophore. *Right:* Example images from a mesh sample – top: complete labeling with CD3, middle: isotype control, bottom: no primary antibody.

The curves of the controls are left-shifted and there is no indication of a distinct cut-off; the applied cut-off 100 reliably detects “positive” cells without including many “false positives”.

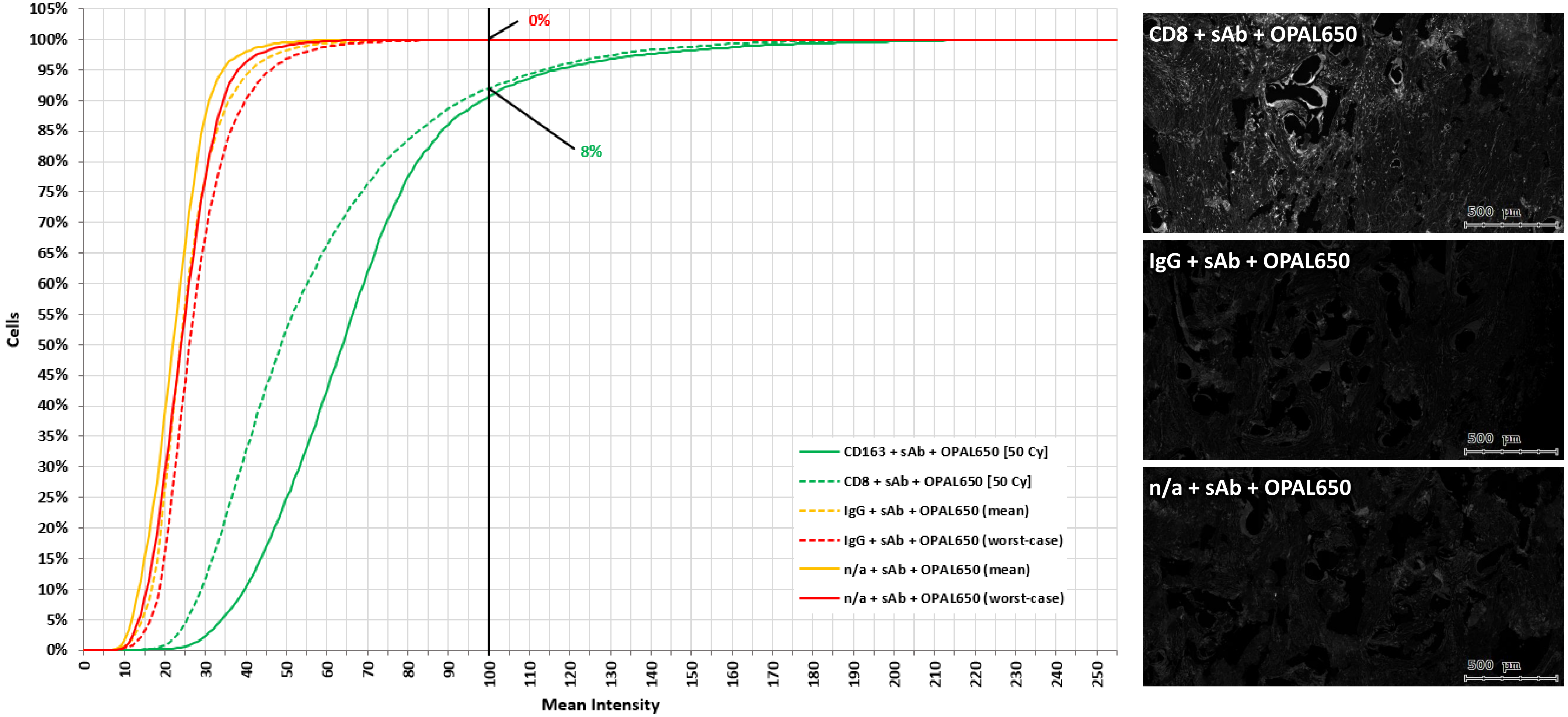

Figure 3a: ***Left:*** **Cumulative distribution of mean cellular intensities for CD163 and CD8 labeled with OPAL^{TM} 650, isotype control (IgG) with the mean and lowest (= worst-case) of the maximum ranges of the panels (macrophage, lymphocyte, neutrophil), and no primary antibody, i.e., application of only the second antibody (sAb) and the fluorophore.** ***Right:*** **Example images from a mesh sample – top: complete labeling with CD8, middle: isotype control, bottom: no primary antibody.**

The curves of the controls are left-shifted and there is no indication of a distinct cut-off; the applied cut-off 100 reliably detects “positive” cells without including many “false positives”.

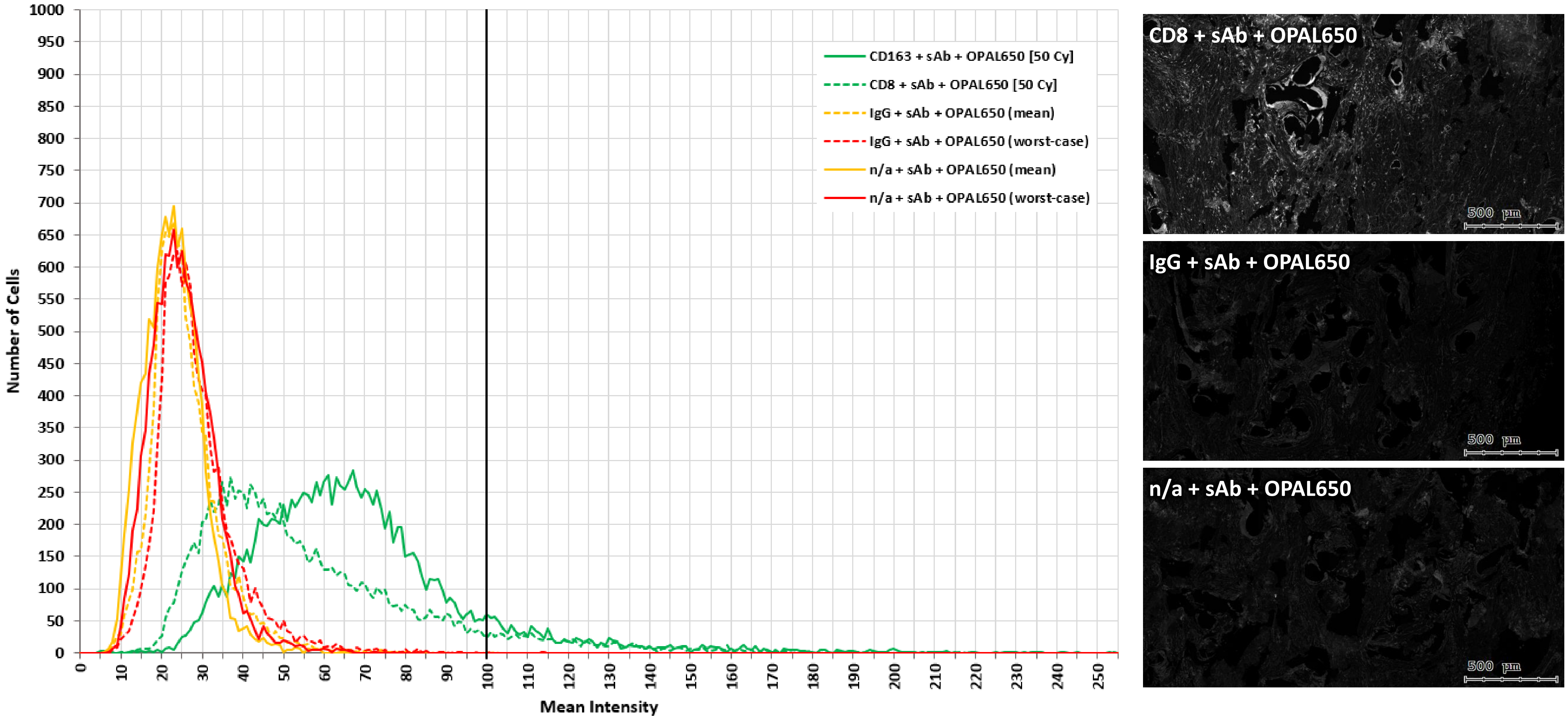

Figure 3b: ***Left:*** **Distribution of mean cellular intensity for CD163 and CD8 labeled with OPAL™ 650, isotype control (IgG) with the mean and lowest (= worst-case) of the maximum ranges of the panels (macrophage, lymphocyte, neutrophil), and no primary antibody, i.e., application of only the second antibody (sAb) and the fluorophore.** ***Right:*** **Example images from a mesh sample – top: complete labeling with CD8, middle: isotype control, bottom: no primary antibody.**
The curves of the controls are left-shifted and there is no indication of a distinct cut-off; the applied cut-off 100 reliably detects "positive" cells without including many "false positives".

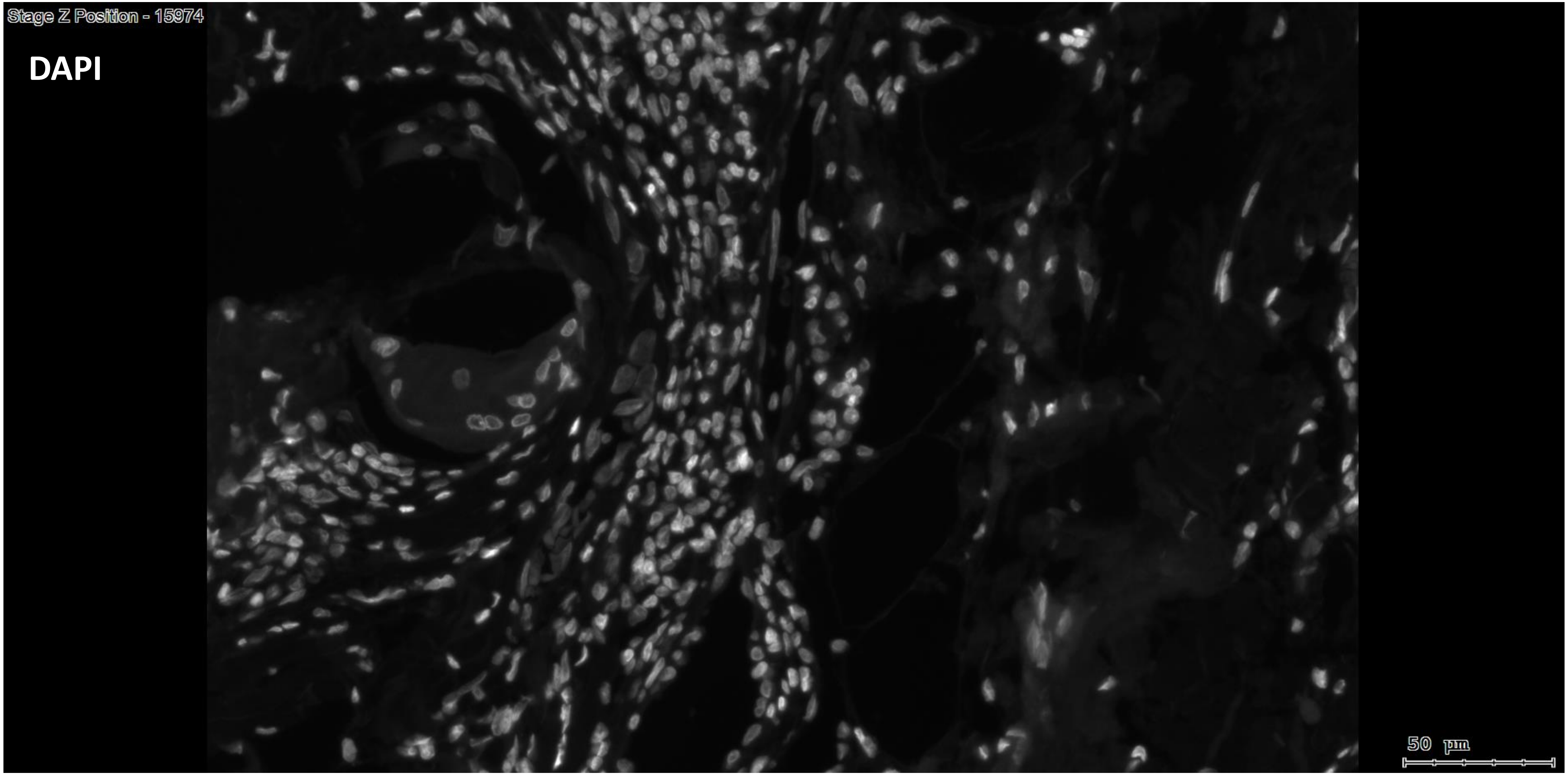

Figure 4: Gray scale image of cell nucleus labeling with DAPI

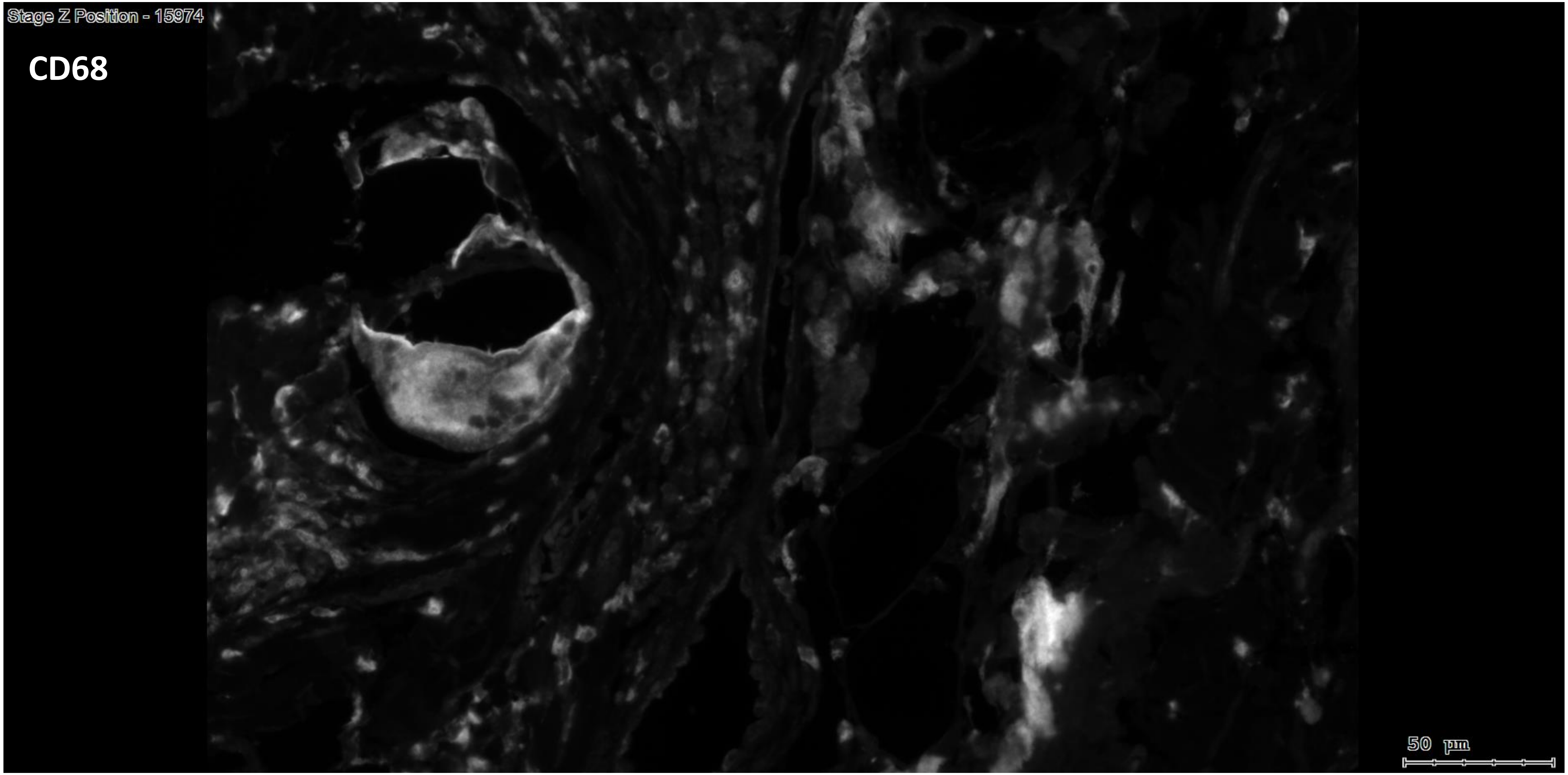

Figure 5: Gray scale image of macrophage labeling with CD68

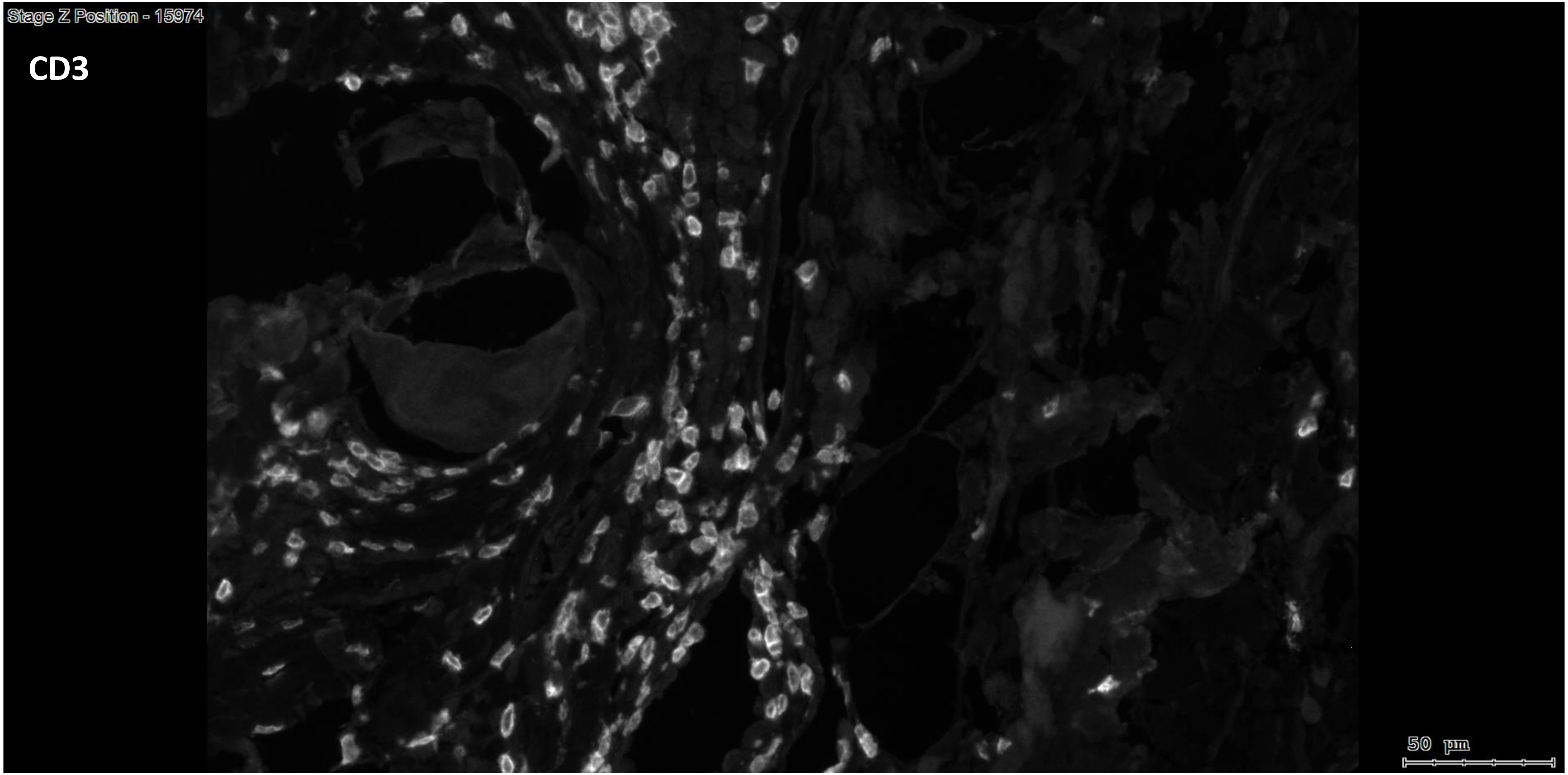

Figure 6: Gray scale image of labeling of T cells with CD3

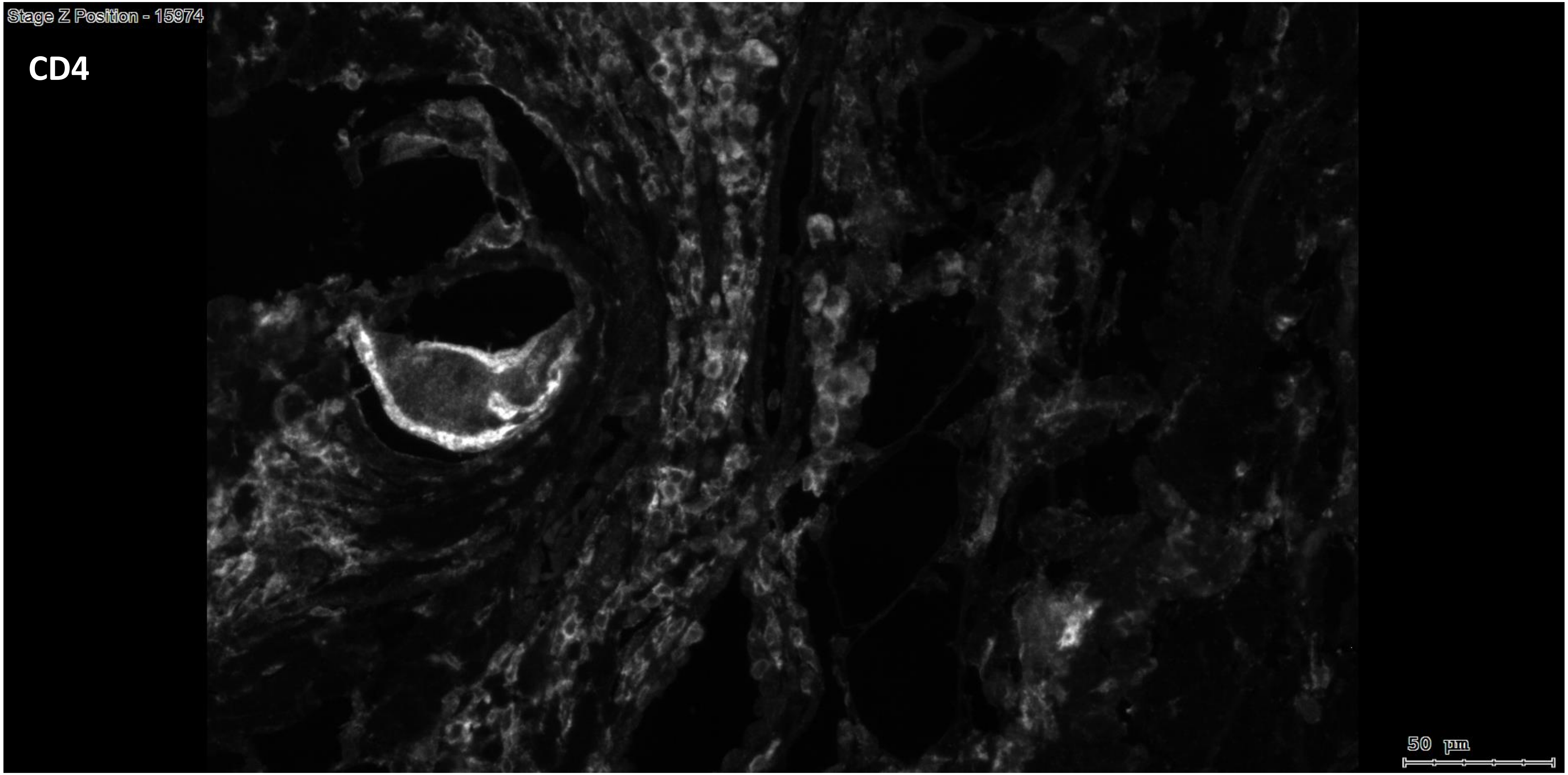

Figure 7: Gray scale image of T-helper cell labeling with CD4

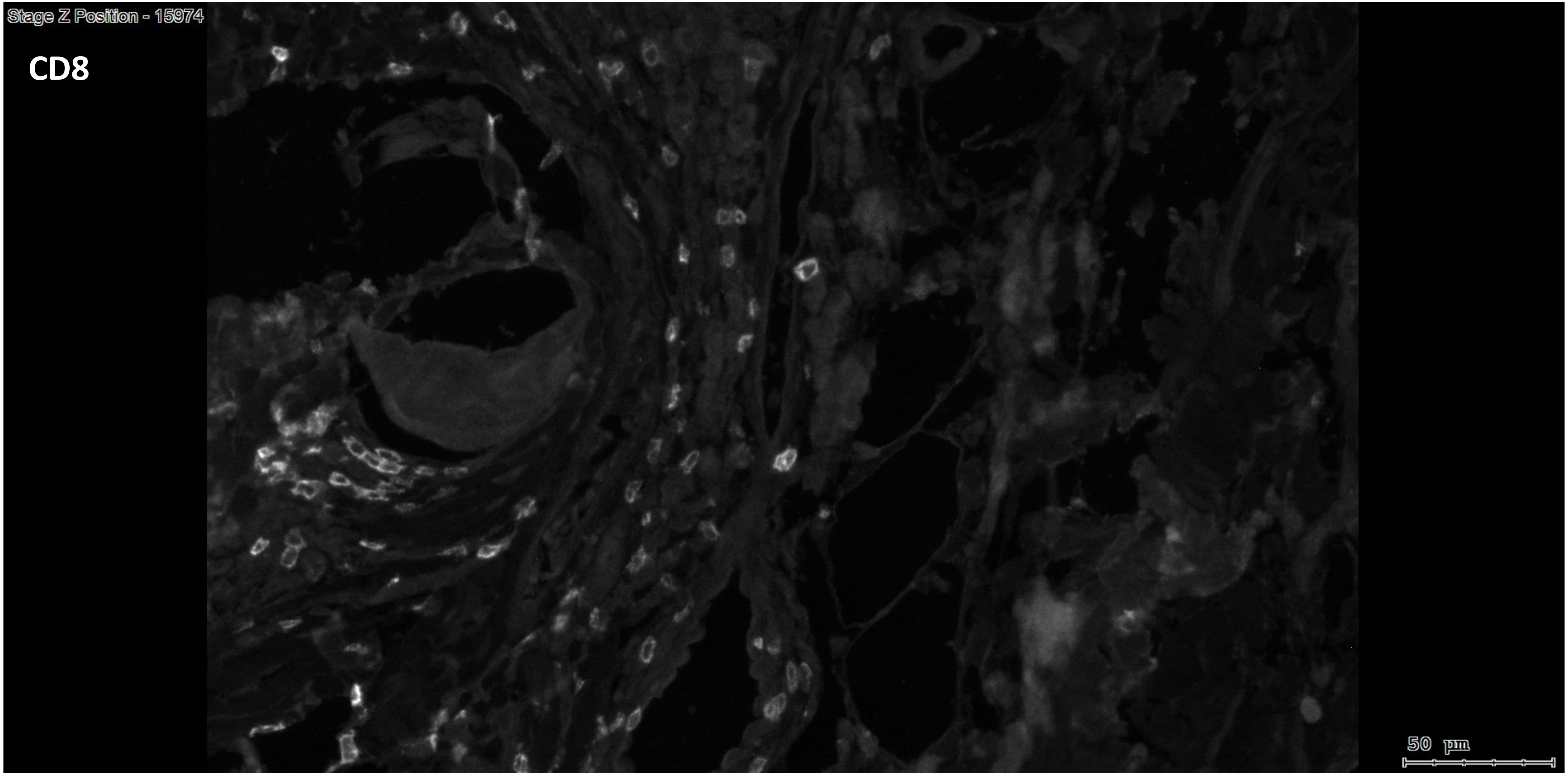

Figure 8: Gray scale image of labeling cytotoxic T-cell with CD8

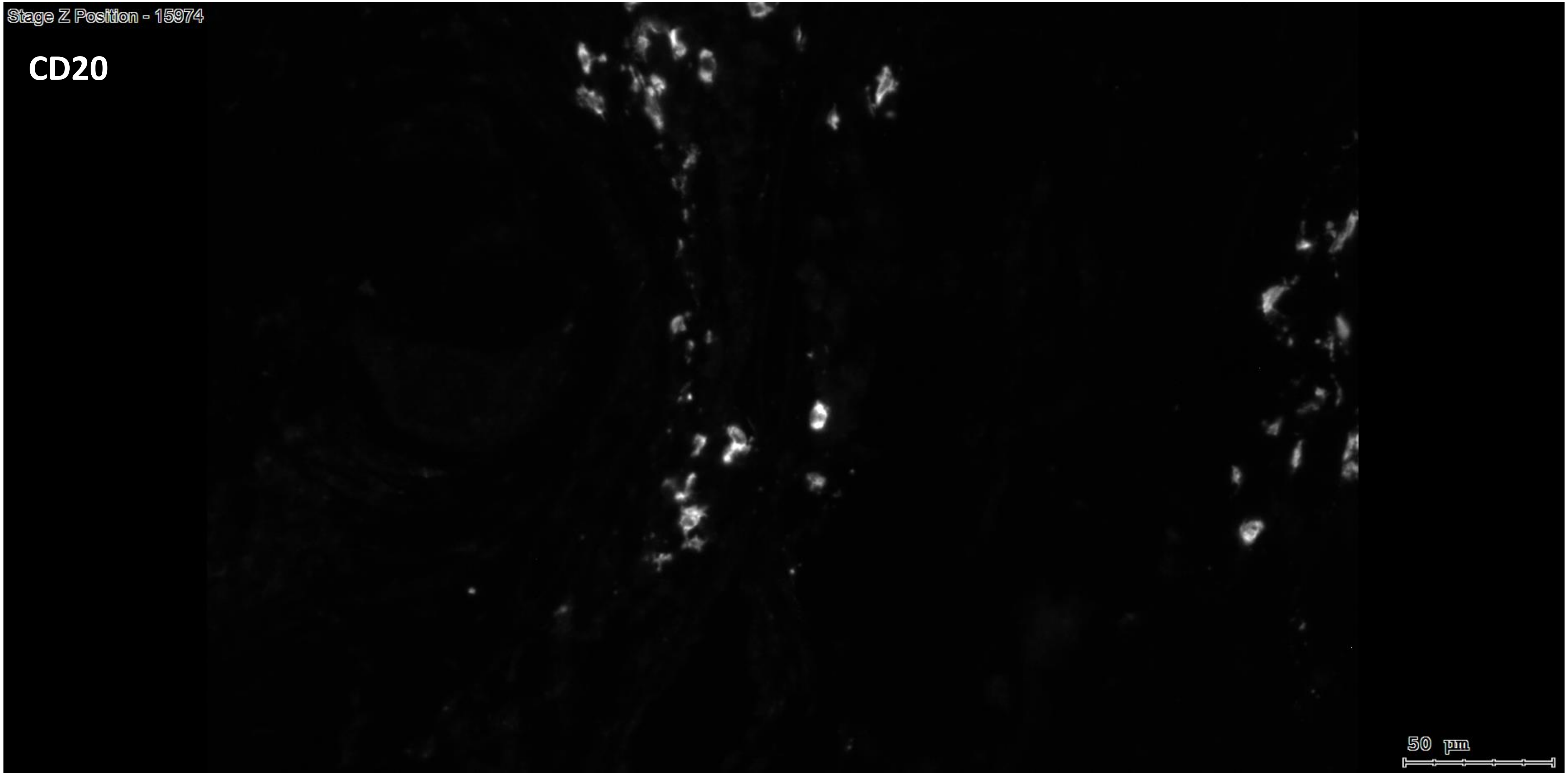

Figure 9: Gray scale image of labeling B cells with CD20

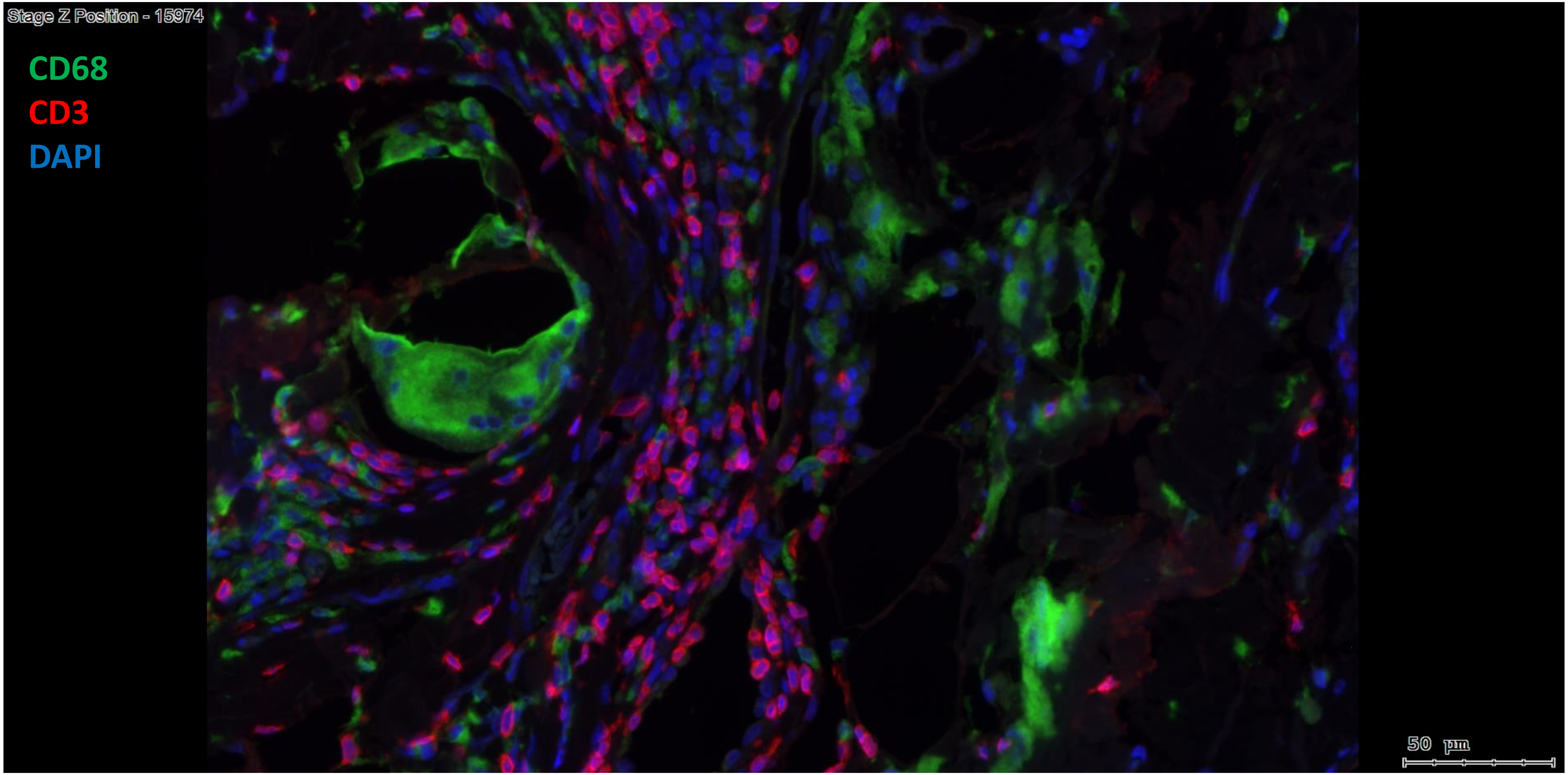

Figure 10: Macrophage labeling with CD68 (green), labeling of T cells with CD3 (red) and nuclei with DAPI (blue); adapted image to primary colors, i.e., colors are separated, but in case of co-expression, mixed colors, such as yellow/orange, turquoise or magenta/purple, result

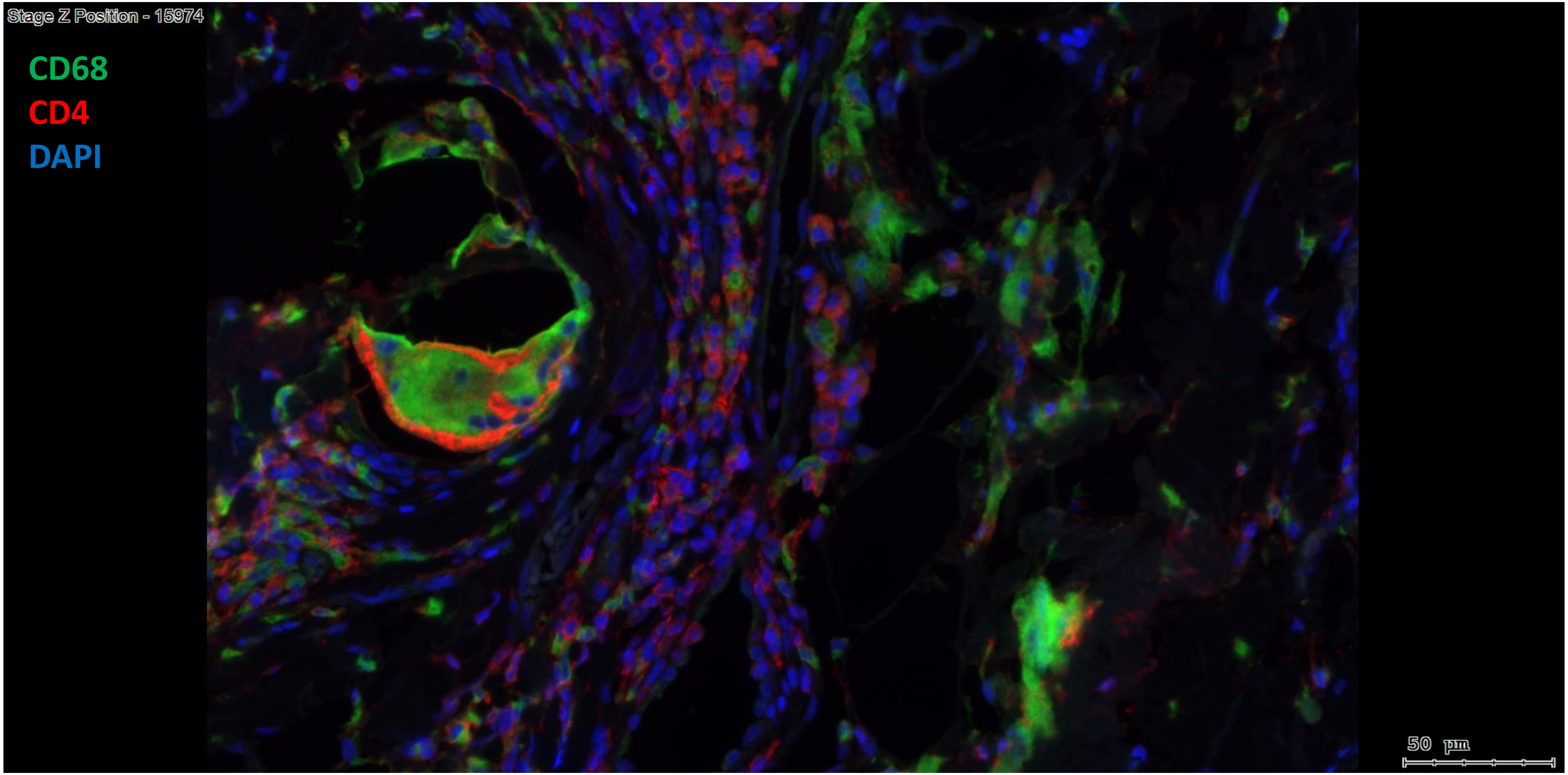

Figure 11: Macrophage labeling with CD68 (green), labeling of T-helper cells with CD4 (red) and nuclei with DAPI (blue); adapted image to primary colors

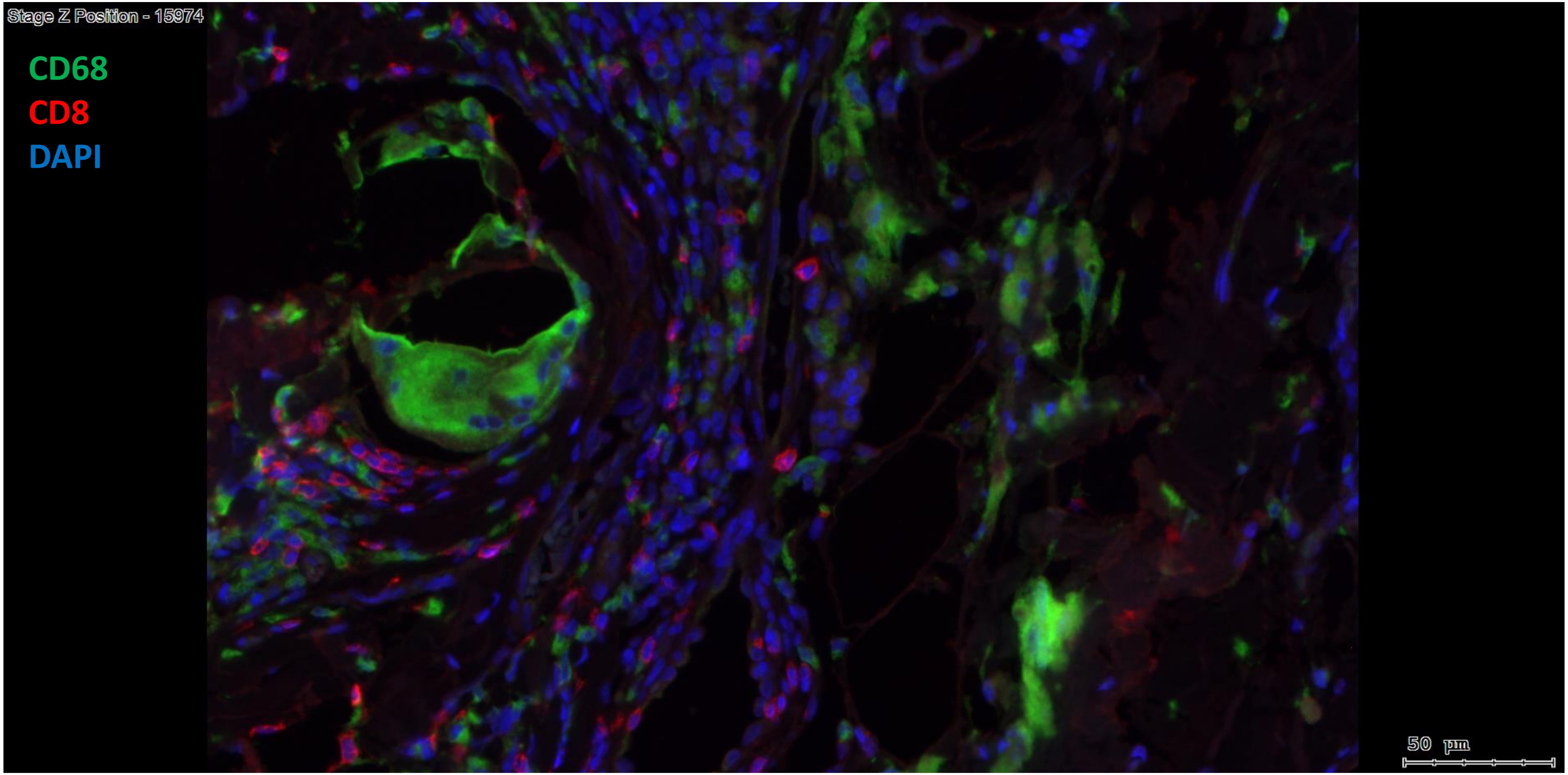

Figure 12: Macrophage labeling with CD68 (green), labeling of cytotoxic T-cells with CD8 (red) and nuclei with DAPI (blue); adapted image to primary colors

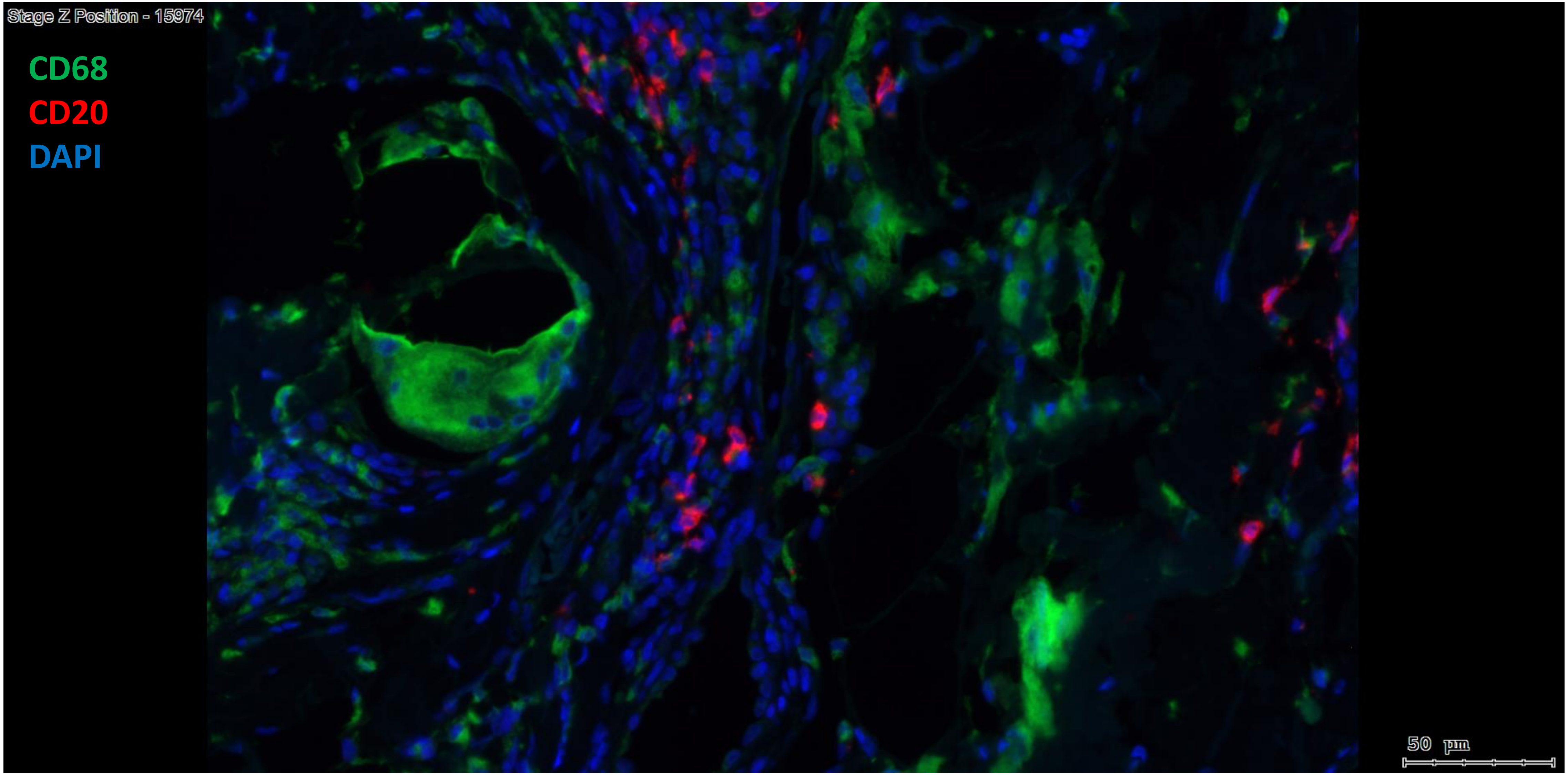

Figure 13: Macrophage labeling with CD68 (green), labeling of B cells with CD20 (red) and nuclei with DAPI (blue); adapted image to primary colors

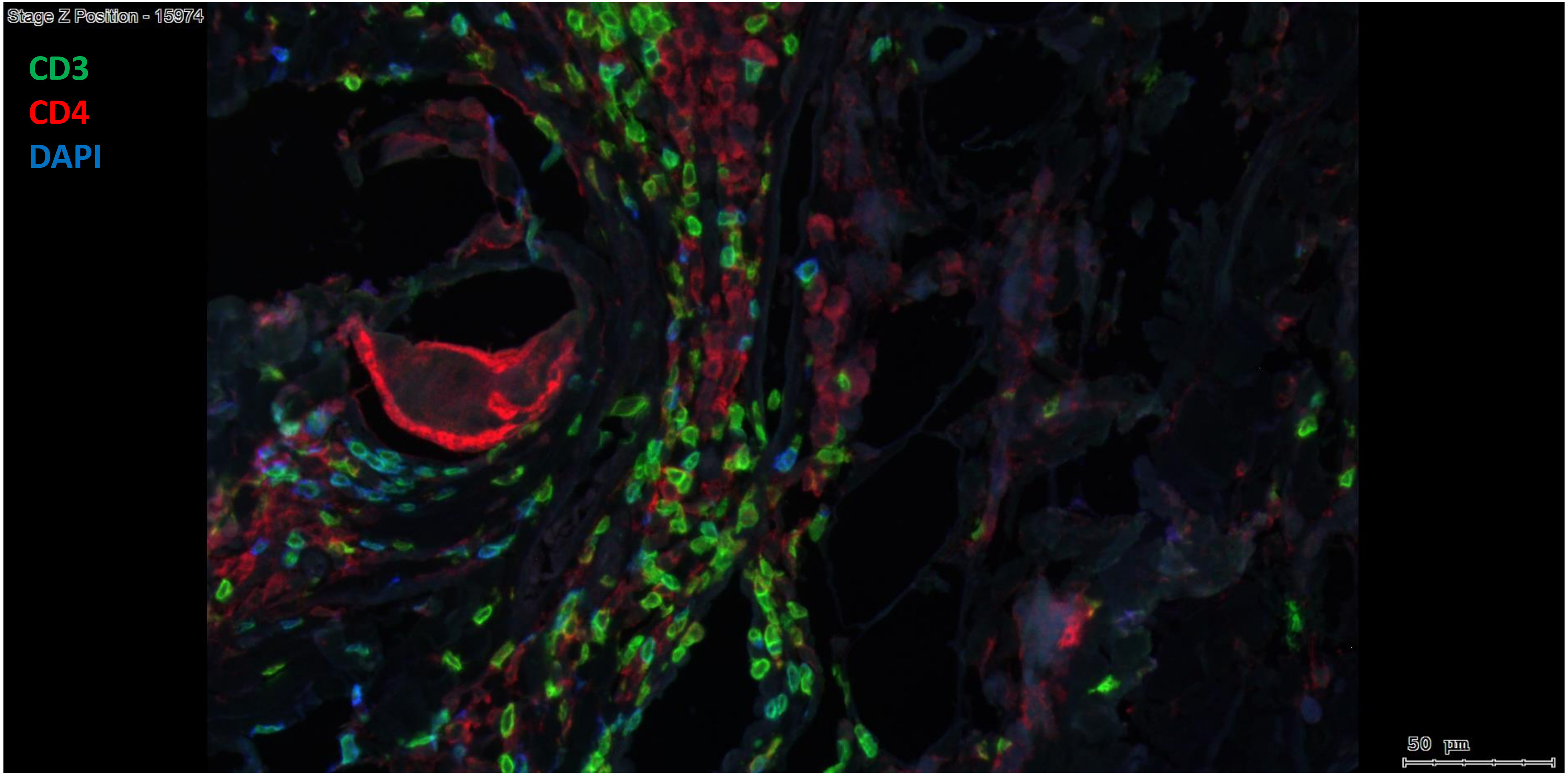

Figure 14: T-cell labeling with CD3 (green), labeling of T-helper cells with CD4 (red) and nuclei with DAPI (blue); adapted image to primary colors

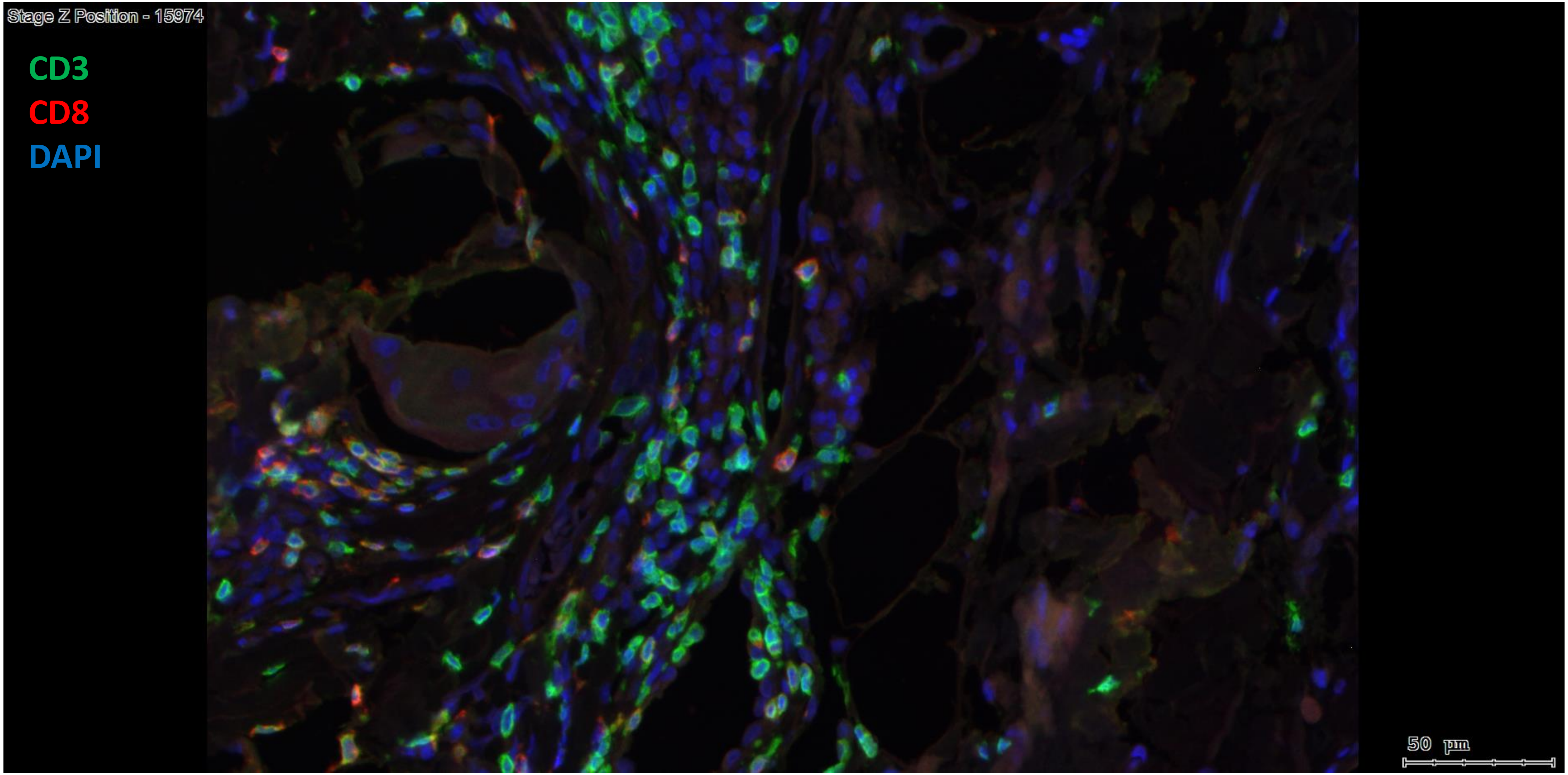

Figure 15: T-cell labeling with CD3 (green), labeling of cytotoxic T-cells with CD8 (red) and nuclei with DAPI (blue); adapted image to primary colors

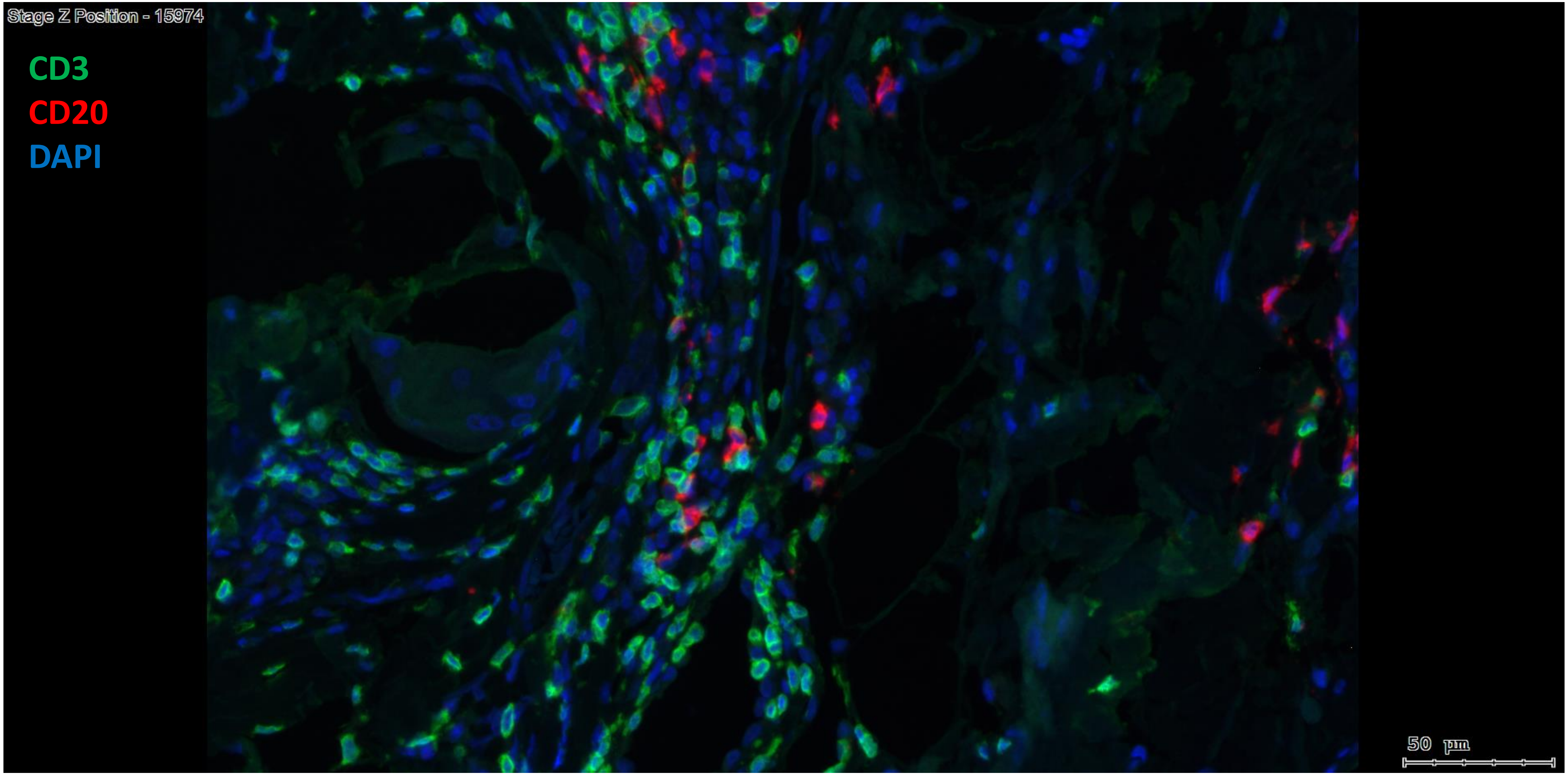

Figure 16: T-cell labeling with CD3 (green), labeling of B cells with CD20 (red) and nuclei with DAPI (blue); adapted image to primary colors

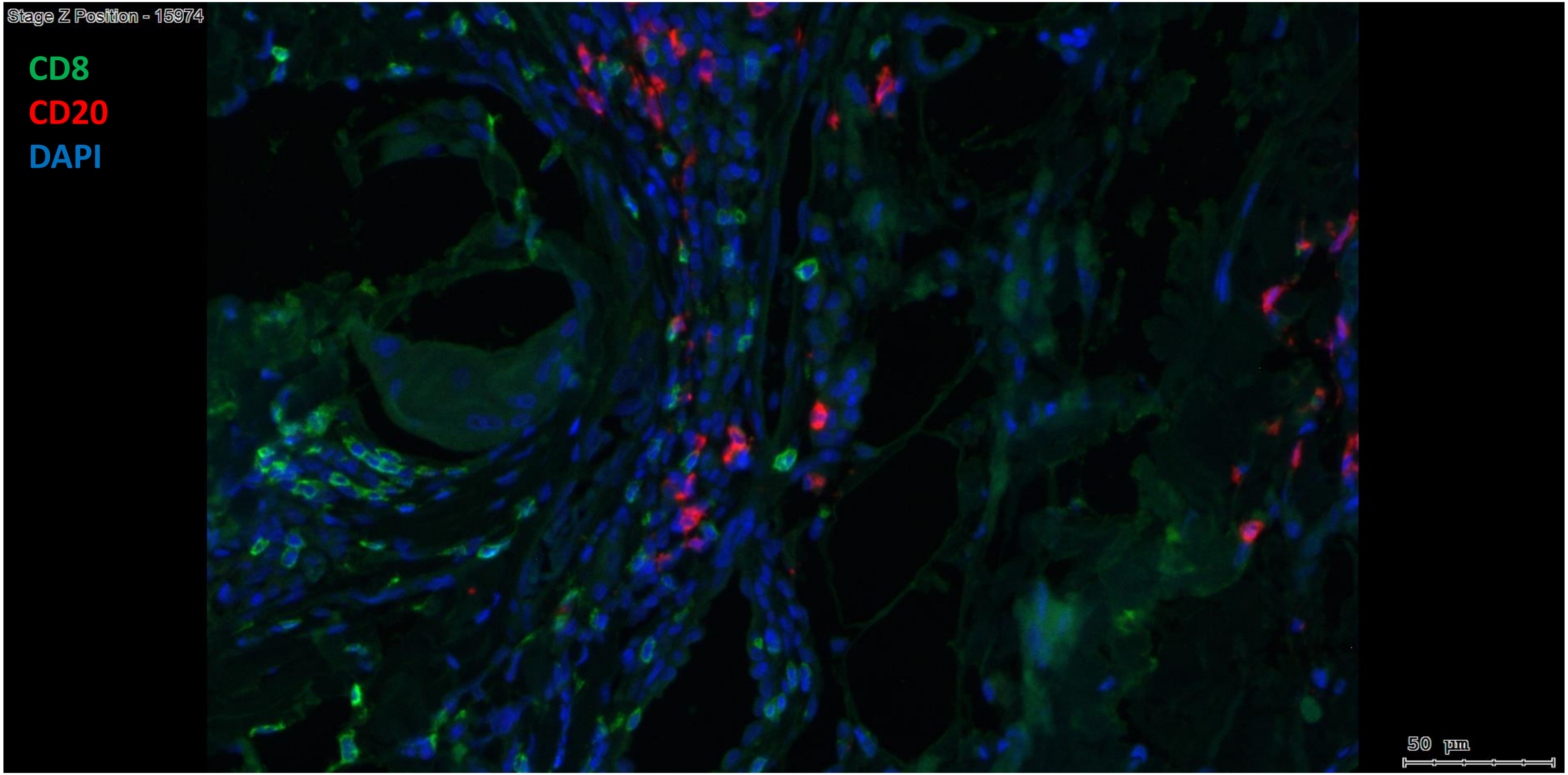

Figure 17: cytotoxic T-cell labeling with CD8 (green), labeling of B cells with CD20 (red) and nuclei with DAPI (blue); adapted image to primary colors

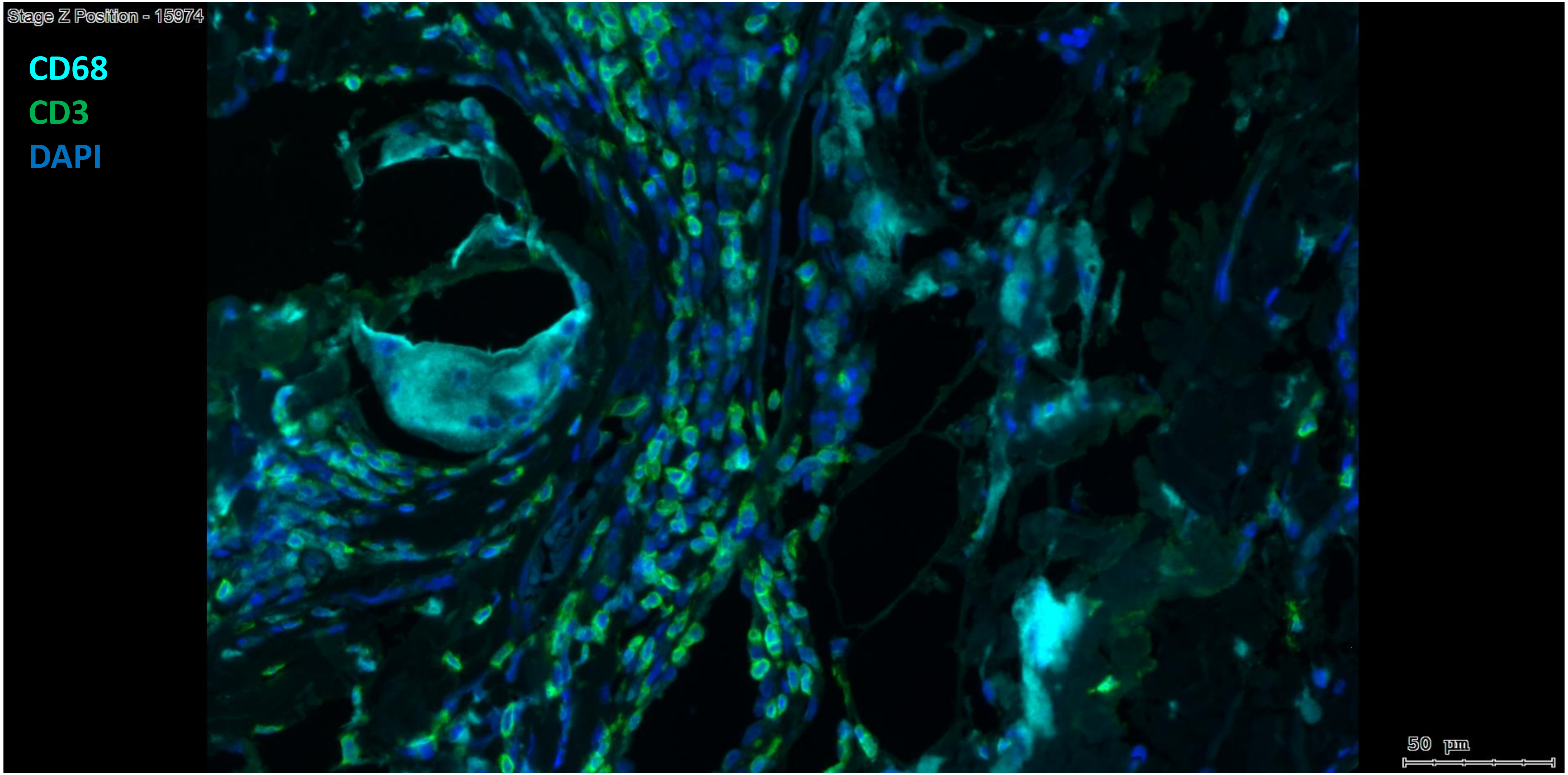

Figure 18: Macrophage labeling with CD68 (turquoise), labeling of T cells with CD3 (green) and nuclei with DAPI (blue)

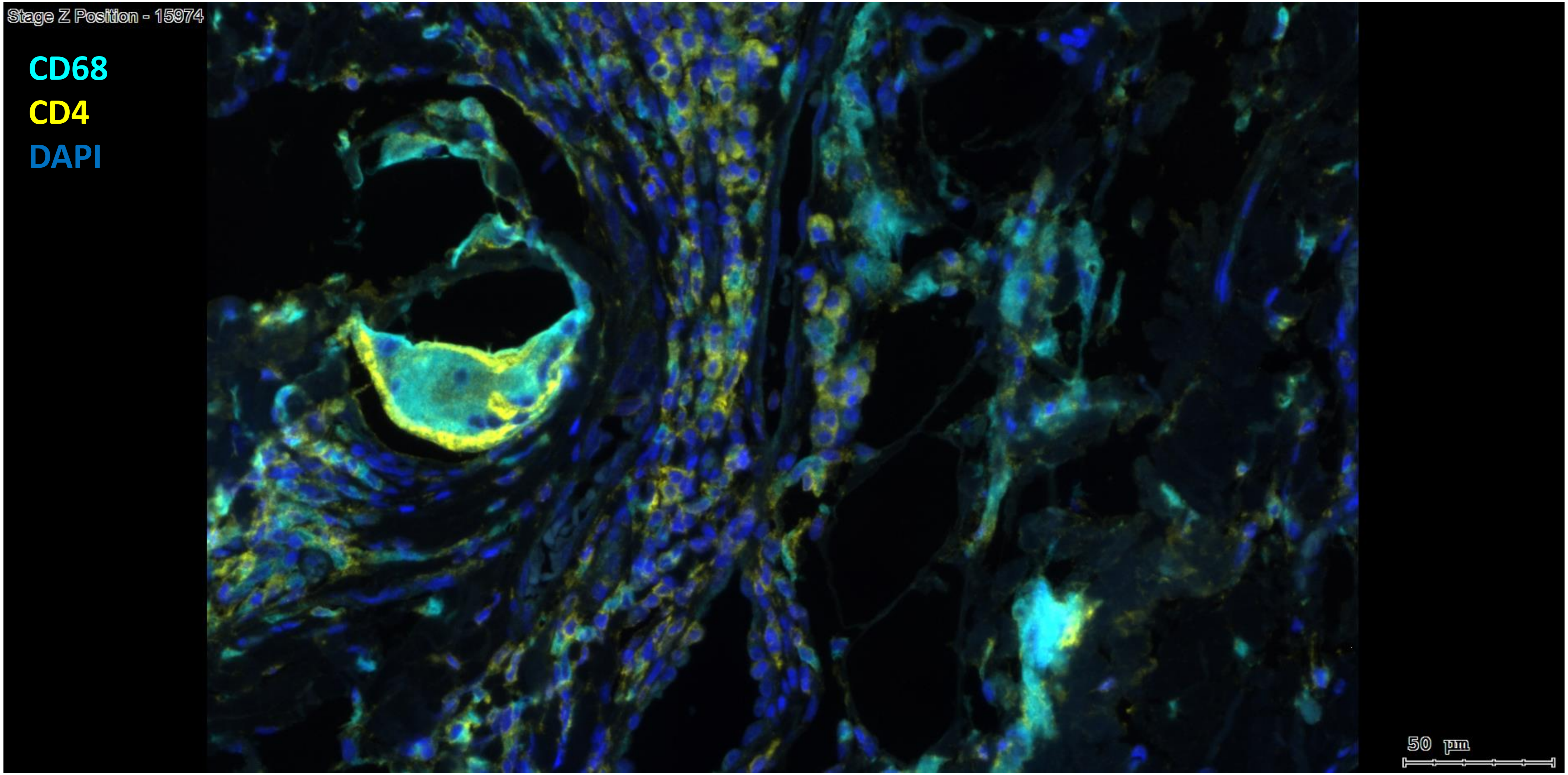

Figure 19: Macrophage labeling with CD68 (turquoise), labeling of T-helper cells with CD4 (yellow) and nuclei with DAPI (blue)

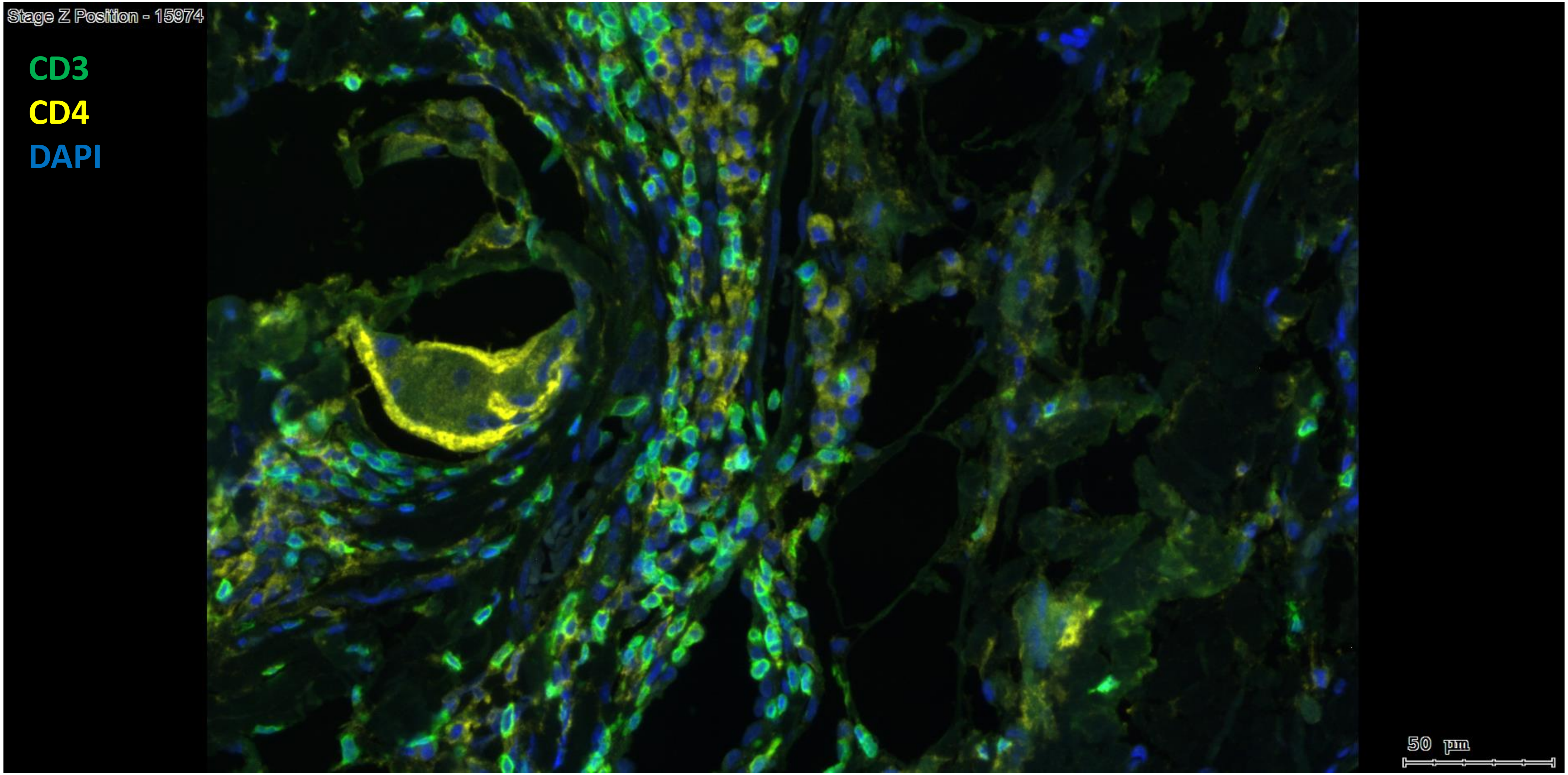

Figure 20: T-cell labeling with CD3 (green), labeling of T-helper cells with CD4 (yellow) and nuclei with DAPI (blue)

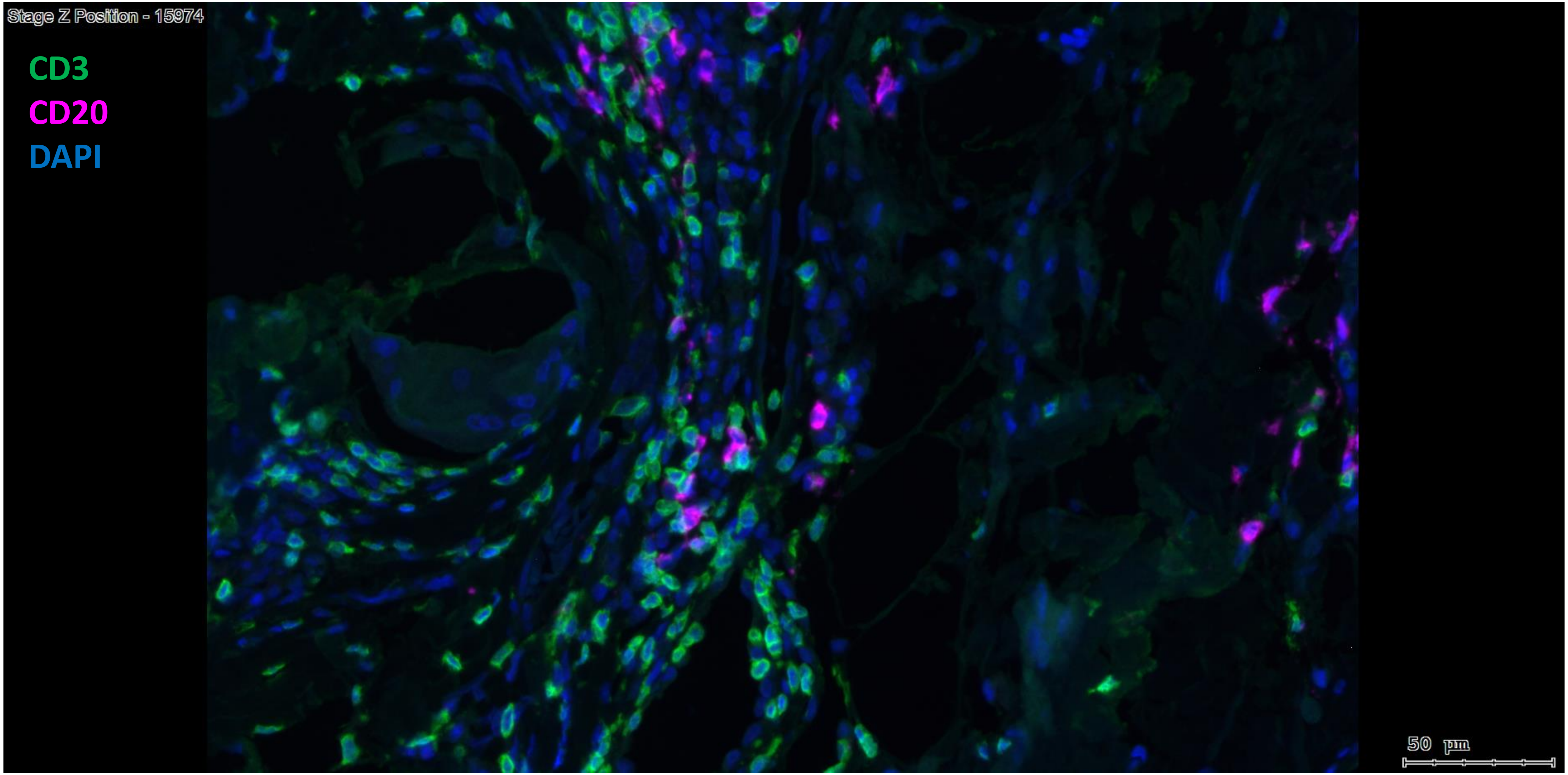

Figure 21: T-cell labeling with CD3 (green), labeling of B cells with CD20 (magenta) and nuclei with DAPI (blue)

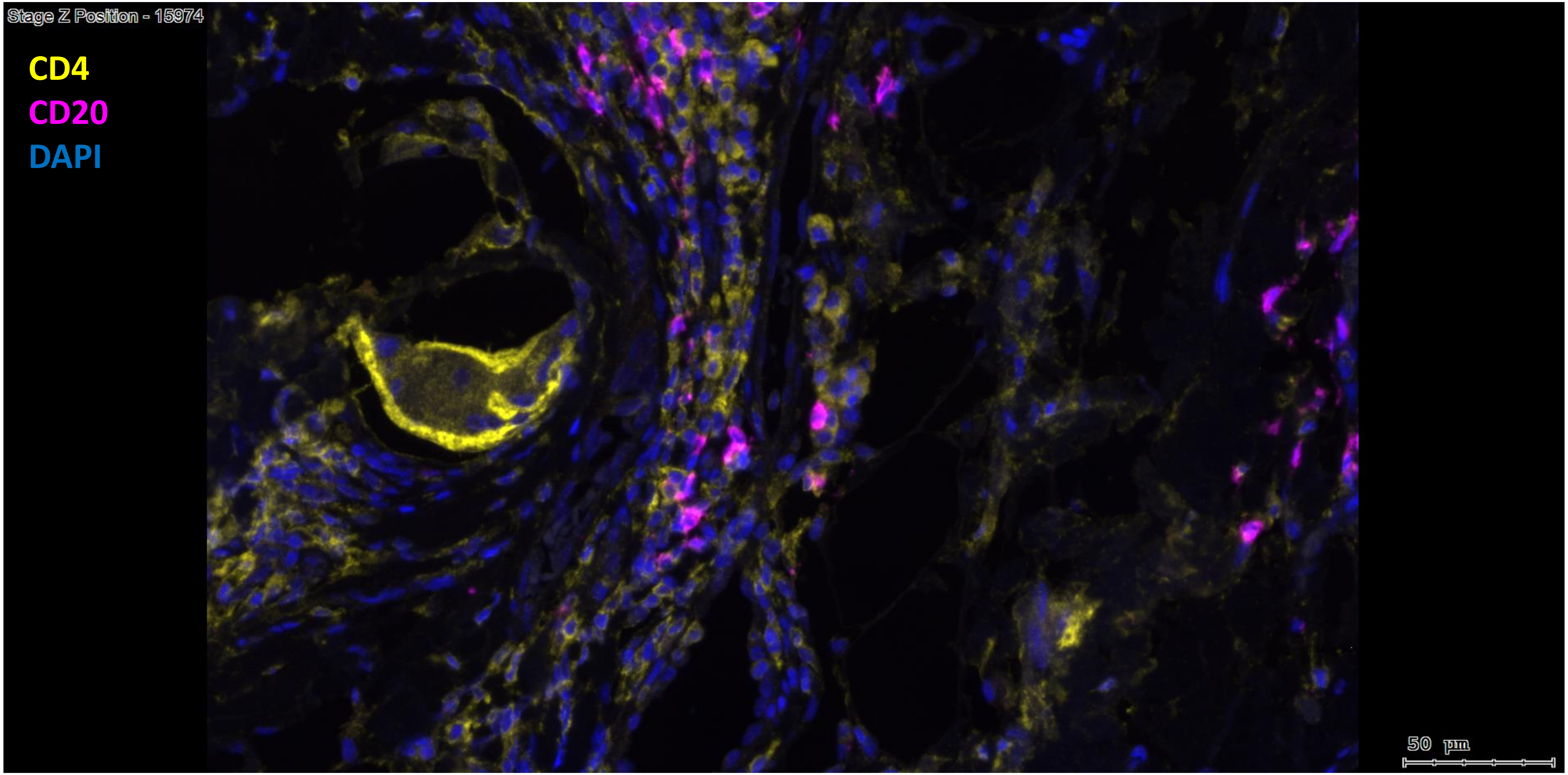

Figure 22: T-helper cell labeling with CD4 (yellow), labeling of B cells with CD20 (magenta) and nuclei with DAPI (blue)

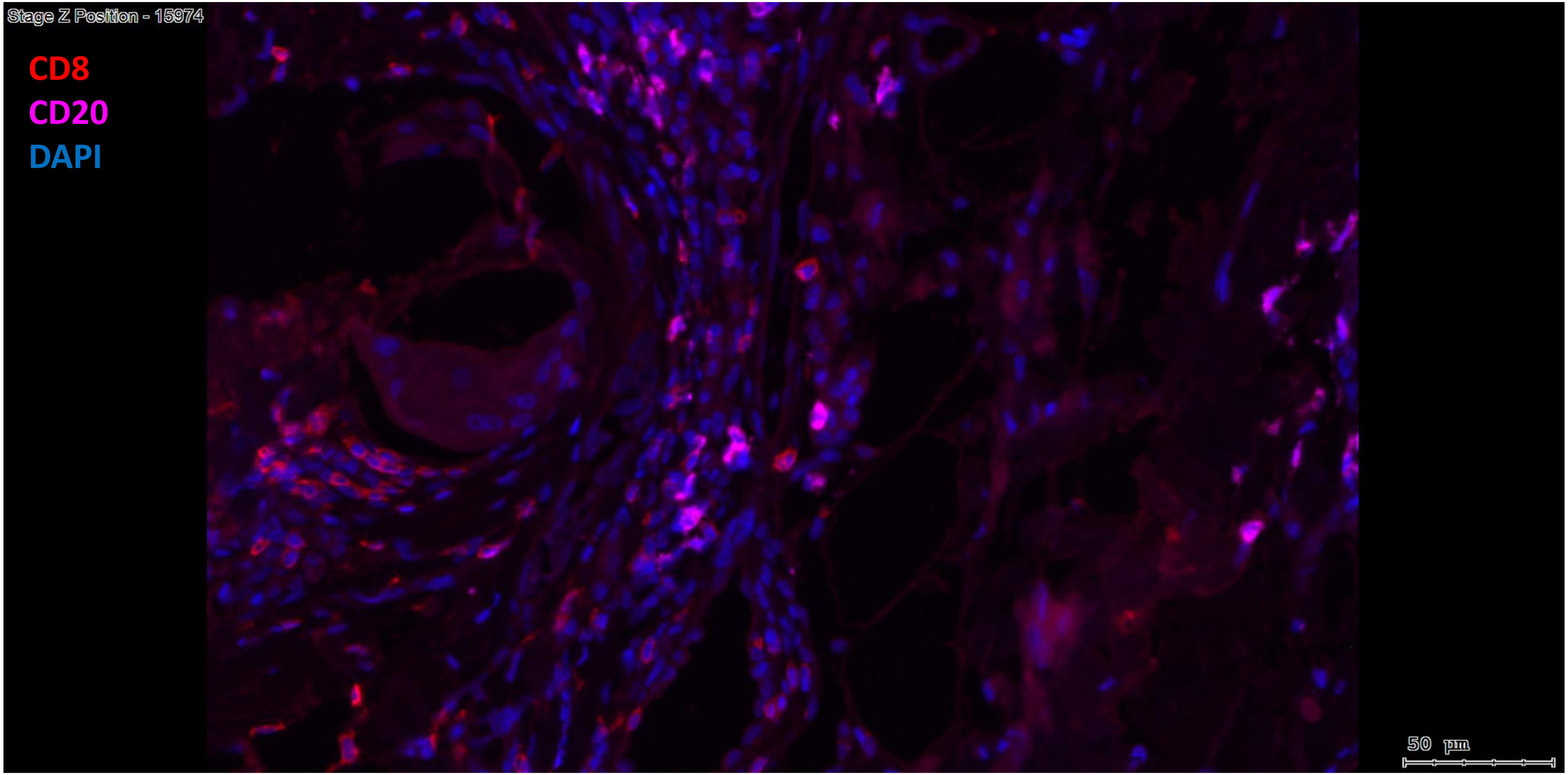

Figure 23: cytotoxic T-cell labeling with CD8 (red), labeling of B cells with CD20 (magenta) and nuclei with DAPI (blue)

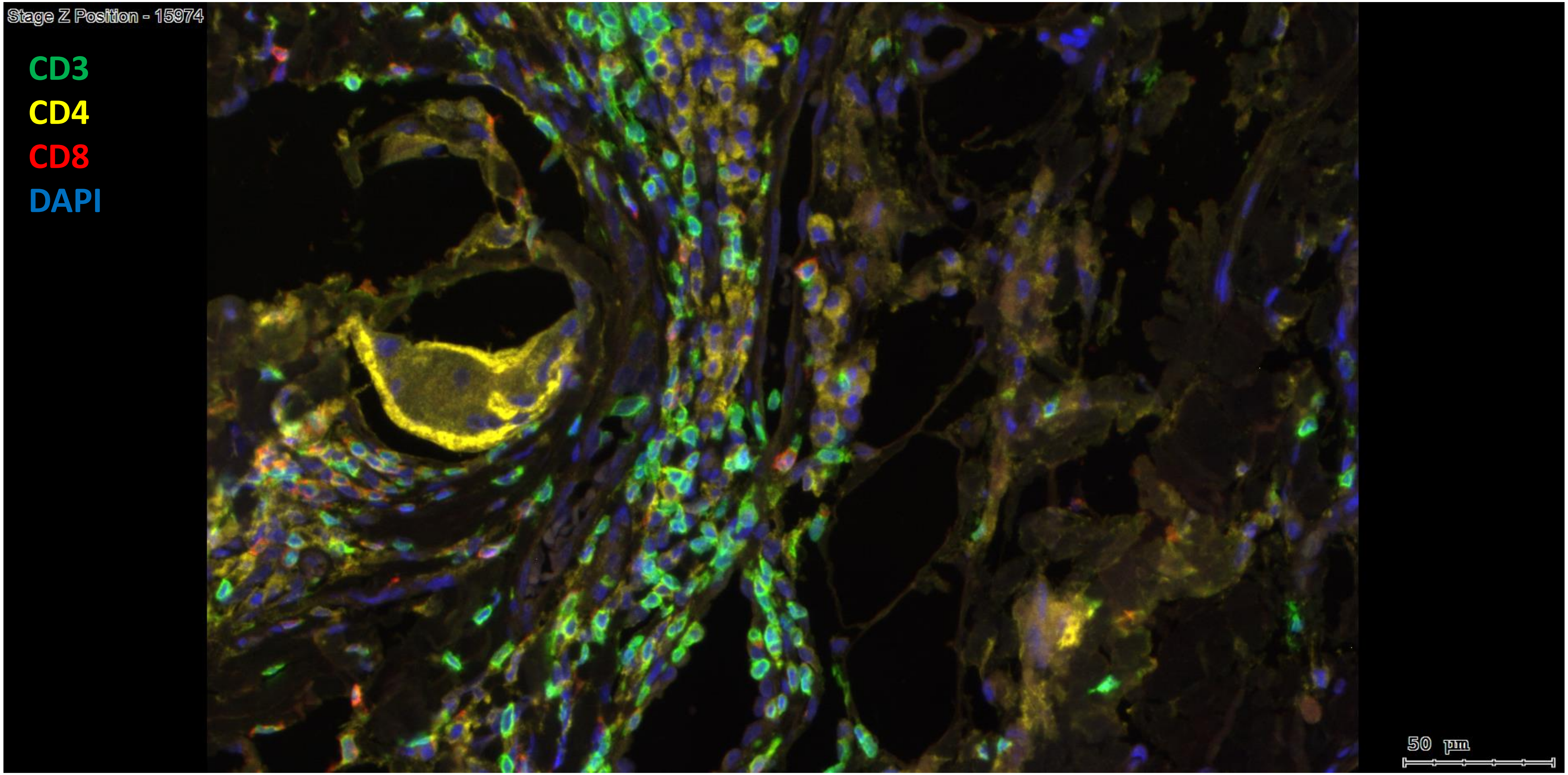

Figure 24: T-cell labeling with CD3 (green), labeling of T-helper cells with CD4 (yellow), cytotoxic T-cells with CD8 (red) and nuclei with DAPI (blue)

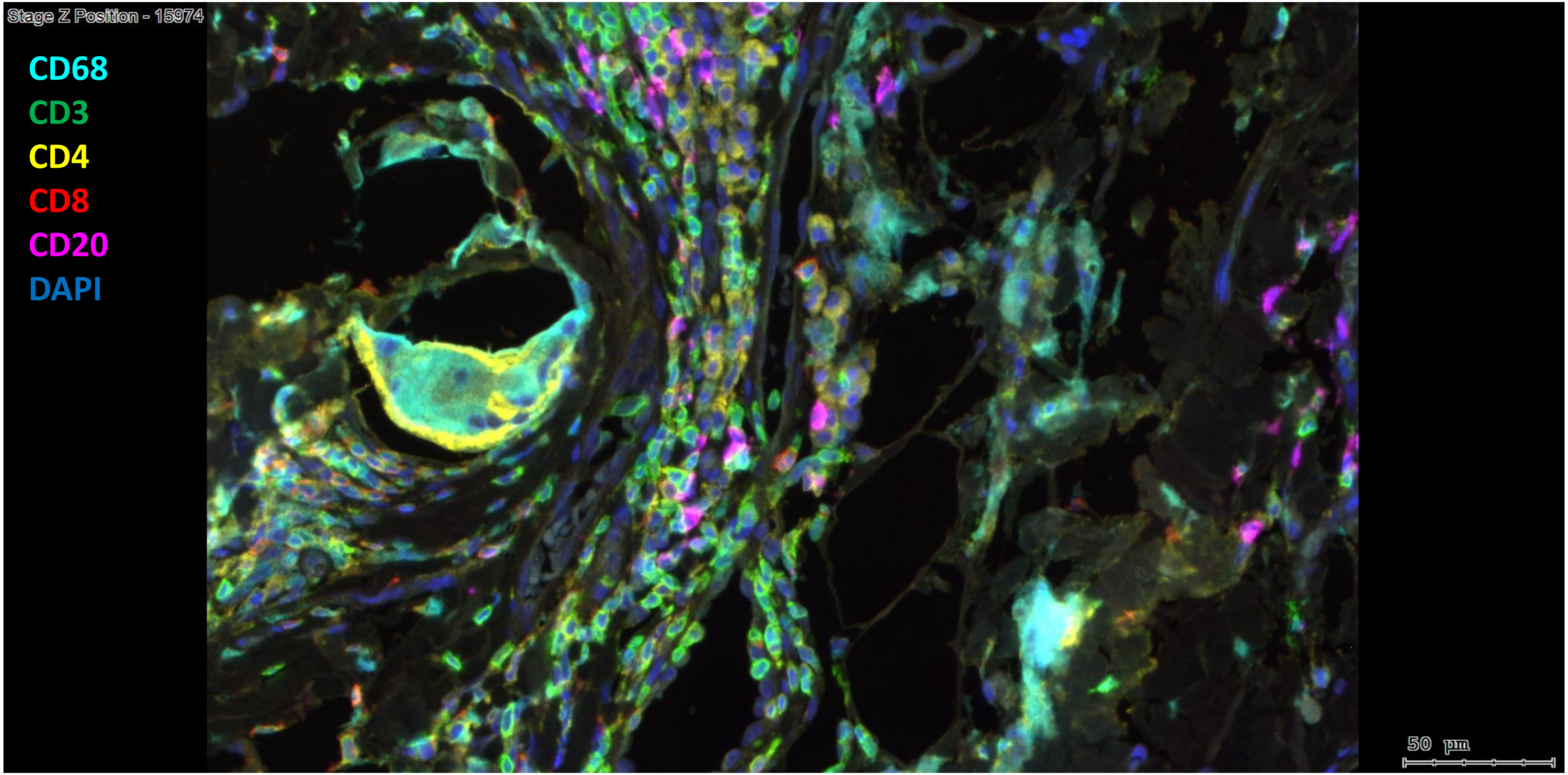

Figure 25: Labeling of macrophages with CD68 (turquoise), T cells with CD3 (green), T-helper cells with CD4 (yellow), cytotoxic T-cells with CD8 (red), B cells with CD20 (magenta) and nuclei with DAPI (blue)
