# Supplementary material for: Quantitative Characterization of Macrophage, Lymphocyte, and Neutrophil Subtypes Within the Foreign Body Granuloma of Human Mesh Explants by 5-Marker Multiplex Fluorescence Microscopy
Source: Front Med (Lausanne). 2022 Feb 15;9:777439. doi: 10.3389/fmed.2022.777439 (PMC8887619; doi:10.3389/fmed.2022.777439)
Supplement: Supplementary file 1 [file Data_Sheet_1.zip › Supplementary Material 4.pdf]

Supplement 4

Content

Macrophage panel

- Figure 1: Example region of interest with backward gating on DAPI image and linear distribution plots of the mean cellular intensities (p. 2)
- Figure 2: Six example regions of interest and linear distribution plots of the mean cellular intensities (p. 3)

Lymphocyte panel

- Figure 3: Example region of interest with backward gating on DAPI image and linear distribution plots of the mean cellular intensities (p. 4)
- Figure 4: Six example regions of interest and linear distribution plots of the mean cellular intensities (p. 5)

Neutrophil panel

- Figure 5: Example region of interest with backward gating on DAPI image and linear distribution plots of the mean cellular intensities (p. 6)
- Figure 6: Six example regions of and linear distribution plots of the mean cellular intensities (p. 7)

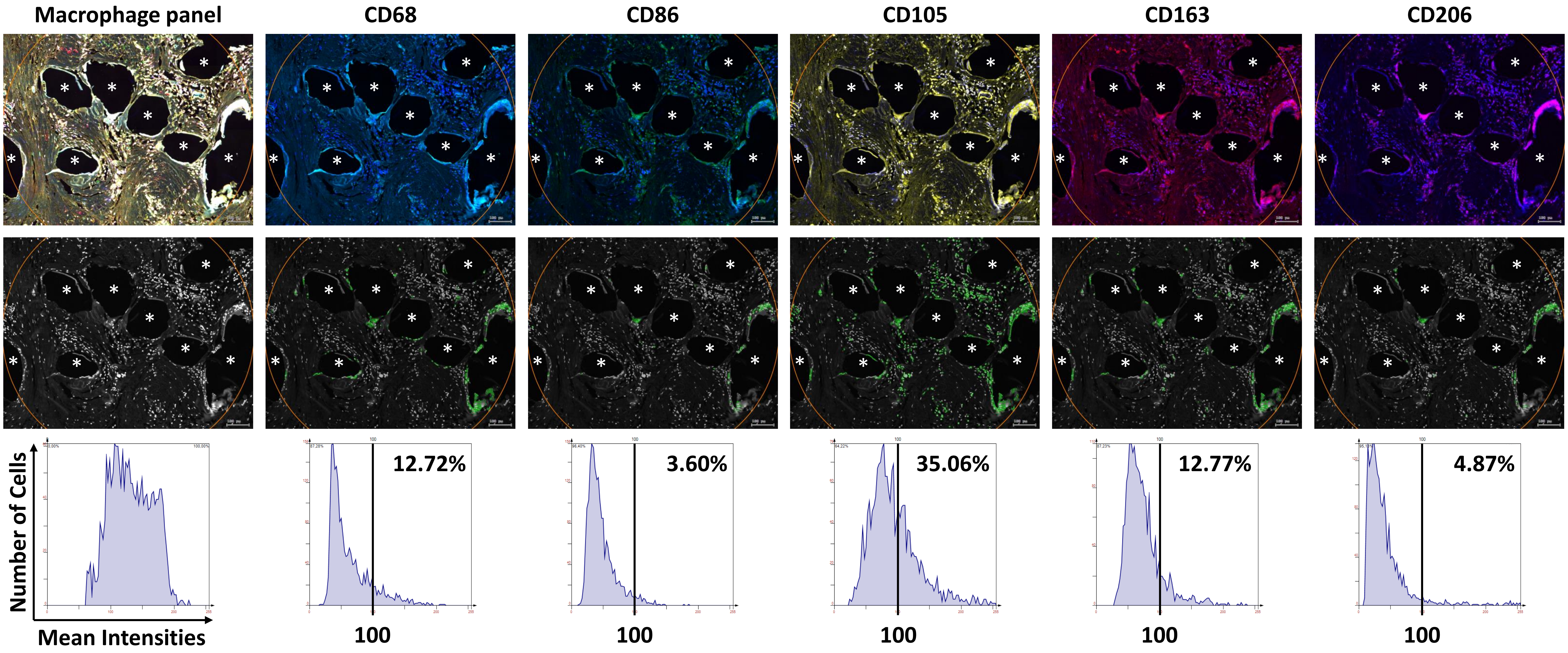

Figure 1: Example region of interest for the macrophage panel
Images in the top row from left to right: overlay of all markers, CD68 in turquois, CD86 in green, CD105 in yellow, CD163 in red, and CD206 in magenta. The individual marker images are all combined with the DAPI image in blue. The fiber locations are marked with asterisks.
Images in the middle: gray scale image of nucleus staining with DAPI on the left and corresponding backward gating images. In the backward gating images, “positive” cells determined by cut-off 100 are shown with green contours.
Images in the bottom row from left to right: linear distribution plots of the mean cellular intensities for DAPI and the respective markers with the mean intensity on the x-axis and the number of cells on the y-axis. Black lines indicate the cut-off 100. Percent of “positive” cells are displayed in the upper right corner.

As can be seen, the staining images (top row) correlate well with the backward gating images of the “positive” cells (middle row) determined by the cut-off 100. Looking at the individual channels, the background signal is low, but due to the additive overlay and the limitations of the RGB (Red, Green, Blue) color space, where turquoise, yellow, and magenta are mixed signals, the background signal appears much stronger in the overlay image (first image in the top row on the left).

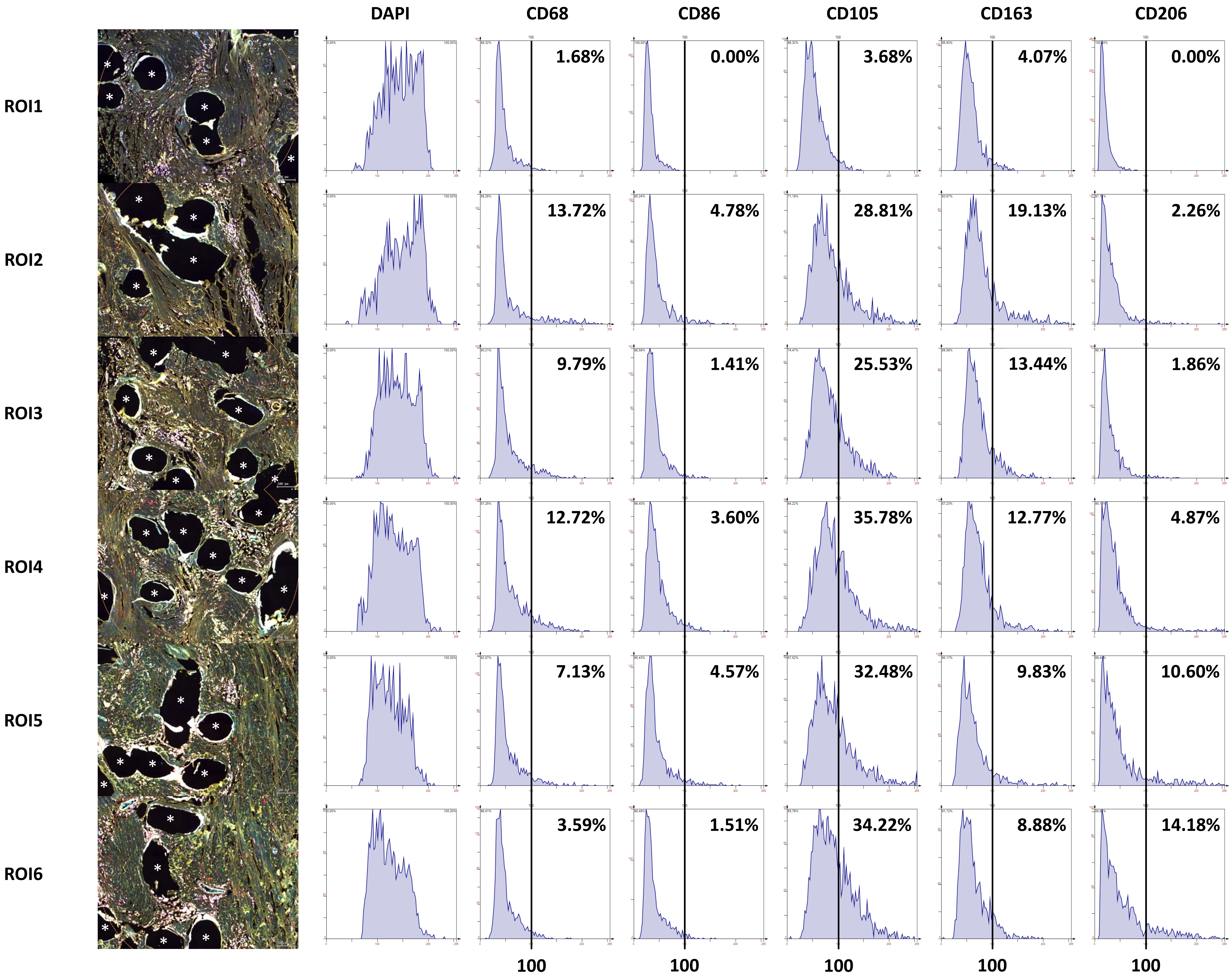

Figure 2: Six example regions of interest for the macrophage panel
In each row from left to right: overlay of all markers and linear distribution plots of the mean cellular intensities for DAPI, CD68, CD86, CD105, CD163, and CD206. Black lines indicate the cut-off 100. Percent of “positive” cells are displayed in the upper right corner. In the overlay images, the fiber locations are marked with asterisks.

The percentages of “positive” cells for each marker varies due to local differences in cellular composition and the number of fibers in the regions of interest.

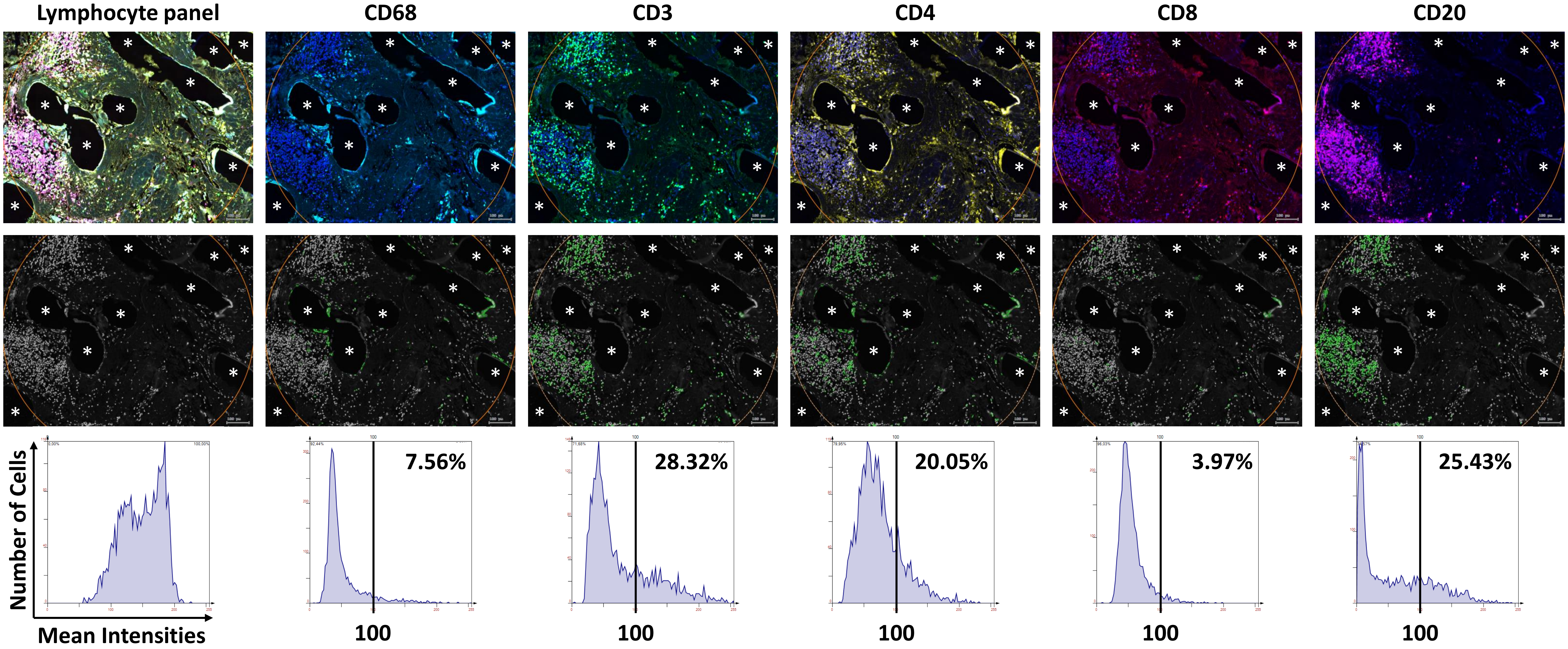

Figure 3: Example region of interest for the lymphocyte panel
Images in the top row from left to right: overlay of all markers, CD68 in turquois, CD3 in green, CD4 in yellow, CD8 in red, and CD20 in magenta. The individual marker images are all combined with the DAPI image in blue. The fiber locations are marked with asterisks.
Images in the middle: gray scale image of nucleus staining with DAPI on the left and corresponding backward gating images. In the backward gating images, "positive" cells determined by cut-off 100 are shown with green contours.
Images in the bottom row from left to right: linear distribution plots of the mean cellular intensities for DAPI and the respective markers with the mean intensity on the x-axis and the number of cells on the y-axis. Black lines indicate the cut-off 100. Percent of "positive" cells are displayed in the upper right corner.

As can be seen, the staining images (top row) correlate well with the backward gating images of the "positive" cells (middle row) determined by the cut-off 100. Looking at the individual channels, the background signal is low, but due to the additive overlay and the limitations of the RGB (Red, Green, Blue) color space, where turquoise, yellow, and magenta are mixed signals, the background signal appears much stronger in the overlay image (first image in the top row on the left).

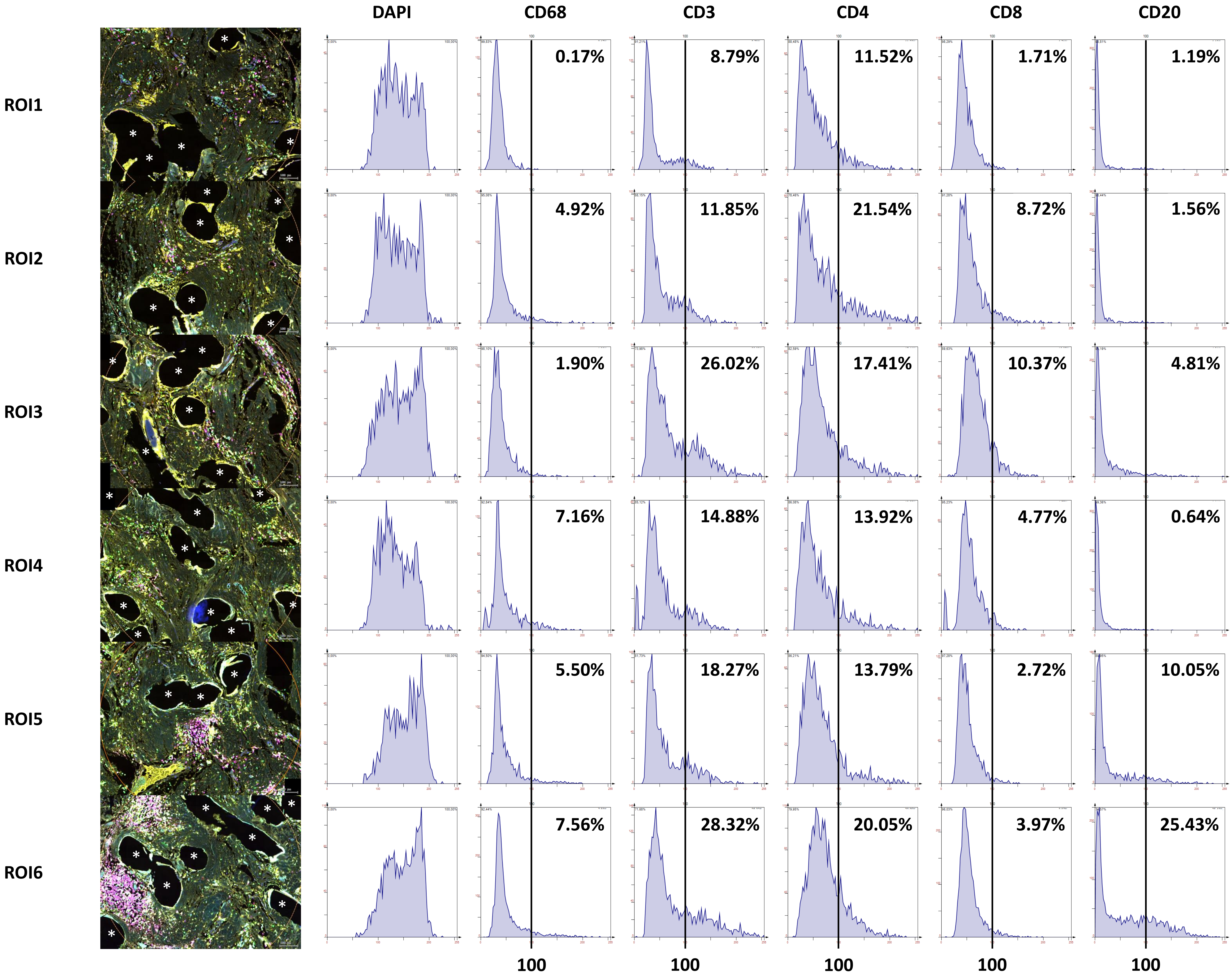

Figure 4: Six example regions of interest for the lymphocyte panel

In each row from left to right: overlay of all markers and linear distribution plots of the mean cellular intensities for DAPI, CD68, CD3, CD4, CD8, and CD20. Black lines indicate the cut-off 100. Percent of "positive" cells are displayed in the upper right corner. In the overlay images, the fiber locations are marked with asterisks.

The percentages of "positive" cells for each marker varies due to local differences in cellular composition, e.g., B cell clusters in regions of interest 5 and 6, and the number of fibers in the regions of interest.

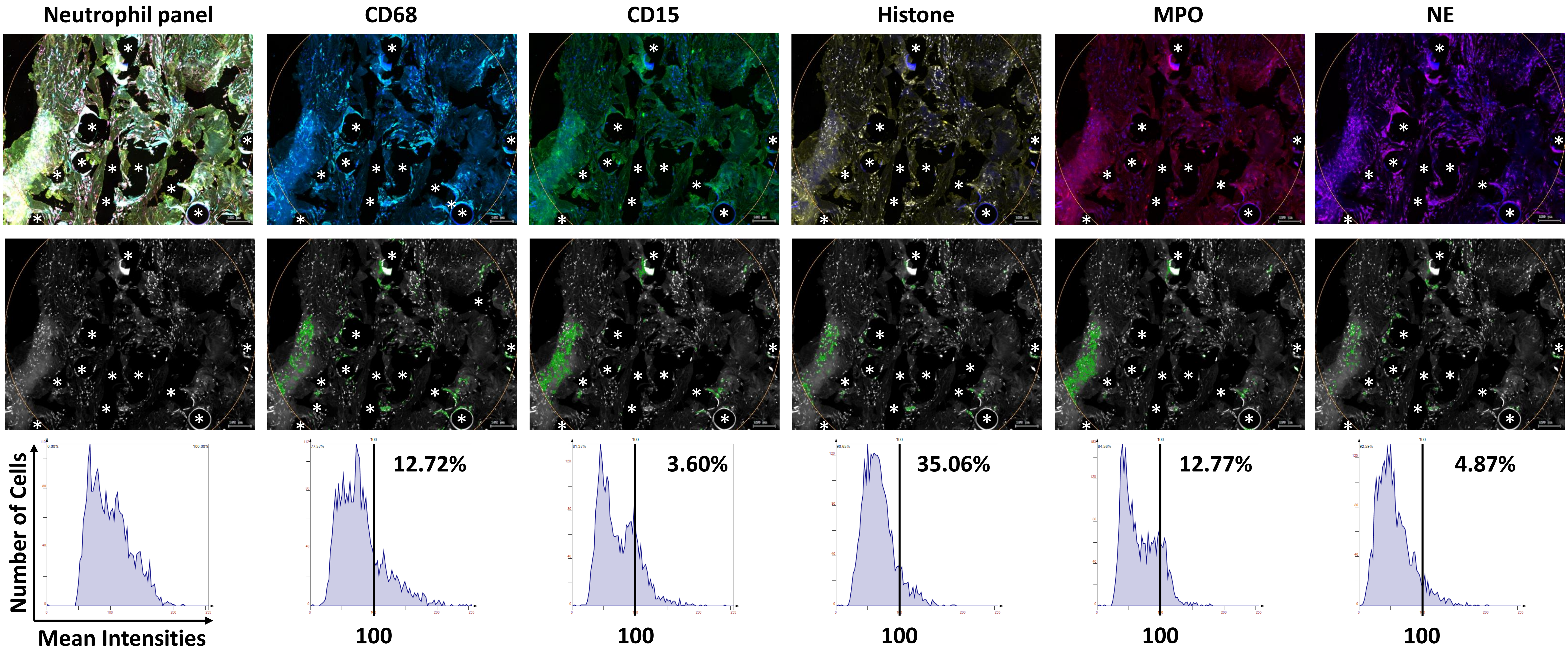

Figure 5: Example region of interest for the neutrophil panel
Images in the top row from left to right: overlay of all markers, CD68 in turquois, CD15 in green, Histone in yellow, MPO in red, and NE in magenta. The individual marker images are all combined with the DAPI image in blue. The fiber locations are marked with asterisks.
Images in the middle: gray scale image of nucleus staining with DAPI on the left and corresponding backward gating images. In the backward gating images, "positive" cells determined by cut-off 100 are shown with green contours.
Images in the bottom row from left to right: linear distribution plots of the mean cellular intensities for DAPI and the respective markers with the mean intensity on the x-axis and the number of cells on the y-axis. Black lines indicate the cut-off 100. Percent of "positive" cells are displayed in the upper right corner.

As can be seen, the staining images (top row) correlate well with the backward gating images of the "positive" cells (middle row) determined by the cut-off 100. Looking at the individual channels, the background signal is low, but due to the additive overlay and the limitations of the RGB (Red, Green, Blue) color space, where turquoise, yellow, and magenta are mixed signals, the background signal appears much stronger in the overlay image (first image in the top row on the left).

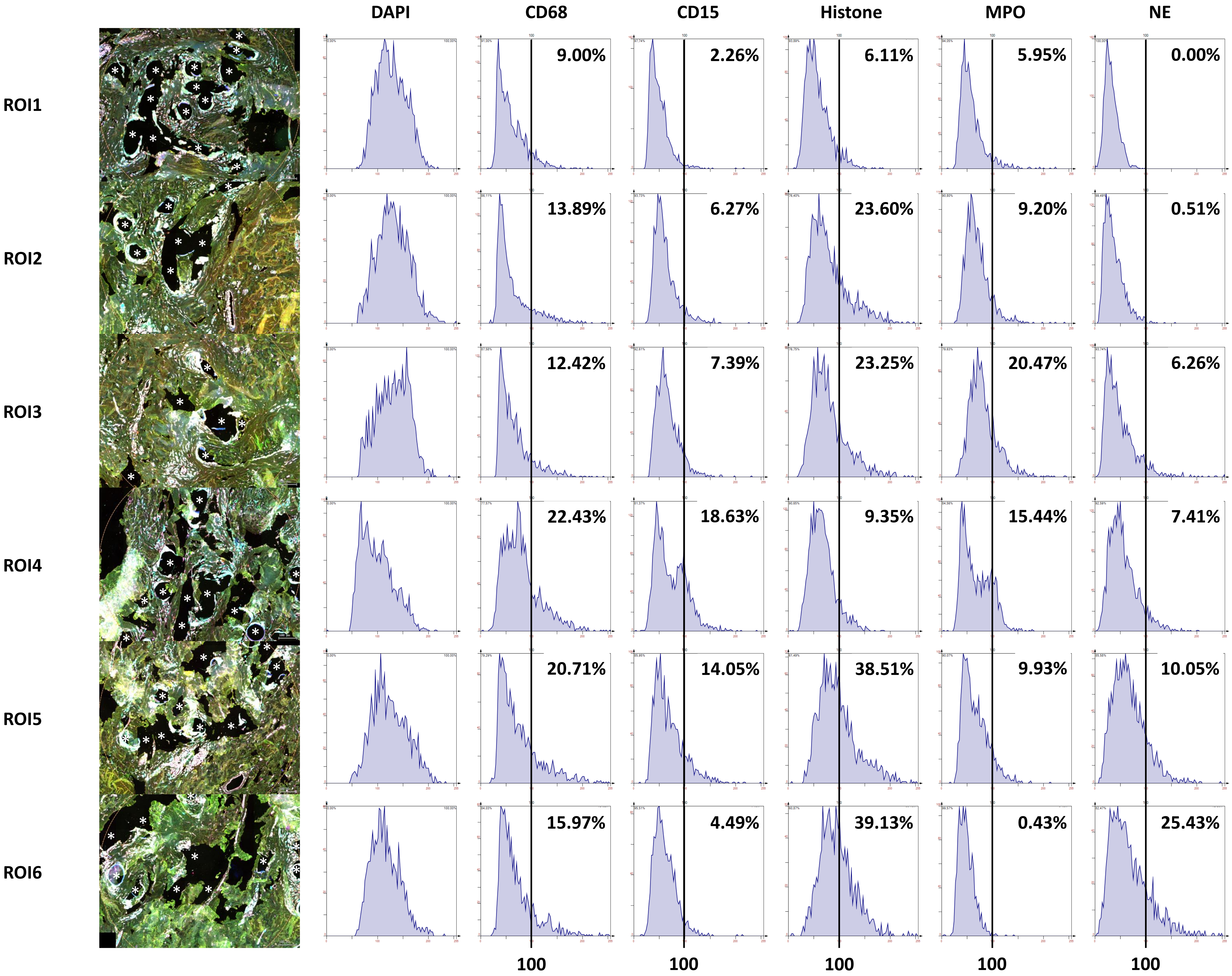

Figure 6: Six example regions of interest for the neutrophil panel

In each row from left to right: overlay of all markers and linear distribution plots for of the mean cellular intensities DAPI, CD68, CD15, Histone, MPO, and NE. Black lines indicate the cut-off 100. Percent of "positive" cells are displayed in the upper right corner. In the overlay images, the fiber locations are marked with asterisks.

The percentages of "positive" cells for each marker varies due to local differences in cellular composition and the number of fibers in the regions of interest.
