# Supplementary material for: Quantitative Characterization of Macrophage, Lymphocyte, and Neutrophil Subtypes Within the Foreign Body Granuloma of Human Mesh Explants by 5-Marker Multiplex Fluorescence Microscopy
Source: Front Med (Lausanne). 2022 Feb 15;9:777439. doi: 10.3389/fmed.2022.777439 (PMC8887619; doi:10.3389/fmed.2022.777439)
Supplement: Supplementary file 1 [file Data_Sheet_1.zip › Supplementary Material 5.pdf]

Supplement 5

Content

Positive predictive value

- Table 1: Positive predictive values (PPVs) for the various markers after applying different cut-off values (p. 2)

Macrophage panel

- Table 2: Cut-off variation (range: 110 – 70) for the macrophage panel at the example of one region of interest (p. 3)
- Figure 1: Effect of cut-off variation on the mean number of “positive” cells for the macrophage panel at an example of 6 regions of interest (p. 4)
- Table 3: Effect of cut-off variation on the mean number of “positive” cells for the macrophage panel at an example of 6 regions of interest (p. 4)

Lymphocyte panel

- Table 4: Cut-off variation (range: 110 – 70) for the lymphocyte panel at the example of one region of interest (p. 5)
- Figure 2: Effect of cut-off variation on the mean number of “positive” cells for the lymphocyte panel at an example of 6 regions of interest (p. 6)
- Table 5: Effect of cut-off variation on the mean number of “positive” cells for the lymphocyte panel at an example of 6 regions of interest (p. 6)

Table 1: Positive predictive values (PPVs) for the various markers after applying different cut-off values. Lowering the cut-off results in higher levels of co-expression, but also lower PPVs

**PPV**	=	**True Positives** / (**True Positives** + **False Positives**)
**True Positives**	=	Number of cells with a mean signal intensity > cut-off after labeling with specific monoclonal antibodies
**False Positive**	=	Number of cells with a mean signal intensity > cut-off after labeling with isotype control antibodies

Fluorophore	Filter	Marker	PPV			
			cut-off 100	cut-off 90	cut-off 80	cut-off 70
Opal 480	47 Cy	CD68	94.5%	90.3%	83.6%	72.3%
Opal 520	46 HE	CD86	92.8%	88.7%	82.4%	74.1%
Opal 570	43 HE	CD105	96.9%	94.0%	87.8%	76.9%
Opal 650	50 Cy	CD163	95.5%	92.8%	87.5%	80.0%
Opal 780	Cy 7E	CD206	99.8%	99.6%	99.3%	97.8%
Opal 480	47 Cy	CD68	93.9%	89.3%	81.9%	69.9%
Opal 520	46 HE	CD3	90.4%	85.1%	77.3%	67.6%
Opal 570	43 HE	CD4	96.3%	93.0%	85.9%	73.9%
Opal 650	50 Cy	CD8	95.1%	92.2%	86.6%	78.6%
Opal 780	Cy 7E	CD20	99.3%	98.9%	98.0%	94.1%
Opal 480	47 Cy	CD68	94.1%	89.6%	82.5%	70.7%
Opal 520	46 HE	CD15	89.1%	83.2%	74.7%	64.4%
Opal 570	43 HE	Histone	95.0%	90.5%	81.5%	67.2%
Opal 650	50 Cy	MPO	93.6%	90.0%	83.1%	73.7%
Opal 780	Cy 7E	NE	99.8%	99.6%	99.3%	97.8%
		**Overall**	95.1%	91.8%	86.1%	77.3%

Table 2: Cut-off variation (range: 110 – 70) for the macrophage panel at the example of one region of interest of a mesh plug. For each cut-off in relation to the given marker(s), the total number of detected “positive” cells, normalized to 2,000 cells, is displayed

Macrophage panel					Cut-off				
CD68	CD86	CD105	CD163	CD206	110	100	90	80	70
All “positive” cells for a given marker, independent of the other markers (n. d. = not defined, pos. = positive)									
pos.	n. d.	n. d.	n. d.	n. d.	195	254	356	454	595
n. d.	pos.	n. d.	n. d.	n. d.	38	72	112	169	249
n. d.	n. d.	pos.	n. d.	n. d.	530	716	925	1,156	1,453
n. d.	n. d.	n. d.	pos.	n. d.	183	255	385	583	886
n. d.	n. d.	n. d.	n. d.	pos.	83	97	120	150	209
All possible marker combinations (pos. = positive, neg. = negative)									
neg.	neg.	neg.	neg.	neg.	1,403	1,217	1,008	773	477
pos.	neg.	neg.	neg.	neg.	54	51	52	48	38
neg.	pos.	neg.	neg.	neg.	4	5	4	5	11
neg.	neg.	pos.	neg.	neg.	318	429	500	534	523
neg.	neg.	neg.	pos.	neg.	5	4	4	13	7
neg.	neg.	neg.	neg.	pos.	0	0	0	0	0
pos.	pos.	neg.	neg.	neg.	0	0	0	0	0
pos.	neg.	pos.	neg.	neg.	27	36	45	52	57
pos.	neg.	neg.	pos.	neg.	4	7	7	5	14
pos.	neg.	neg.	neg.	pos.	0	0	0	0	0
neg.	pos.	pos.	neg.	neg.	0	0	3	4	6
neg.	pos.	neg.	pos.	neg.	0	0	0	0	0
neg.	pos.	neg.	neg.	pos.	0	0	0	0	0
neg.	neg.	pos.	pos.	neg.	52	67	105	188	339
neg.	neg.	pos.	neg.	pos.	5	4	2	0	1
neg.	neg.	neg.	pos.	pos.	0	0	0	0	0
pos.	pos.	pos.	neg.	neg.	0	0	1	1	0
pos.	pos.	neg.	pos.	neg.	0	0	0	0	0
pos.	pos.	neg.	neg.	pos.	0	0	0	0	0
pos.	neg.	pos.	pos.	neg.	41	74	126	174	230
pos.	neg.	pos.	neg.	pos.	5	1	0	0	1
pos.	neg.	neg.	pos.	pos.	0	0	0	0	0
neg.	pos.	pos.	pos.	neg.	0	0	0	4	9
neg.	pos.	pos.	neg.	pos.	0	0	0	0	0
neg.	pos.	neg.	pos.	pos.	0	0	0	0	0
neg.	neg.	pos.	pos.	pos.	16	14	15	22	29
pos.	pos.	pos.	pos.	neg.	9	12	26	49	80
pos.	pos.	pos.	neg.	pos.	0	1	1	0	0
pos.	pos.	neg.	pos.	pos.	0	0	0	0	0
pos.	neg.	pos.	pos.	pos.	32	23	25	21	34
neg.	pos.	pos.	pos.	pos.	2	5	3	2	2
pos.	pos.	pos.	pos.	pos.	22	49	73	104	141

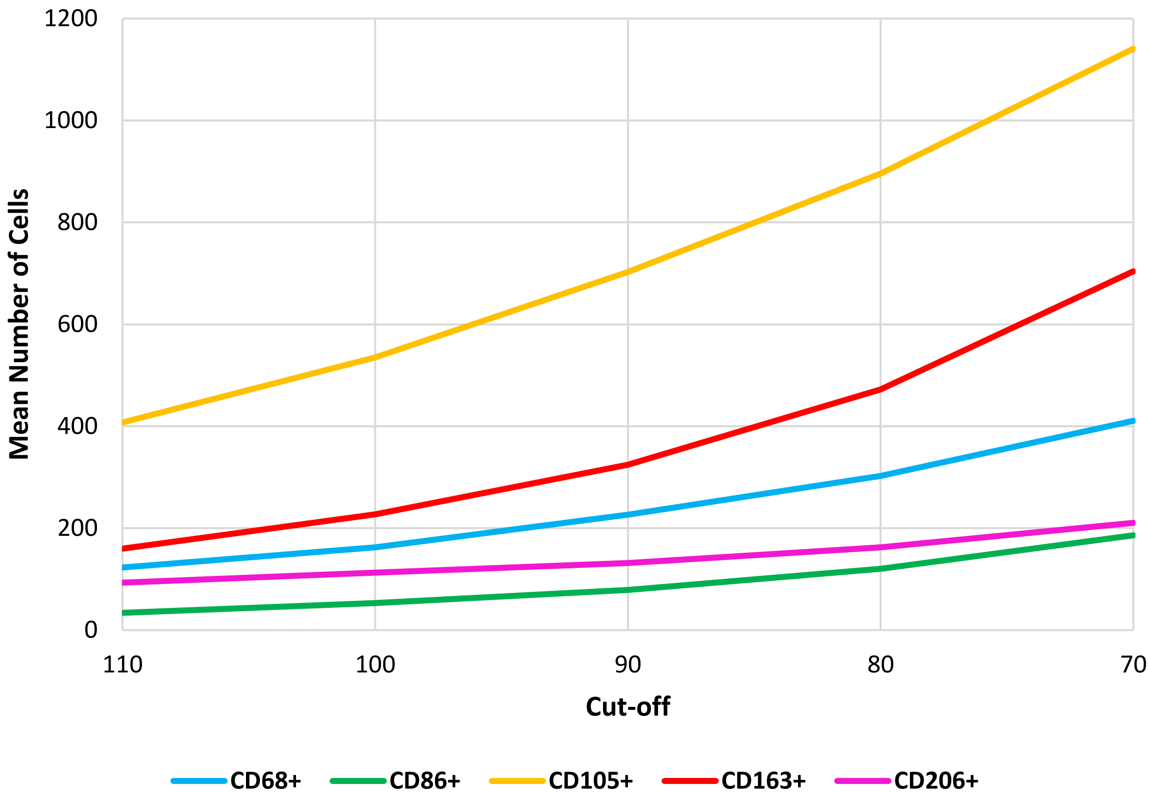

Figure 1: Effect of cut-off variation on the mean number of "positive" cells for the macrophage panel at an example of 6 regions of interest of a mesh plug. The mean number of cells is normalized to 2,000 cells.

Table 3: Effect of cut-off variation on the mean number of "positive" cells for the macrophage panel at an example of 6 regions of interest of a mesh plug. For each cut-off, the mean number of cells is normalized to 2,000 cells.

**Macrophage panel**					**Cut-off**				
CD68	CD86	CD105	CD163	CD206	110	100	90	80	70
All "positive" cells for a given marker, independent of the other markers (n. d. = not defined, pos. = positive)									
pos.	n. d.	n. d.	n. d.	n. d.	123 (86)	162 (97)	226 (109)	303 (123)	411 (139)
n. d.	pos.	n. d.	n. d.	n. d.	34 (30)	53 (39)	79 (52)	121 (67)	186 (87)
n. d.	n. d.	pos.	n. d.	n. d.	407 (194)	535 (238)	703 (298)	896 (347)	1,141 (390)
n. d.	n. d.	n. d.	pos.	n. d.	160 (82)	227 (101)	324 (129)	472 (174)	704 (248)
n. d.	n. d.	n. d.	n. d.	pos.	93 (95)	113 (112)	132 (122)	162 (142)	210 (179)

Table 4: Cut-off variation (range: 110 – 70) for the lymphocyte panel at the example of one region of interest of a mesh plug. For each cut-off in relation to the given marker(s), the total number of detected "positive" cells, normalized to 2,000 cells, is displayed.

Lymphocyte panel					Cut-off				
CD68	CD3	CD4	CD8	CD20	110	100	90	80	70
All "positive" cells for a given marker, independent of the other markers (n. d. = not defined, pos. = positive)									
pos.	n. d.	n. d.	n. d.	n. d.	84	110	147	185	266
n. d.	pos.	n. d.	n. d.	n. d.	270	365	457	518	594
n. d.	n. d.	pos.	n. d.	n. d.	201	276	363	500	678
n. d.	n. d.	n. d.	pos.	n. d.	31	54	90	159	287
n. d.	n. d.	n. d.	n. d.	pos.	160	201	258	298	348
All possible marker combinations (pos. = positive, neg. = negative)									
neg.	neg.	neg.	neg.	neg.	1,395	1,238	1,068	917	768
pos.	neg.	neg.	neg.	neg.	12	11	18	26	39
neg.	pos.	neg.	neg.	neg.	215	262	273	242	175
neg.	neg.	pos.	neg.	neg.	103	118	136	171	181
neg.	neg.	neg.	pos.	neg.	4	1	0	4	5
neg.	neg.	neg.	neg.	pos.	137	151	179	186	173
pos.	pos.	neg.	neg.	neg.	0	0	0	1	0
pos.	neg.	pos.	neg.	neg.	69	90	109	113	113
pos.	neg.	neg.	pos.	neg.	0	0	0	1	3
pos.	neg.	neg.	neg.	pos.	0	0	0	0	3
neg.	pos.	pos.	neg.	neg.	16	31	56	87	120
neg.	pos.	neg.	pos.	neg.	22	37	60	84	107
neg.	pos.	neg.	neg.	pos.	11	20	31	27	30
neg.	neg.	pos.	pos.	neg.	0	1	3	4	14
neg.	neg.	pos.	neg.	pos.	5	16	19	33	39
neg.	neg.	neg.	pos.	pos.	0	0	1	1	0
pos.	pos.	pos.	neg.	neg.	0	1	4	5	8
pos.	pos.	neg.	pos.	neg.	0	0	1	0	3
pos.	pos.	neg.	neg.	pos.	0	0	0	0	0
pos.	neg.	pos.	pos.	neg.	1	7	7	20	57
pos.	neg.	pos.	neg.	pos.	1	0	3	4	10
pos.	neg.	neg.	pos.	pos.	0	0	0	0	0
neg.	pos.	pos.	pos.	neg.	1	1	4	15	35
neg.	pos.	pos.	neg.	pos.	3	7	15	29	53
neg.	pos.	neg.	pos.	pos.	3	4	5	10	16
neg.	neg.	pos.	pos.	pos.	0	0	0	0	1
pos.	pos.	pos.	pos.	neg.	0	0	4	11	24
pos.	pos.	pos.	neg.	pos.	0	0	0	0	3
pos.	pos.	neg.	pos.	pos.	0	0	0	0	0
pos.	neg.	pos.	pos.	pos.	0	1	1	1	1
neg.	pos.	pos.	pos.	pos.	0	1	3	5	16
pos.	pos.	pos.	pos.	pos.	0	0	0	1	3

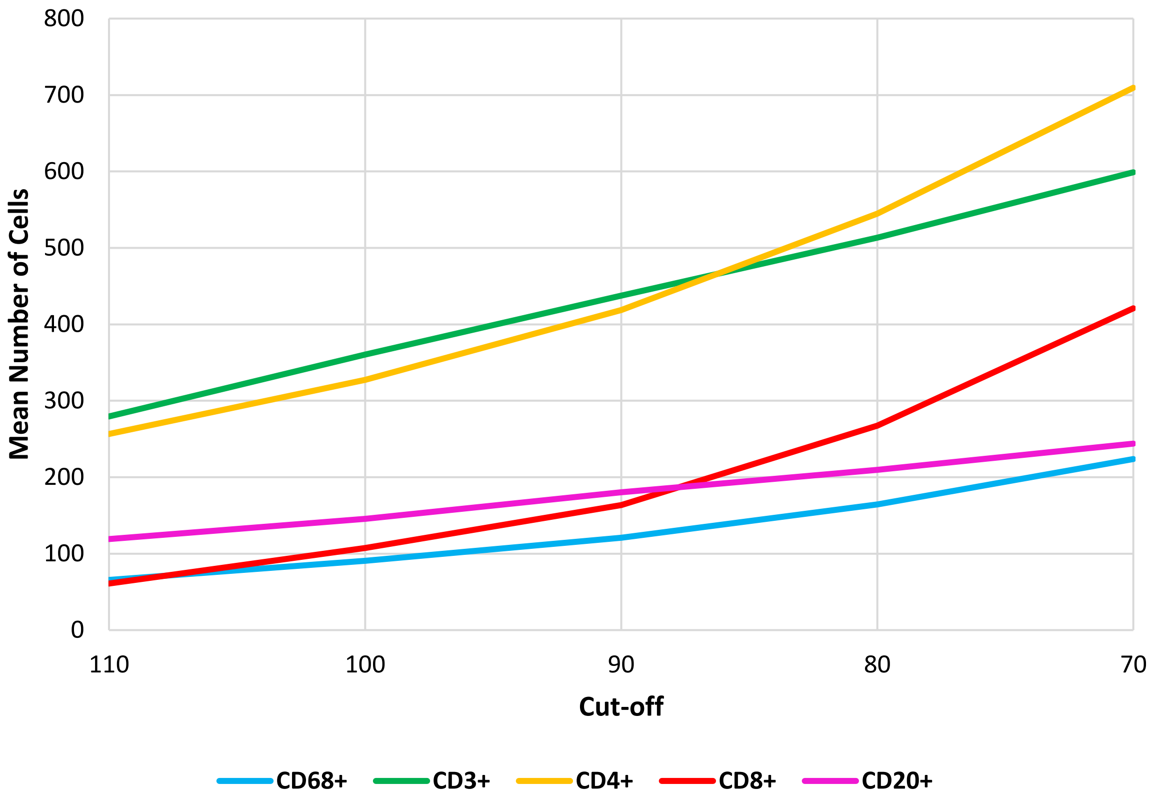

Figure 2: Effect of cut-off variation on the mean number of "positive" cells for the lymphocyte panel at an example of 6 regions of interest of a mesh plug. The mean number of cells is normalized to 2,000 cells.

Table 5: Effect of cut-off variation on the mean number of "positive" cells for the lymphocyte panel at an example of 6 regions of interest of a mesh plug. For each cut-off, the mean number of cells is normalized to 2,000 cells.

Lymphocyte panel					Cut-off				
CD68	CD3	CD4	CD8	CD20	110	100	90	80	70
All "positive" cells for a given marker, independent of the other markers (n. d. = not defined, pos. = positive)									
pos.	n. d.	n. d.	n. d.	n. d.	66 (44)	91 (59)	121 (73)	164 (92)	224 (114)
n. d.	pos.	n. d.	n. d.	n. d.	280 (155)	360 (156)	438 (156)	513 (162)	599 (175)
n. d.	n. d.	pos.	n. d.	n. d.	257 (68)	327 (79)	419 (89)	545 (112)	710 (142)
n. d.	n. d.	n. d.	pos.	n. d.	61 (45)	108 (69)	164 (98)	268 (154)	421 (219)
n. d.	n. d.	n. d.	n. d.	pos.	119 (162)	146 (191)	181 (223)	209 (247)	244 (276)
