# Supplementary figures and images for: Quantitative Characterization of Macrophage, Lymphocyte, and Neutrophil Subtypes Within the Foreign Body Granuloma of Human Mesh Explants by 5-Marker Multiplex Fluorescence Microscopy
Source: Front Med (Lausanne). 2022 Feb 15;9:777439. doi: 10.3389/fmed.2022.777439 (PMC8887619; doi:10.3389/fmed.2022.777439)

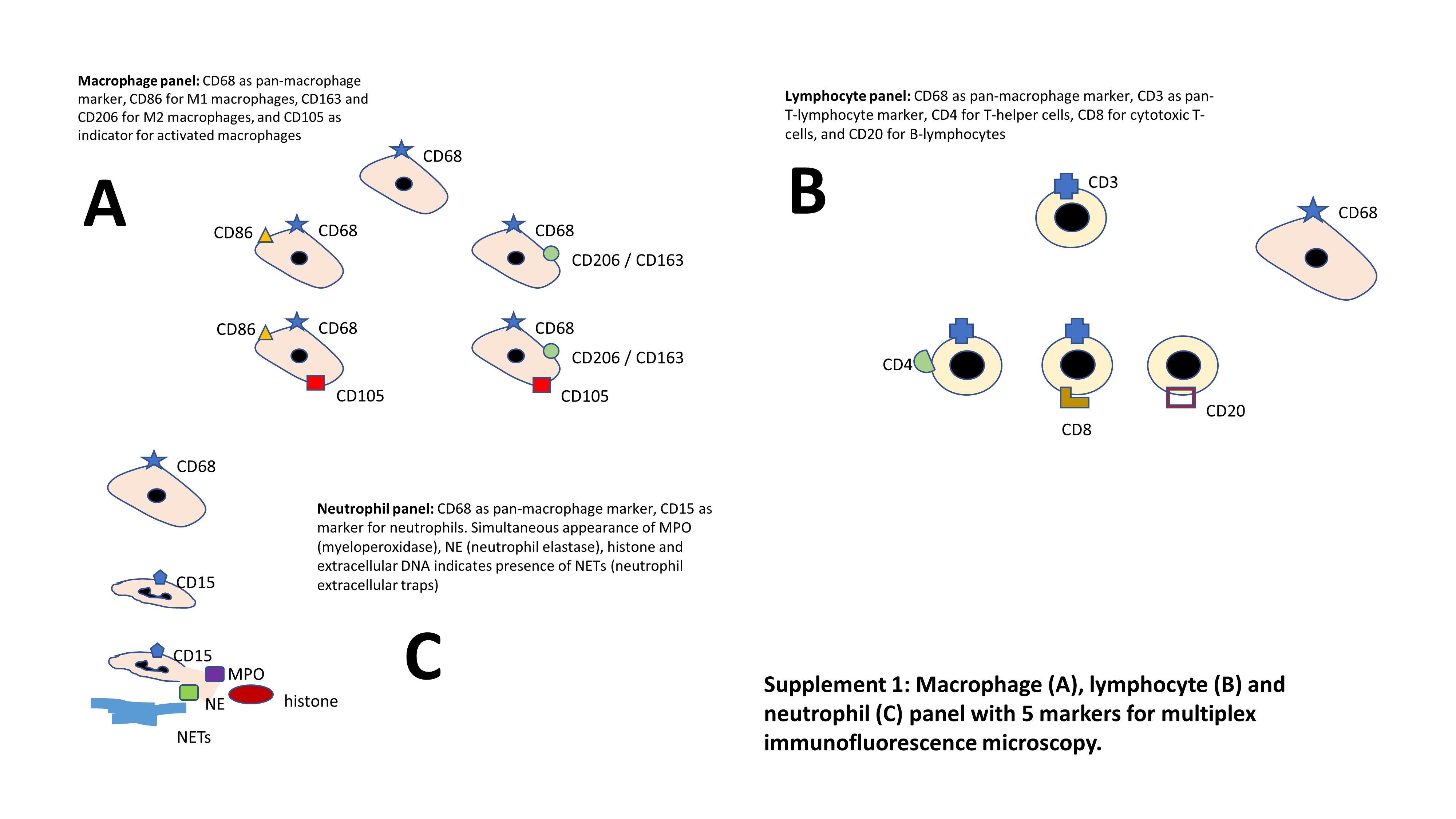

Supplement: Supplementary file 1 [file Data_Sheet_1.zip › Supplementary Material 1.JPEG]

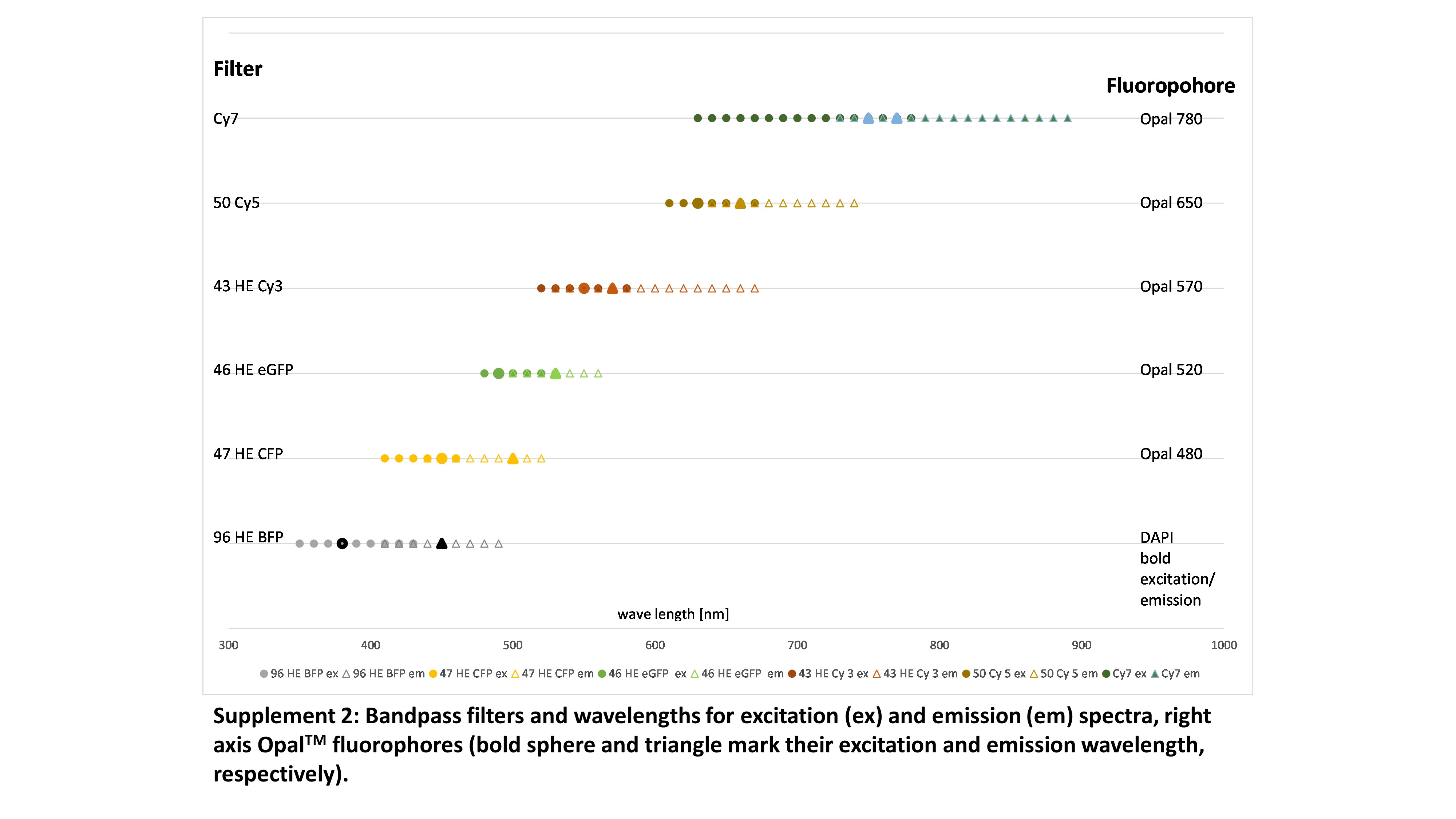

Supplement: Supplementary file 1 [file Data_Sheet_1.zip › Supplementary Material 2.JPEG]
